# A Transient, Highly Reactive Fe(IV)=O Species Revealed Through the Interference by O_2_ in the Activation of Organic Peracids by [(N4Py)Fe(II)]^2+^

**DOI:** 10.1021/acscatal.5c00706

**Published:** 2025-04-21

**Authors:** Marika Di Berto Mancini, C. Maurits de Roo, Andy S. Sardjan, Ronald Hage, Giorgio Olivo, Osvaldo Lanzalunga, Marcel Swart, Wesley R. Browne

**Affiliations:** † Stratingh Institute for Chemistry, Faculty of Science and Engineering, University of Groningen, Nijenborgh 3, Groningen 9474AG, Netherlands; ‡ Dipartimento di Chimica and Istituto CNR per i Sistemi Biologici (ISB-CNR), Università di Roma “La Sapienza”, P.le A. Moro, 5, Rome I-00185, Italy; § IQCC and Department of Chemistry, Universitat de Girona, ParcUdG, c/Emili Grahit 91, Girona 17003, Spain; ∥ ICREA, Pg. Lluís Companys 23, Barcelona 08010, Spain

**Keywords:** iron, peracid, spectroscopy, mechanism, oxidation, catalysis

## Abstract

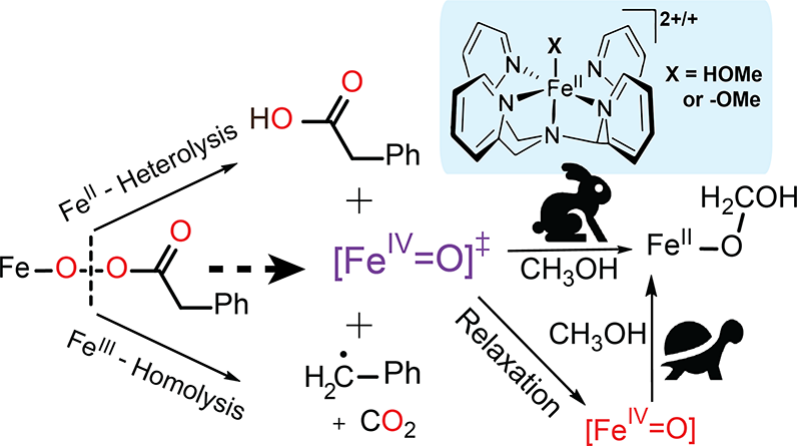

Biomimetic models of high-valent species relevant to those formed by the activation of O_2_ by nonheme iron enzymes are essential for understanding reactivity. In synthetic complexes, oxidants such as peroxides and peroxyacids rather than O_2_, are used to generate these species. However, although O_2_ is not the terminal oxidant in these models, its presence in reaction mixtures can negatively impact the outcome of catalytic reactions. In this report, the origin of this impact is elucidated using the reaction of the nonheme iron complex [(N4Py)Fe(II)(CH_3_CN)]^2+^ (**1**, N4Py = *N,N*-bis(2-pyridylmethyl)-*N*-bis(2-pyridyl)methylamine) with phenyl peracetic acid. We show that the speciation of the catalyst is sensitive to changes in composition by monitoring reaction progress using multiple *operando* spectroscopic techniques (UV/vis, Raman, FTIR, luminescence spectroscopy) concurrently to track changes in concentrations of the iron complexes, organic compounds, and gases. We elucidate the fundamental role played by molecular oxygen in the observed progress of the reactions, affecting the product distribution as expected, but also driving the system toward the accumulation of Fe(IV)=O species by scavenging intermediate benzyl radicals. These reactions influence the operation of a Fe(II)/Fe(IV) catalytic cycle with the peracid. An unexpected outcome of the study is that the data strongly indicate the transient formation of a highly reactive iron species capable of oxidizing organic substrates (e.g., methanol to methanal) within the solvent cage. We show that it is this species that enables an Fe(II)/Fe(IV) catalytic cycle. These findings shed light on differences in the catalytic performance of biomimetic nonheme iron complexes compared to the enzymes that inspire them.

## Introduction

Nonheme iron enzymes, such as methane monooxygenase (MMO) and Tau-D, as well as the antibiotic Fe-bleomycin (FeBLM), activate dioxygen (O_2_) to catalyze oxidative transformations.^1^ Over the past decades, biomimetic analogues of these nonheme iron-dependent enzymes have been studied intensively to model their activity and ultimately realize fully synthetic systems that match their performance in oxidative transformations. Both enzymes and biomimetic models generate key reactive intermediates, high-valent iron(IV)–oxo species, through activation by an oxidant. Hence, the investigation of the structure, spectroscopy, and reactivity of these intermediates has been a major focus of attention.^2^ Oxidation of organic substrates by Fe(IV)=O species and analysis of proposed intermediates relevant to the O_2_ activation pathways in enzymes generally rely on oxidants other than O_2_.^3,4^ Oxygen atom transfer reagents, such as hydroperoxides^5−7^ (e.g., H_2_O_2_ and alkyl hydroperoxides), PhIO, peroxy acids (i.e., RCO_3_H),^8^ NaO*X* (*X* = Cl or Br),^9,10^ and O_3_,^11^ have been employed to generate Fe(IV)=O species supported by various aminopyridyl ligands under a wide range of reaction conditions, including in water at room temperature.^12^

Catalytic oxidation of C–H bonds occurs via initial hydrogen atom abstraction (HAT) by the Fe(IV)=O species, forming a C-centered radical and an Fe(III)–OH complex, followed by OH rebound to yield the hydroxylated product. In enzymes, the rebound is rapid, while in most synthetic Fe(IV)=O complexes, the rebound reaction competes with trapping of the alkyl radicals by O_2_.^13^ Therefore, reactivity studies are usually performed under anaerobic conditions,^8^ since O_2_ scavenges C-centered radicals (diffusion-controlled rates) to produce alkyl peroxyl radicals, which can react further via the Russell termination mechanism^14^ and other oxidation reactions to produce oxygenated alkyl products. However, the effect of O_2_ on the catalyzed reactions themselves is less well understood.

The relative stability of several of the “activated” species (e.g., Fe(III)-OOH, Fe(IV)=O, etc.) formed from the complex [(N4Py)Fe(II)(CH_3_CN)]^2+^ (**1**), a functional biomimetic model for the antibiotic bleomycin,^15^ has enabled their complete spectroscopic characterization. This Fe(IV)=O species can oxidize several classes of organic compounds.^8,16−20^ The reactivity of [(N4Py)Fe(IV)=O]^2+^ (**4**) is increased by photoexcitation into its near-UV absorption bands to form a short-lived but highly reactive Fe(IV)=O species (4*).^21^ Reaction of **1** with H_2_O_2_ leads to a ferric hydroperoxido intermediate, [(N4Py)Fe(III)-OOH]^2+^, which is also able to engage in catalytic alkane oxidation, albeit with low TON (e.g., 36 turnovers over several hours).^15^ It can be assumed to undergo homolytic cleavage of the O–O bond to generate **4** and a hydroxyl radical, both of which can engage in oxidative processes such as C–H bond hydroxylation. However, recent studies have shown that the primary reaction pathway is catalytic disproportionation of H_2_O_2_ through a mechanism directly involving [(N4Py)Fe(III)–OOH]^2+^^22^ and that homolytic cleavage of the O–O bond in [(N4Py)Fe(III)–OOH]^2+^ to yield [(N4Py)Fe(IV)=O]^2+^ is too slow to be kinetically competent.^23^ Furthermore, the O_2_ produced by disproportionation of H_2_O_2_^24^ may react with organic radicals formed after hydrogen atom abstraction, yielding products due to the Russell mechanism even under anaerobic conditions.^14^ Hence, the involvement of O_2_ and H_2_O_2_ in these reactions must be considered carefully.

Terminal oxidants other than H_2_O_2_ can be used to activate nonheme iron complexes, such as O atom donors ArIO, ROOH, RC(O)OOH, and hypohalites.^25^ In particular, iron(III)–acylperoxo complexes, generated using organic peracids, can form iron(IV)–oxo species through homolytic cleavage of the O–O bond. These species have advantages over the corresponding hydroperoxo counterparts because the O–O bonds of the former cleave more easily due to the additional driving force provided by the ensuing release of CO_2_. In addition, **1** and peracids, compared to H_2_O_2_, show higher catalytic activity in alkane, alcohol, and aromatic oxidation, demonstrated by better selectivity and higher TON achieved.^8^ Furthermore, these oxidants, in contrast to H_2_O_2_, do not typically release O_2_ through disproportionation. The presence of O_2_ during the oxidation of alkanes with peracids has a clear impact on selectivity and catalytic activity, resulting in much lower alcohol/ketone ratios, which are ascribed to a Russell mechanism.^8^ The impact of O_2_ on the speciation of the iron catalyst during the reaction is less obvious but equally important given that its presence also reduces the overall efficiency in substrate oxidation.

The activation of **1** by peracid species was reported earlier.^4,26,27^ The presence of H_2_O_2_ in most commercially available organic peracids complicates the study of their reactivity with iron complexes, however. For example, peracetic acid (PAA), the most widely used peracid, contains a substantial amount of H_2_O_2_, typically 5% by weight, corresponding to ca. 0.3 equiv/PAA equivalent in commercial samples. Such amounts are sufficient to interfere through hydroperoxide activation and mask reactions between the iron complex and the peracid itself (vide infra). *meta*-Chloroperoxybenzoic acid (*m*-CPBA) is available commercially as a solid and usually contains only residual H_2_O_2_. Ray et al. used *m*-CPBA with **1** to study the mechanism of O–O bond lysis through the analysis of peracid decomposition products.^27^ However, as shown by Que et al.,^28^ this oxidant is less suitable due to the formation of interfering byproducts, such as salicylic acid, which can form stable Fe(III) phenolato complexes.

Although it can be prepared in solid form, with little if any H_2_O_2_ present, only a few examples have been reported in which phenylperacetic acid (PhPAA) is used to study the reactivity of nonheme iron complexes.^26^ Interestingly, this reagent can generate high-valent iron species without *in situ* generation of O_2_, interference from H_2_O_2_, or decomposition products due to arene oxidation.^28^ In addition, the decomposition products formed from PhPAA^29^ can be readily characterized by GC, HPLC, and/or ^1^H NMR analysis without workup.

**Scheme 1:**
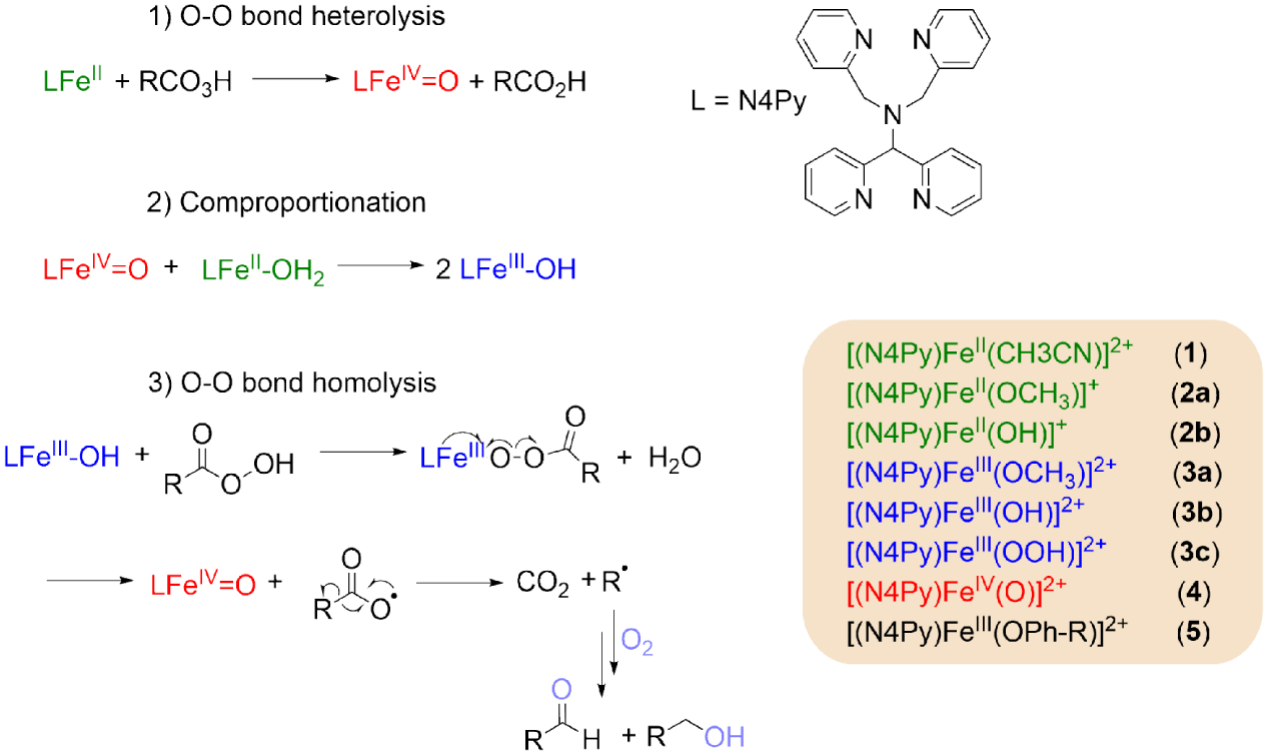
Expected Mechanism for the Activation of **1** By Peracids, and the N4Py Iron-Species Discussed in the Text Note that the protonation state in the case of **2a** and **2b** is uncertain.

Here, we report the reaction of **1** with phenylperacetic acid (PhPAA) in alcohols (methanol and trifluoroethanol) using in-line reaction monitoring with Raman, visible absorption, headspace FTIR absorption, and luminescence spectroscopy. The generation of specific products (both organic compounds and CO_2_) derived from the homolytic cleavage of the O–O bond of an intermediate Fe(III)–OOC(O)R (Scheme 1) is rationalized.^9,25,30^

**Scheme 2:**
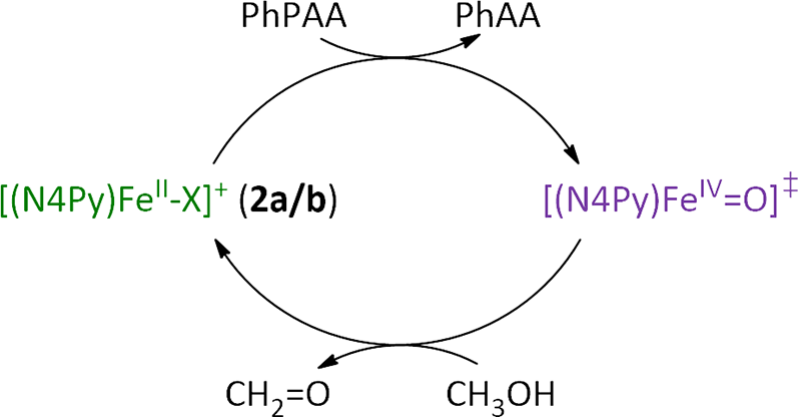
Catalytic Activity of **2a** Toward Methanol Oxidation through a Transient Fe(IV)=O SpeciesX *X* = CH_3_OH, CH_3_O^–^(**2a**), H_2_O, or HO^–^ (**2b**), note that it is possible that *X* = CH_3_OH (**2a**) or H_2_O (**2b**) under reaction conditions, however, coordination of CH_3_O^–^ and OH^–^ is indicated only for simplicity.

Although the product distribution shows only a modest dependence on the concentration of dissolved O_2_, we demonstrate that its role in trapping intermediate organic radicals heavily impacts the relative concentrations of the various iron species observed. Kinetic analysis, supported by multivariate curve resolution (MCR) analysis, reveals these effects and provides strong evidence toward the transient involvement of a more reactive species than the well-characterized **4**, enabling the catalyst to oxidize methanol with 40% efficiency in oxidant (Scheme 2).

## Results and Discussion

**Figure 1:**
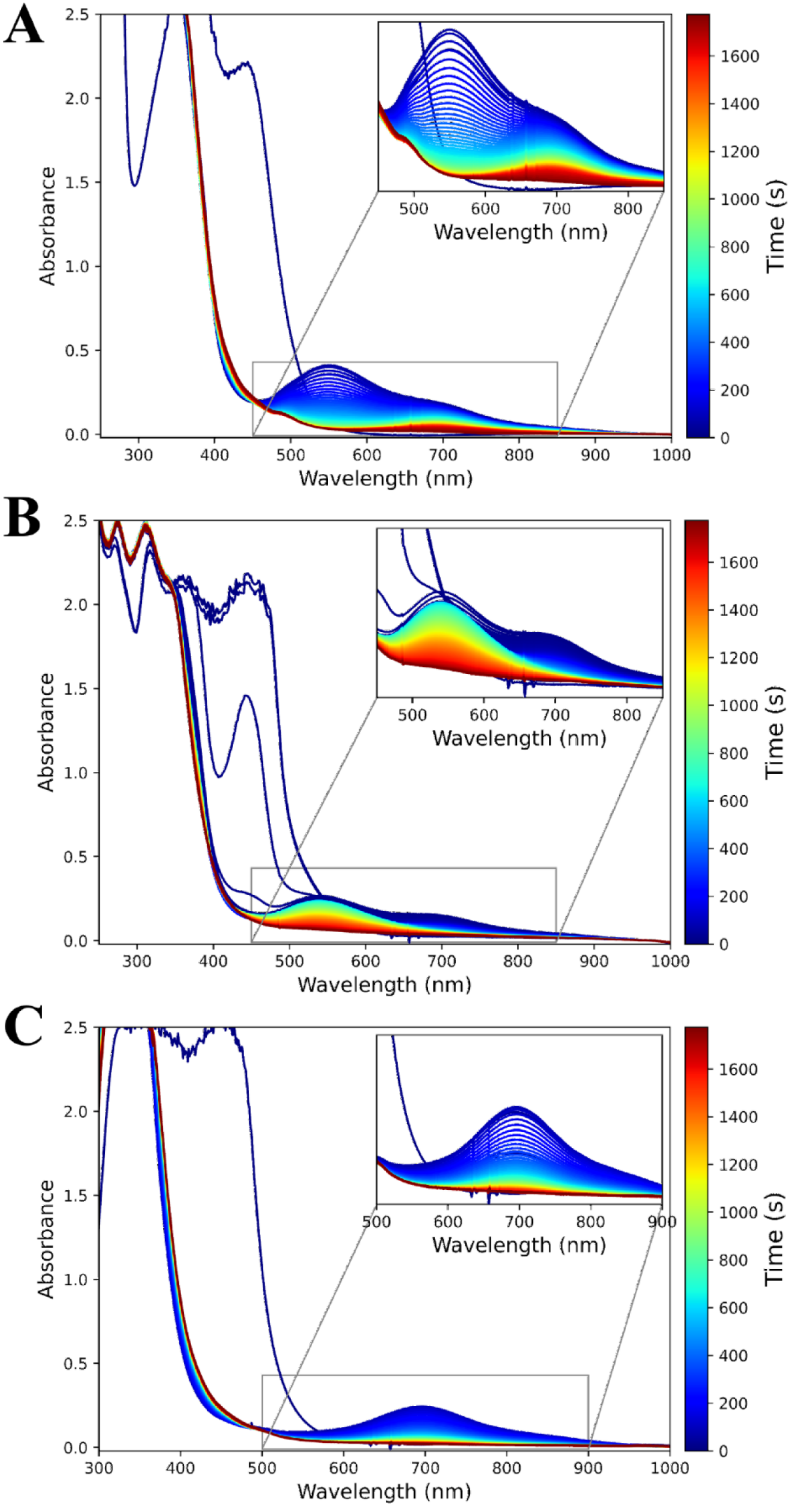
UV/vis absorption spectra over time of **1** (1 mM) in CH_3_OH with (A) 10 equiv of PAA, (note that commercial PAA contains up to 6 wt % H_2_O_2_) (B) 10 equiv of PhPAA + 3.2 equiv of H_2_O_2_, and (C) 10 equiv of PhPAA. Insets show the characteristic bands of the Fe(III)–OOH (at 550 nm, **3c**) and Fe(IV)=O (at 695 nm, **4**) species. See SI for further details and MCR analyses (Figures S2–S8).

The reaction of **1** with organic peracids was studied in methanol and 2,2,2-trifluoroethanol (TFE) because, although the exchange of its CH_3_CN ligand with H_2_O, ROOH, or H_2_O_2_, etc., is thermodynamically unfavorable (ca. 80 kJ/mol based on differences in redox potentials), it is nevertheless rapid in these solvents.^31^ The equilibrium between **1** and complexes [(N4Py)Fe(II)OCH_3_]^+^ (**2a**) or [(N4Py)Fe(II)OH]^+^ (**2b**) etc., is established essentially immediately upon dissolution.^22^ Phenylperacetic acid (PhPAA) was selected as the oxidant since it can be prepared without residual H_2_O_2_. The presence of H_2_O_2_ in a peracid (e.g., in PAA; Figure 1A,B; see also Figures S2–S8 for MCR analysis) complicates studies of the peracids’ reactivity with catalysts. For example, in methanol, the addition of a small excess of peracetic acid (PAA) (10 equiv) results in a rapid decrease in the visible absorption of **2a,** concomitant with the appearance of the characteristic absorption band at ca. 550 nm of the complex [(N4Py)Fe(III)–OOH]^2+^ (**3c**; Figure 1A).^15^ The absorbance at 550 nm eventually decays with the appearance of the characteristic^16^ absorption band of [(N4Py)Fe(IV)=O]^2+^ (**4**) at 695 nm.^32^ Spectra of **1**, **2a,** and **4** are shown in Figure S1 for reference. The initial formation of **3c** indicates that oxidation to the Fe(III) state and formation of the relatively stable Fe(III)-OOH is rapid, impeding further reaction with PAA until the H_2_O_2_ present is disproportionated.^22,23^ Thereafter, the Fe(IV)=O species (**4**) is formed to a significant extent. Two points are of note with regard to the reaction progress observed by UV/vis absorption spectroscopy. First, although **4** can form through O–O bond homolysis in [(N4Py)Fe(III)–OOH]^2+^, this reaction is too slow to be significant on the time scale of the reactions discussed here,^23^ and hence **4** must form by reaction of the iron(III) complexes with peracetic acid. Second, **4** reacts rapidly with stoichiometric H_2_O_2_^21,24^ and hence its appearance is indicative that H_2_O_2_ is no longer present in solution.

In contrast, the addition of 10 equiv of phenylperacetic acid (PhPAA), prepared free of H_2_O_2_, to **1** in methanol results in the rapid appearance of an absorption band at 695 nm with negligible absorbance at 550 nm (Figure 1C).^33^ It is notable that the time dependence of changes in the concentration of the iron species correlates well with the release of CO_2_ into the headspace under conditions of limited oxidant under air (Figure S9 for further discussion and Figures S10 and S11).

### Reaction of **1** with PhPAA Under Air

**Figure 2:**
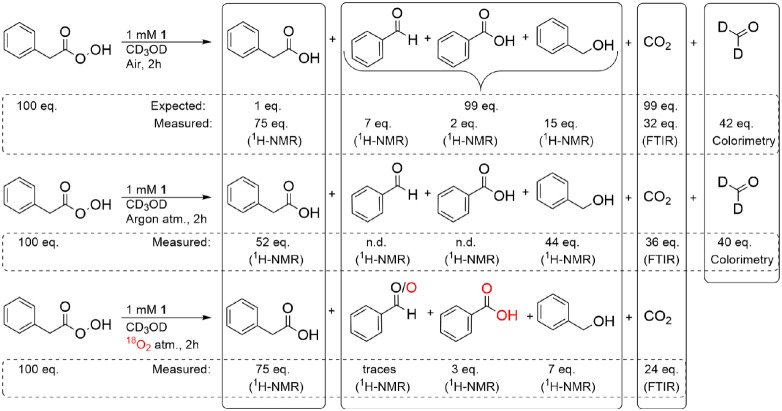
Mass balance for products obtained 2 h after addition of 100 equiv of PhPAA to 1 mM **1** in CD_3_OD under air, an argon atmosphere, and under ^18^O_2_ atmosphere. PhCOOH, PhCHO, PhCH_2_OH, and PhAA were determined by ^1^H NMR spectroscopy, CO_2_ by headspace FT-IR spectroscopy, and CD_2_O by colorimetry.

**Figure 3:**
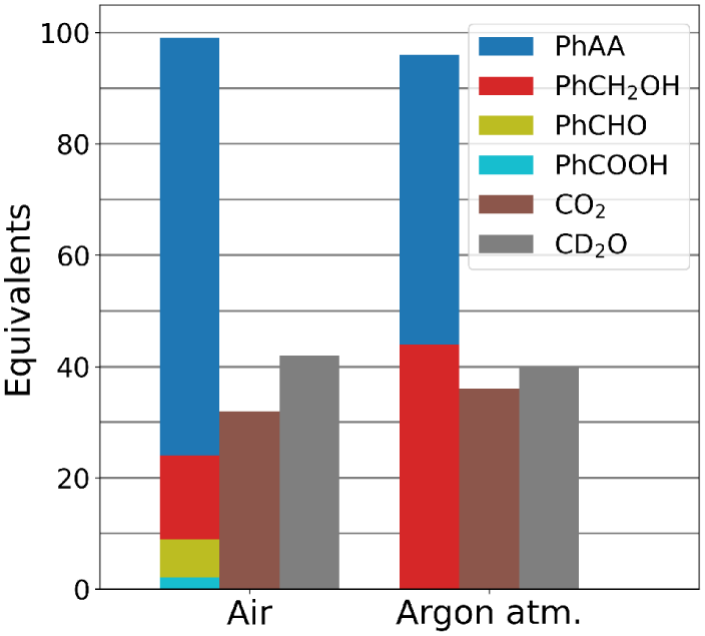
Bar chart showing equivalents of products (with respect to 100 eq of PhPAA) obtained 2 h after addition of 100 equiv of PhPAA to **1** (1 mM) in CD_3_OD under air, and under an argon atmosphere. PhCOOH, PhCHO, PhCH_2_OH, and PhAA were determined by ^1^H NMR spectroscopy, CO_2_ by headspace FT-IR spectroscopy, and CD_2_O by colorimetry. See the SI for details.

**Figure 4:**
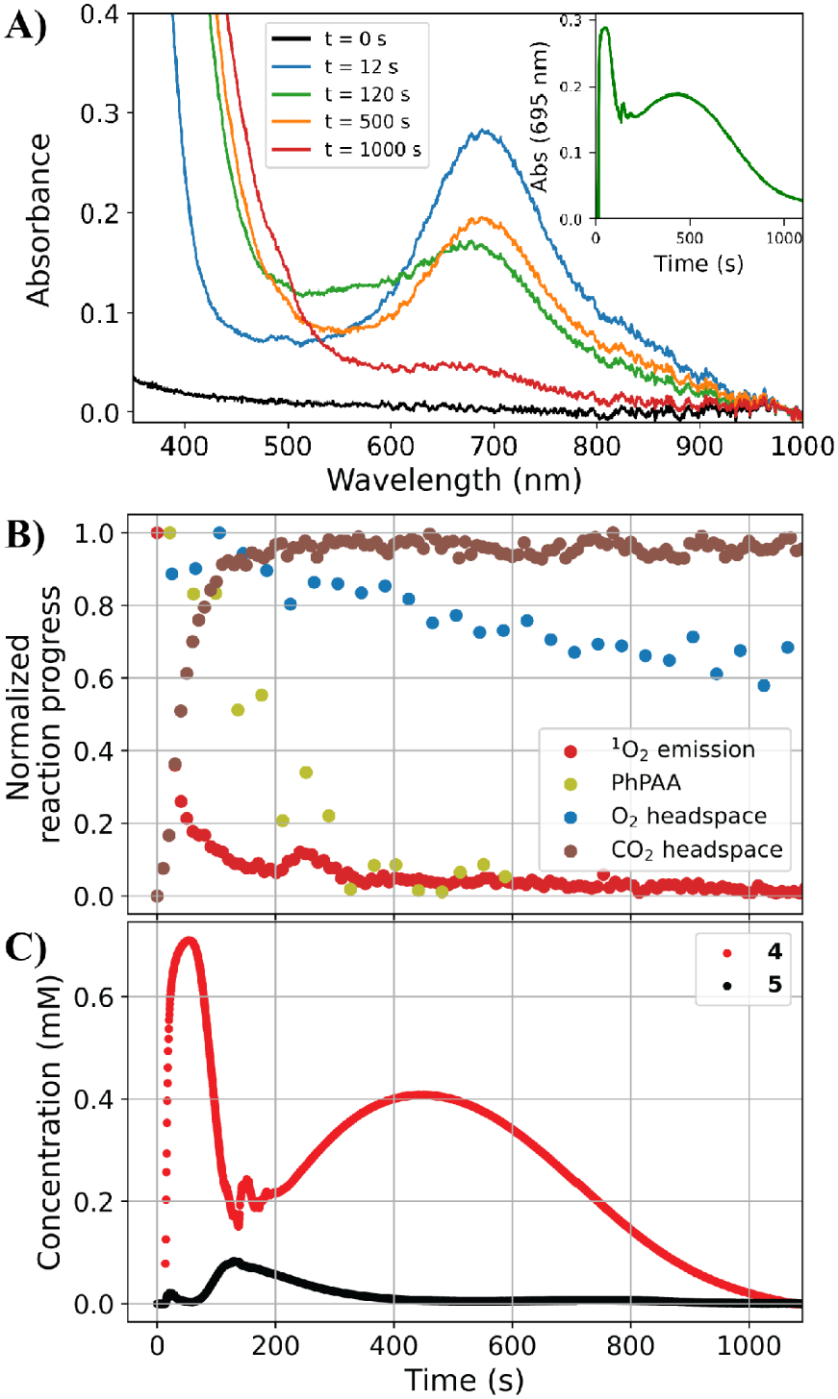
(A) UV/vis absorption spectra of **1** before and over 1000 s after addition of 100 equiv of PhPAA acid (inset shows absorbance at 695 nm over time). Reaction progress of (B) CO_2_ released (brown, determined by FTIR), PhPAA (olive, determined by Raman spectroscopy), O_2_ in headspace (blue, determined by headspace Raman spectroscopy), and O_2_ in the liquid phase (light red, determined by ^1^O_2_ emission by luminescence spectroscopy), and (C) MCR comp. 2, corresponding primarily to **4** (red), and MCR comp. 3, corresponding primarily to **5** (black). Conditions: 1 mM **1**, 100 mM PhPAA, in 1.5 mL of MeOH under air.

The reaction of **1** with a large excess of PhPAA (100 equiv) was monitored spectroscopically and offline to determine product distributions (Figures 2 and 3) and to establish reactivity under catalytically relevant conditions. The NIR absorption band of **4** at 695 nm (ca. 75% of the initial **1**, considering its molar absorptivity) appears within seconds of addition to PhPAA (Figures 4 A and S12A). The absorbance remains constant for ca. 20 s, followed by conversion to other species over ca. 100 s (Figures 4A and S12B). Surprisingly, after ca. 200 s, the absorbance at 695 nm increases again over 100 s and thereafter decreases slightly faster than would be expected for the self-decay of **4**(16,21) to **3b** in methanol (Figure S12C). The more rapid decrease is attributed to the oxidation of formaldehyde (Figure S13) formed unexpectedly during the reaction (Figure 2).

It is noteworthy that the time course, as well as the extent of changes in NIR absorbance, varied from reaction to reaction (Figure S14). Furthermore, the erratic changes in absorbance, for example at ca. 150 s in Figure 4, are not due to spectral artifacts but instead are caused by variations in mixing efficiency (stirring) from reaction to reaction, which affect the rate of mass transport of O_2_ from the headspace to the reaction mixture (vide infra).^34^

Due to the overlap in the absorption spectra of the various species formed during the reaction, multivariate curve resolution (MCR) analysis was employed to extract the time dependencies of their concentrations.^35^ The MCR analysis of the UV/vis absorption spectra yielded three significant components (Figure S12D).^36^ Component 1 (Figure S12D, blue trace) most closely resembles the absorption spectrum of an Fe(III) complex, e.g., **3a,** and component 2 resembles that of **4** (Figure S12D, red trace). A third component (ascribed to an Fe(III)-phenolato complex, **5**; for characterization see Figures S15, S16, and S17) was evident in the visible absorption spectrum, with a maximum at ca. 580 nm (Figure S12D, black trace). The lack of absorbance at ca. 450 nm excludes the recovery of an Fe(II) species, i.e., **2a** or **2b**, to a significant extent. The outcomes of the MCR analysis (i.e., original data vs. fitted data and residuals) are shown in Figures S18 and S19.

The correspondence between the time dependence of the concentrations of the various iron species (specifically MCR components) with the consumption of PhPAA, the release of CO_2_, and the change in O_2_ in the headspace is shown in Figure 4. PhPAA is mostly consumed within ca. 400 s (Figure S20), whereas CO_2_ is released (ca. 32% w.r.t. PhPAA, Figure S21) over the first 150 s and O_2_ is consumed (ca. 30% w.r.t PhPAA, Figure S22) over 1500 s. MCR analysis shows an inversion in the relative concentration of **4** and **5** after ca. 100 s, which reverses again at ca. 250 s (Figure 4). The first transition between the two components is in good agreement with the time over which CO_2_ evolved, reaching its final level at ca. 150 s. Notably, at that time, PhPAA is not yet fully consumed (it is fully depleted only after ca. 400 s) and O_2_ continues to be depleted from the headspace. The reaction mixture ultimately contained 75% phenylacetic acid, 15% benzyl alcohol, 2% benzoic acid, and 7% benzaldehyde with respect to the initial [PhPAA] (by ^1^H NMR spectroscopy, Figure S23). The reaction also produces ca. 42 mM of formaldehyde (determined at the end of the reaction colorimetrically), which is unexpected,^22^ as the rate of reaction of **4** with methanol is too slow to achieve this over the time scale of the reaction.

The reaction products derived from the benzyl radical, the evolution of CO_2_, and the loss of O_2_ from the headspace are consistent with the trapping of radicals formed after homolysis of the O–O bond of PhPAA. However, the depletion of dissolved O_2_ also correlates with the ratio of the various iron species formed, which prompted us to determine the concentration of dissolved O_2_ in situ spectroscopically (see SI and Figures S24–S28 for details).^23^

Addition of excess PhPAA to **1** results in a decrease in the concentration and eventually the complete consumption of dissolved O_2_ (Figures S29 and S30). During this time, the NIR absorption band of **4** initially increases, decreases again, and eventually recovers. Hence, the depletion followed by the recovery of **4** is correlated with the concentration of dissolved O_2_; i.e., in the presence of PhPAA, reactions involving O_2_ serve to increase the steady-state concentration of **4**.

The change in UV/vis absorption upon the addition of PhPAA to **1** in trifluoroethanol (TFE) shows similar progress over time as in methanol, with a longer overall reaction time and only minor differences in the absorbance of the various iron species, consistent with the higher solubility of O_2_ in TFE^37,38^ and the lower rate of self-decay of **4** in this solvent (Figure S31). MCR analysis provides the same three components, which correspond well to the absorption spectra of the expected iron species (Fe(III), **4**, and **5**; Scheme 1 and Figures S32–S35). The contribution of these components over time was similar to that observed in methanol.

The data indicate that, in addition to the expected participation of O_2_ in benzyl radical trapping, O_2_ also influences the time dependence of the appearance and disappearance of iron species. However, the reasons why O_2_ interferes with the reactions of the various iron complexes and PhPAA remain unclear. Since the mass transport of O_2_ between air and methanol is dependent on mixing efficiency and its concentration varies significantly over the course of these reactions, further experiments were carried out under limiting conditions of O_2_ availability—namely, under argon to exclude O_2_ completely or with 1 atm of ^18^O_2_ (for atom tracking as well) with a large headspace to maintain the concentration of dissolved O_2_ in solution, both in CH_3_OH and CD_3_OD.

### Reactions Under Argon and Under ^18^O_2_ in CH_3_OH

**Figure 5:**
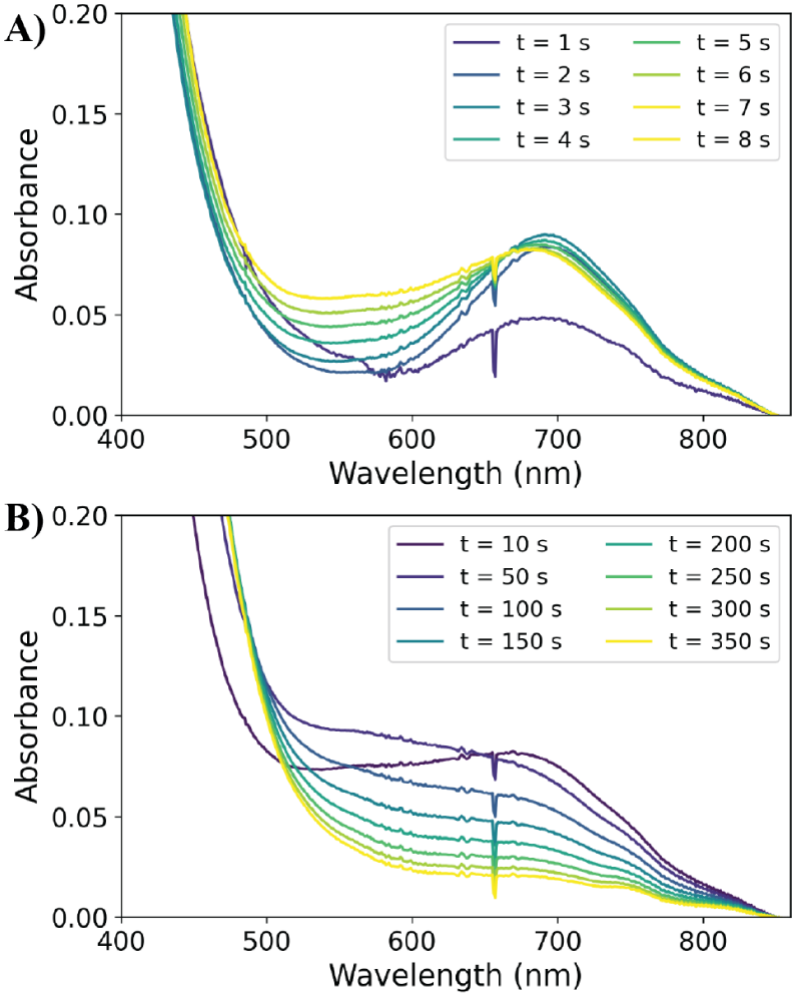
UV/vis absorption of **1** with 100 eq. PhPAA in methanol under argon over (A) 8 s and (B) 350 s. The characteristic absorption bands of **4** (at 695 nm) and **5** (at 580 nm) are evident. Conditions: 1 mM **1**, 100 mM phenylperacetic acid in 1.5 mL of MeOH under argon.

Under argon, a rapid initial change in UV/vis absorption is observed, followed by a subsequent slower change in vis-NIR absorption (Figures 5, S36, and S37). The absorption bands observed are consistent with the expected species: **4**, **5**, Fe(III) (**3a**/**3b**), and Fe(II) (**2a**/**2b**). Over the first 50 s (Figure 5), the formation of **4** and the subsequent formation of **5** are evident (Figure S36). The absorbance of **5** then decays over a longer period.

The relative, as well as absolute, concentrations of the various iron species over time are different from those observed under air (Figures S36C,D and 6). Specifically, the NIR absorbance of **4** increases rapidly after the addition of PhPAA, as under air, but then decreases again—initially quickly and then more slowly. Its concentration is, at all times, much lower than that observed under air. Notably, the absorbance of **5** increases and decreases at a rate similar to that observed under air, but begins much earlier. Furthermore, in contrast to the conditions under air, the NIR absorbance of **4** does not recover later in the reaction.

**Figure 6:**
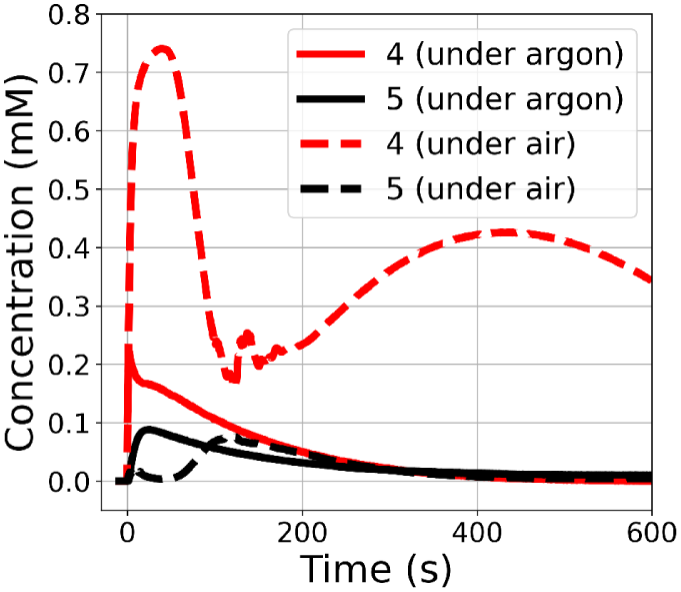
Normalized reaction progress from MCR analysis showing component concentrations corresponding to **5** (black) and **4** (red) under air (dashed lines) and argon (solid lines). Concentrations of **5** assumes a molar absorptivity of 1000 M^–1^·cm^–1^ at 580 nm.

**Figure 7:**
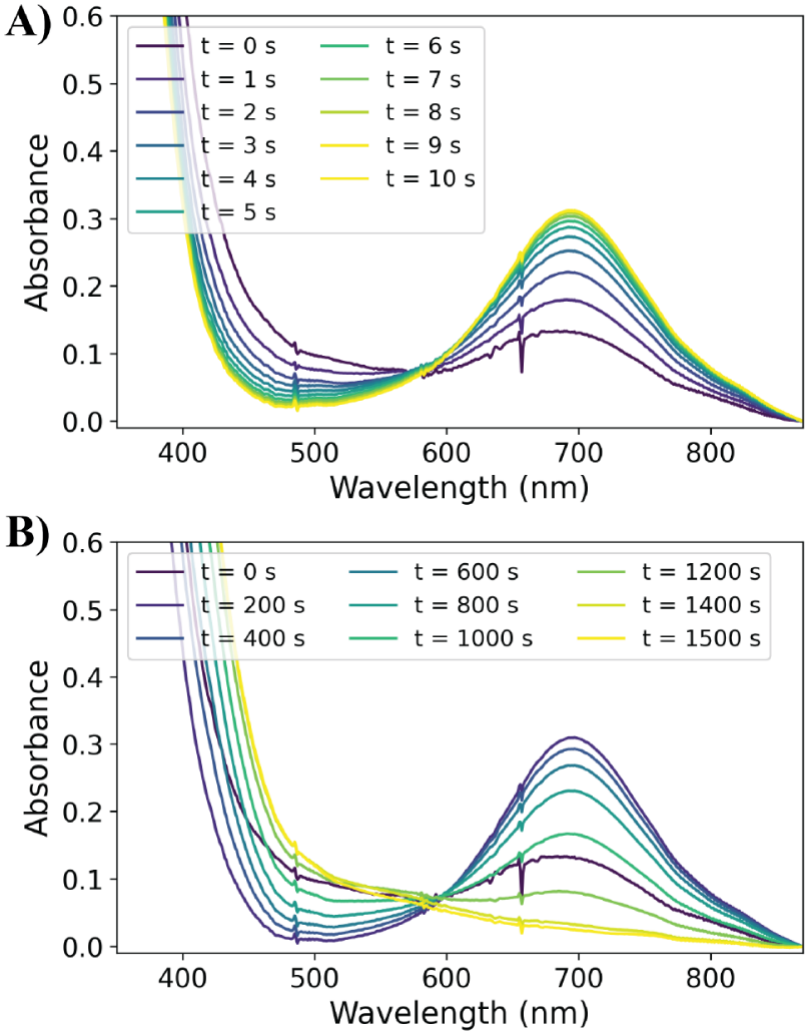
UV/vis absorption spectra of **1** with 100 equiv. PhPAA in methanol under ^18^O_2_ atmosphere between (A) 0 and 10 s and (B) 10 and 1500 s. Conditions: 1 mM **1**, 100 mM phenylperacetic acid in 1.5 mL of MeOH under ^18^O_2_.

In contrast, in ^18^O_2_ purged solutions (with 1 atm of ^18^O_2_ in the headspace), **4** is formed to a greater extent and persists at this higher steady-state concentration for much longer, with a relatively constant absorbance of ca. 0.32, before decaying finally at a slightly faster rate than observed under air due to the presence of formaldehyde (vide supra, Figures 7, S38, and S39).

### Reactions Under Argon and Under ^18^O_2_ in CD_3_OD

Analogous experiments were carried out under argon and under ^18^O_2_, in CD_3_OD to limit contributions from the reaction of **4** with the solvent.^21^ These limiting situations, in regard to O_2_ availability, allow for products formed through the reaction of **1** and PhPAA, and products due to the reaction of organic radicals formed with molecular oxygen, to be distinguished. The reactions under argon and ^18^O_2_ were followed by UV/vis absorption, Raman (headspace), and FTIR spectroscopy simultaneously, followed by direct analysis of the reaction mixture by ^1^H NMR spectroscopy and GC-MS. The extent of formaldehyde formation was determined for the reaction carried out under argon.

Under argon, MCR analysis of the UV/vis absorption spectra (see SI for details of MCR analysis, Figures S40–S43) reveals components and time dependence similar to the reaction in CH_3_OH (Figure S44), with the only difference being that, in CD_3_OD, the decay of Fe(IV)=O and the appearance and decay of **5** are less rapid. Headspace analysis shows that CO_2_ is released (ca. 36% w.r.t. PhPAA, Figures S45 and S46), and by ^1^H NMR spectroscopy, 52% of the PhPAA was converted to phenylacetic acid and 44% to benzyl alcohol (Figure S47). Formaldehyde-d_2_ was also produced (ca. 40 mM by the end of the reaction). In addition, formaldehyde formation was not impacted by solvent deuteration (ca. 37 mM in MeOH).^39^ The yields of each product are summarized in Figure 2.

**Figure 8:**
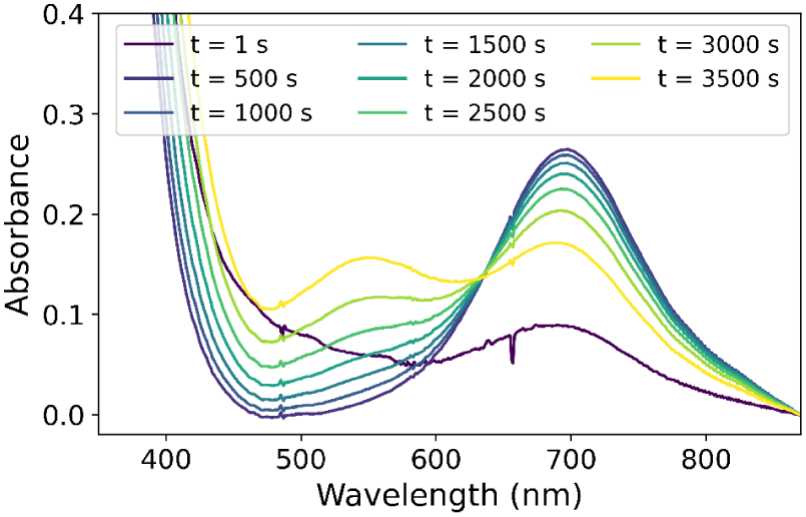
Reaction of **1** with PhPAA in CD_3_OD under ^18^O_2_ atmosphere monitored by UV/vis absorption spectroscopy: (A) from 0 to 3500 s and (B) from 3500 to 5370 s. Conditions: 1 mM **1**, 100 mM phenylperacetic acid in 1.5 mL of CD_3_OD under ^18^O_2_.

Under ^18^O_2_ in CD_3_OD, the overall changes in the UV/vis absorption spectra were again similar to those observed in CH_3_OH (Figures 8 and S48–S50), and again the rate of decay of **4** is lower in CD_3_OD.^21^ Furthermore, the maximum absorbance of **5** reached is higher, which indicates that the oxidation of methanol competes with the aromatic hydroxylation of phenylacetate (vide infra).

Release of CO_2_ (24% w.r.t. PhPAA), and depletion of ^18^O_2_ from the headspace, respectively, are also observed (Figures S51 and S52). The ratio and amount of PhPAA degradation products are similar to those observed under air (Figure S53). GC-MS analysis shows the presence of ^18^O-labeled benzaldehyde and benzoic acid (Figure S54), indicating that O_2_ is involved in their formation. However, the benzyl alcohol formed does not contain ^18^O, indicating that this product is formed without the direct involvement of O_2_, i.e., not via the Russell mechanism.^40^

### Mechanistic Considerations and Impact of O_2_ on Catalyst Speciation

**Scheme 3:**
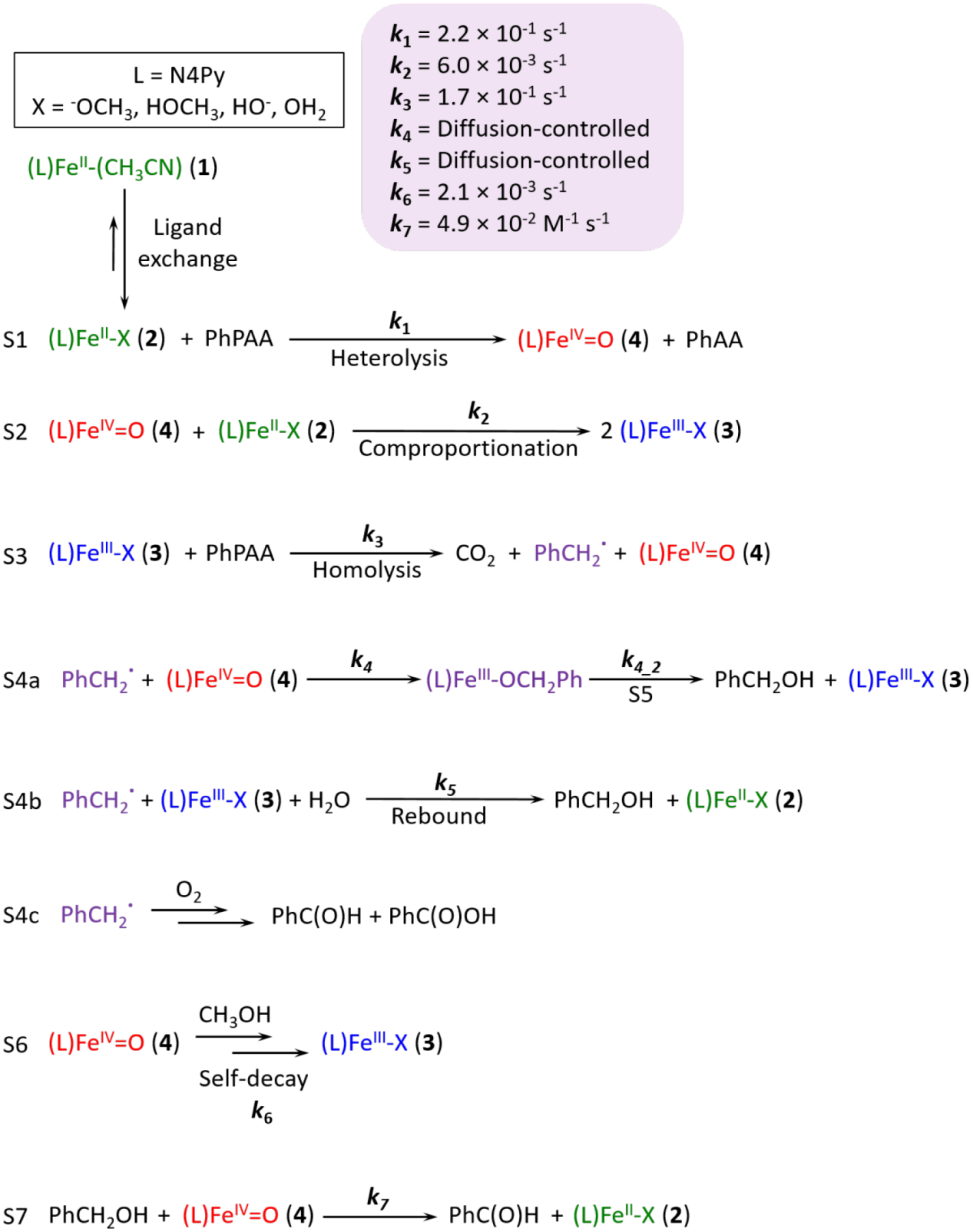
Reactions Following Addition of Excess PhPAA to **1** in Methanol Including Available Observed Rate Constant, *k*_obs_, for Elementary Steps *k*_7_ is taken from Morimoto et al.^100^ Note that, under argon, reaction S4c does not occur due to the absence of O_2_ and reaction S7 is not observed due to the absence of significant concentrations of **4.**

The relative amounts of each of the iron species formed over time with PhPAA are sensitive to reaction conditions, especially the presence or absence of O_2_. The reactions (Scheme 3, S1–S7) that are expected to take place following the addition of PhPAA to **1** are summarized in Scheme 3. The Fe(II)–peracid complex formed initially upon the addition of PhPAA undergoes heterolytic O–O bond cleavage (Scheme 3, S1) to form **4** and phenylacetic acid (1 equiv). Although comproportionation of residual **2b** and **4** to produce **3b** should be considered (S2, *k*_2_ = 6.0 × 10^–3^ s^–1^),^21^ the rate at which PhPAA reacts with **2a** is also high (*k*_1_ = 2.2 × 10^–1^ s^–1^) and results in the conversion of at least 75% of **2a** to **4** in the first seconds of the reaction, with the remainder (25%) converted to **3a**.

Addition of 100 equiv of PhPAA to preprepared **4** shows only the conversion of residual **3a** present to **4** (Figure S55, *k*_obs_ = 2.2 × 10^–1^ s^–1^ for the formation of the Fe(IV) state). This can be expected since, although **4** reacts rapidly with H_2_O_2_,^22^ the H–O BDE of PhPAA is greater than for H_2_O_2_ (by more than 10 kcal/mol, Tables S1 and S2). Furthermore, the later addition of 100 equiv of PhPAA to the reaction mixture, when all iron has converted to the Fe(III) state, shows rapid (within seconds) conversion to **4** (Figure S56, *k*_obs_ = 1.7 × 10^–1^ s^–1^ for the formation of **4**). **3a** reacts with PhPAA to form [(N4Py)Fe(III)–O–OC(O)CH_2_Ph]^2+^ transiently, followed by homolysis of the O–O bond to form **4** and PhCH_2_CO_2_^·^. In both cases, the subsequent reaction progress is the same as observed with **1** (*k*_1_ in Scheme 3). Hence, we can conclude that **4** does not react with PhPAA directly.

Homolysis of the O–O bond in [(N4Py)Fe(III)-O-OC(O)CH_2_Ph]^2+^ is driven by subsequent spontaneous decarboxylation to form CO_2_ and PhCH_2_^•^. The reaction of PhCH_2_^•^ with O_2_ generates PhCH_2_OO^•^, which decomposes to form benzaldehyde exclusively, e.g., via a non-Russell mechanism pathway. The product can be oxidized further by **4** to benzoic acid, etc.,^21^ regenerating an Fe(III) complex (eventually **3a**). However, PhCH_2_^•^ can also react with **4** to generate an Fe(III) complex ([(N4Py)Fe(III)–O–CH_2_Ph]^2+^) in a manner analogous to the rebound reaction between PhCH_2_^•^ and **3b** to generate [(N4Py)Fe(II)–O(H)–CH_2_Ph]^2+^.^41^

Hence, the expected outcome of the reaction is ca. 1 equiv of phenylacetic acid, with the remaining PhPAA converted to CO_2_, benzaldehyde, benzoic acid, etc. (Figure 2). Experimentally, however, this outcome is not obtained. Instead, phenylacetic acid is formed as the major product, while products from the homolytic pathway are formed in yields of only ca. 10–25% yield.^42^ Furthermore, the amount of formaldehyde (ca. 40% turnover with respect to **1**) is unexpected, as **4** reacts with CD_3_OD too slowly to achieve this turnover number. Hence, an alternative pathway for PhPAA heterolysis, which also leads to methanol oxidation, must be operating.

Reactions carried out under argon and with saturated ^18^O_2_ provide useful insight into the relative importance of the various processes. Under ^18^O_2_ the reaction outcome is similar to that with an air equilibrated solution, and ^18^O labeling is consistent with the aldehyde and benzoic acid forming from the reaction of ^18^O_2_ with a benzyl radical. Notably, the benzyl alcohol obtained does not contain oxygen from O_2_ and is therefore formed via a different pathway than by a Russell termination mechanism. Under argon, there is a significant decrease in the amount of phenylacetic acid formed (52%) and an increase in the amount of benzyl alcohol (44%) and CO_2_, close to a 1:1 ratio. The formation of benzyl alcohol under an anaerobic atmosphere is consistent with its O atom not originating from O_2_, and it is likely that it is formed via rebound with iron(III) species present, such as **3b**.^41^

#### Role of O_2_ in Extent of Formation of **4**

Since the self-decay of **4** in methanol and, especially in CD_3_OD, is slow and **4** does not react with PhPAA, the rapid disappearance of the initially formed **4** under argon is unexpected. Furthermore, the incomplete conversion to **4** under any condition (from 85% under O_2_ to 21% under argon) is surprising, given the rate at which Fe(III) species (**3a**/**3b**) react with PhPAA.

Reaction monitoring shows that, under air or O_2_, PhPAA is consumed prior to the recovery of **4,** indicating that it is the combination of PhPAA and O_2_ that determines the steady-state concentration of Fe(IV)=O, with a clear dependence of the concentration of **4** on the concentration of dissolved oxygen (i.e., **4** is formed when O_2_ is present in solution). Accordingly, in air-equilibrated solvent, in the absence of a headspace to replenish O_2_, the NIR absorbance of **4** does not recover at later times due to all oxygen being consumed and not replaced. These observations together indicate that there is a pathway toward the reduction of **4** that is, in some way, prevented by O_2_.

The Fe(III) species, i.e., **3a** and/or **3b**, formed in the reaction mixture can react with PhPAA, yielding CO_2_, **4**, and the benzyl radical (PhCH_2_^•^, Scheme 3, S3). The benzyl radical can engage in any of three reactions: (i) reaction with O_2_ (S4c), (ii) rebound with **3b** to form [(N4Py)Fe(II)(HO–CH_2_Ph)]^2+^ (S4b),^41^ or (iii) reaction with **4** within the solvent cage to yield [(N4Py)Fe(III)–O–CH_2_Ph]^2+^ (S4a). Reaction with O_2_, **3b,** or **4** likely proceeds at diffusion-limited rates, and hence, the relative contributions should depend on the concentration of the reactants and their relative diffusion coefficients. In addition, the benzyl radical can react with the molecule of **4** formed following the O–O bond homolysis if it does not diffuse away, increasing the contribution of the latter reaction.

**Figure 9:**
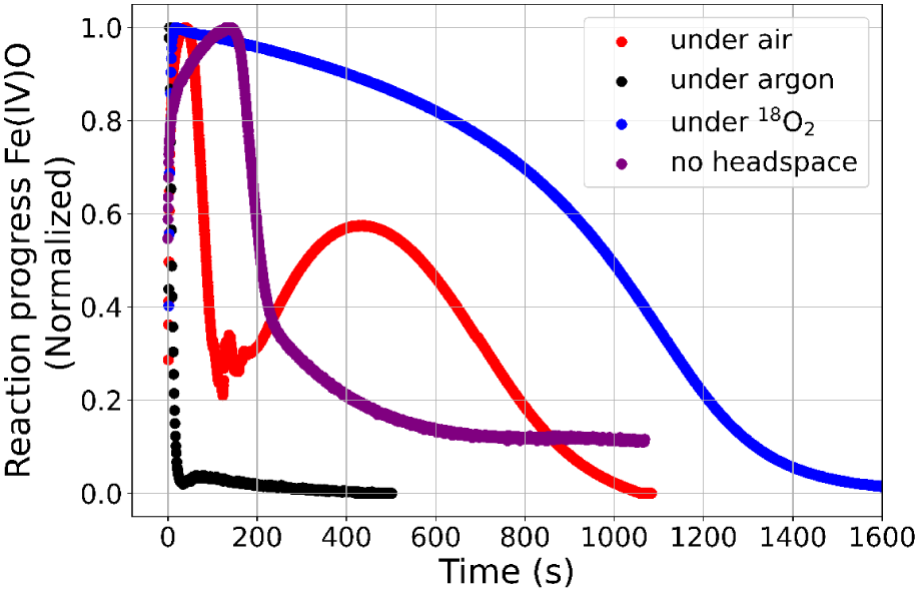
NIR-absorbance of **4** under argon (black), air (red), air equilibrated solution without headspace (purple), and under ^18^O_2_ (blue). Note that traces are normalized to the maximum absorbance in each case: 1 mM **1**, 100 mM phenylperacetic acid in 1.5 mL of MeOH.

The reaction between the benzyl radical and O_2_ is well-known,^43,44^ and its occurrence here is substantiated by the incorporation of ^18^O into benzaldehyde and benzoic acid from ^18^O_2_ and by the absence of these products in the absence of O_2_. It is probable that, in the presence of O_2_, this reaction dominates, and **4** accumulates as a result. Figure 9 shows the NIR absorbance of **4** over time for the reactions under argon, air-equilibrated conditions, air-equilibrated conditions without headspace, and under ^18^O_2_. **4** forms rapidly at the beginning of the reaction in all cases; its concentration decreases rapidly under argon and accumulates under air only as far as dissolved O_2_ is present (<200 s). When dissolved oxygen is mostly consumed (ca. 200 s), the absorbance of **4** decreases. The recovery of **4** occurs only after 200 s, when the rate of replenishment of O_2_ from the headspace becomes competitive with the consumption of O_2_ in the reaction mixture. Accordingly, in a reaction carried out in an air-equilibrated solution but without headspace (a fully filled cuvette so that there is no additional influx of O_2_), **4** does not recover. Only when O_2_ is depleted from the solution do the other two reactions (Scheme 3 S4b and S4a) become competitive, consuming **4**.

Under ^18^O_2_ (with a headspace), the concentration of dissolved O_2_ remains sufficiently high in solution to result in the accumulation of **4** and its persistence. After ca. 800 s, **4** decays with a rate constant slightly greater than the self-decay in methanol due to its reaction with the formaldehyde formed, which is in agreement with the rate of oxidation of 10 equiv of formaldehyde by independently prepared Fe(IV)=O (Figure S13).

The occurrence of reaction S4a in Scheme 3 is indicated by several observations. It is certain, for two reasons, that the benzyl alcohol is not formed by the reaction of the benzyl radical with O_2_. First, in the absence of O_2_ (i.e., by argon gas purge), the proportion of benzyl alcohol obtained with respect to phenylacetic acid increases. Second, the benzyl alcohol formed in the presence of ^18^O_2_ does not contain ^18^O. Furthermore, **4** accumulates only when O_2_ is present in the solution.

The homolysis of the O–O bond upon reaction of PhPAA with the Fe(III) complexes present (i.e., **3a** and **3b**) results in the formation of equal amounts of **4** and a benzyl radical that can combine to form [(N4Py)Fe(III)-OCH_2_Ph]^2+^. The benzyl alcohol can be readily released by exchange with methanol (Scheme 3, S5).^45^**3b** can also react with the benzyl radical to form [(N4Py)Fe(II)OHCH_2_Ph]^2+^ (S4b), which is essentially a rebound reaction;^13^ and, after ligand exchange, the Fe(II) complex reacts again with PhPAA to form **4** and phenylacetic acid via heterolytic O–O bond cleavage.

In addition, under air or ^18^O_2_, when **4** accumulates, oxidation of benzyl alcohol to benzaldehyde via another HAT/rebound process, reforming the Fe(II) complex, is kinetically possible (Scheme 3, S7). Indeed, GC-MS analysis shows that the oxygen atoms in benzaldehyde are only partially derived from ^18^O_2_; hence benzaldehyde is formed via two pathways, one involving ^18^O_2_ (S4c) and one involving the reaction of **4** with benzyl alcohol^21^ (S7). Under argon, **4** can react with the benzyl radical either as it forms or, if they initially escape each other, they can react upon subsequent collisions. Hence, the reaction between **4** and benzyl alcohol occurs to a much lesser extent. It is further noteworthy that the amount of CO_2_ formed in each of the reactions corresponds well with the amount of benzyl-derived products obtained (Figure 3).

These two reactions (Scheme 3, S4a, S4b) continue to occur as long as PhPAA is present in the solution. Once PhPAA is consumed, after ca. 500 s, the **4** species can react only with the solvent (self-decay, S6) and the formaldehyde and benzyl alcohol present (Figure S13).

### Kinetic Modeling and Methanol Oxidation

Although the iron species and reaction products (i.e., phenylacetic acid, benzaldehyde, benzyl alcohol) that are observed are expected, the reaction progress and mass balances, especially for the formation of formaldehyde, are inconsistent with a simple mechanism in which an initial single turnover from Fe(II) to Fe(IV)=O, by oxidation with PhPAA, is followed by the cycling between Fe(III) and Fe(IV)=O states. Furthermore, although steps S4b and S7 in Scheme 3 lead to the reformation of the Fe(II) state, these do not account for the amounts of phenylacetic acid (which should derive from O–O heterolysis), as well as benzaldehyde and benzyl alcohol observed.

**Scheme 4:**
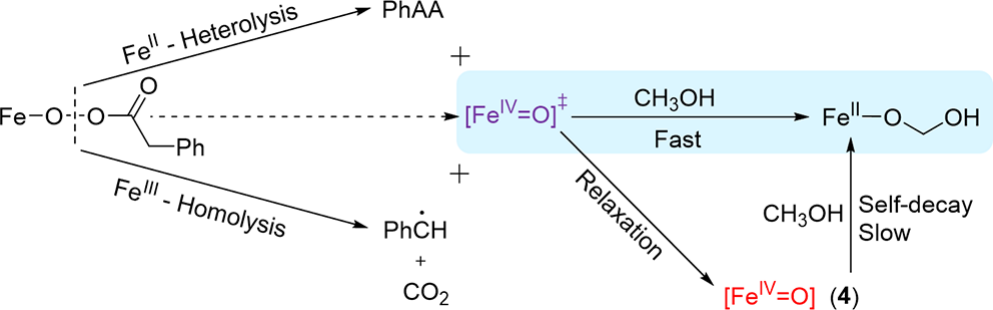
Initial Formation of the More Reactive [Fe(IV)=O]^‡^ Upon Reaction of **2a/b** (Heterolytic Path) and/or **3a/b** (Homolytic Path) with PhPAA The species formed initially on O–O bond cleavage either reacts directly with methanol or relaxes to the less reactive **4.**

The stoichiometric oxidation of methanol to formaldehyde by **4** was shown earlier to be accelerated by photoexcitation; i.e., photoexcitation produced a more reactive species, 4*.^21^ In this study, it should be noted that reaction monitoring was carried out under conditions where photoexcitation does not occur. However, the amount of formaldehyde formed during the reaction of **1** with PhPAA is much greater than can be accounted for by the reaction of **4** with methanol, as this reaction does not occur significantly on the time scale of the experiments reported here. Hence, the formaldehyde must be formed by a more reactive high-valent species than **4**, tentatively assigned as a [Fe(IV)=O]^‡^ species that is formed transiently following homolytic cleavage of the O–O bond in [(N4Py)Fe(III)–O–OC(O)CH_2_Ph]^2+^ and/or following O–O bond heterolysis in [(N4Py)Fe(II)–O–OC(O)CH_2_Ph]^+^ (Scheme 4).

**Figure 10:**
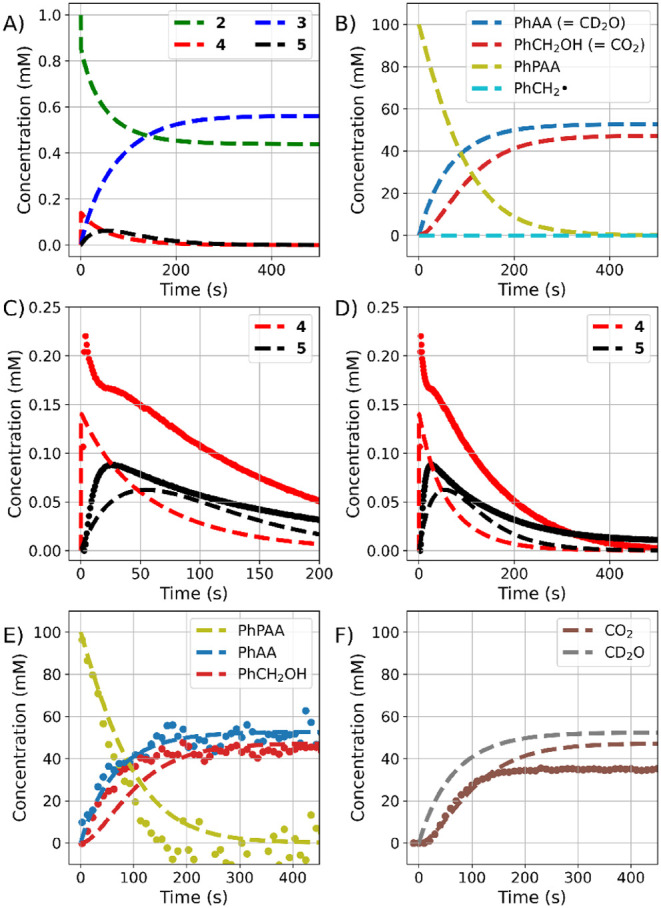
Result of the microkinetic modeling of the reaction of 1 mM **1** with 100 eq. PhPAA under argon with an additional elementary step: the reaction of [Fe(IV)=O]^‡^ and methanol to Fe(II) and formaldehyde. (A) Modeled concentrations of the iron species vs time. (B) Modeled concentrations of phenylperacetic acid (PhPAA), phenylacetic acid (PhAA), CO_2_, benzyl radical, and benzyl alcohol vs time. (C,D) Experimental data from UV/vis and modeled concentrations of **4** and **5** over 200 and 500 s, respectively. (E) Experimental data from liquid phase Raman spectroscopy and modeled concentrations of PhPAA, PhAA, and benzyl alcohol vs time. (F) Experimental data from headspace FTIR spectroscopy and modeled concentrations of CO_2_ and CD_2_O vs time. The dashed and scatter plots represent the model fit and experimental data, respectively. Color legend: **2** (green), **4** (red), **3** (blue), **5** (black), PhPAA (olive), PhAA (light blue), CO_2_ (brown), benzyl radical (cyan), benzyl alcohol (light red), and CD_2_O (gray).

A kinetic model containing the elementary steps described in Scheme 3 for the reaction of **1** with a large excess of PhPAA under argon was constructed, taking into consideration the kinetic competence of **4** to rationalize the product distribution obtained. Reactions carried out under argon were used to construct the model for simplicity, since under air the concentration of O_2_ changes over time due to reaction with benzyl radicals and hence impacts the iron redox cycles. The additional elementary steps involving the reaction of [Fe(IV)=O]^‡^ with methanol in the first solvation sphere to yield Fe(II) and formaldehyde, or relaxation to give **4,** were added to the kinetic model. The resulting model was overall in much better agreement with the experimental data (Figure 10) in comparison to the model without the additional elementary steps (see SI and Figure S57 for further discussion).

Despite the fact that the agreement between the predicted and experimentally observed concentrations is not exact, the predicted concentrations of **4** and **5** are in good agreement with the UV/vis spectroscopic data (Figure 10A, C, D). The predicted concentration of **4** of ca. 0.15 mM, is close to the experimentally determined concentration of 0.2 mM (Figure 10C, D). The modeled concentrations of PhPAA, PhAA, and benzyl alcohol show better agreement with experimental data from Raman and ^1^H NMR spectroscopy (Figure 10B, E) w.r.t. the model that does not consider [Fe(IV)=O]^‡^. The experimentally determined concentrations of CO_2_ and formaldehyde (ca. 40 mM) are also in good agreement (Figure 10B, F). Hence, cleavage of the O–O bond of PhPAA yields a transient [Fe(IV)=O]^‡^ species that is a more potent oxidant than the thermalized complex **4** and accounts for the higher-than-expected reaction rates observed.

## Conclusion

In the present contribution, we employed a combination of in-line spectroscopies (FTIR, UV/vis absorption, Raman, etc.), together with product analysis and kinetic modeling, to establish the reactions that occur in methanol and TFE between **1** and PhPAA, as well as the impact of O_2_ on those reactions.

Under air and O_2_ atmospheres, we observe the formation of **4**; however, the extent of oxidation of methanol to formaldehyde is well beyond its kinetic competence. Furthermore, in the absence of O_2_, the concentration of **4** decreased dramatically. Kinetic modeling of the data reveals the involvement of an intermediate ([Fe(IV)=O]^‡^), which is a more powerful oxidant than **4**, in the reaction of **1** with phenylperacetic acid. While the structure of [Fe(IV)=O]^‡^ is unknown, it is likely that it has a different electronic structure compared to that of **4**, e.g., Fe(III)–O^•^, etc., that relaxes close to the vibrational time scale. The short-lived nature of this intermediate, due to its rapid relaxation to form **4**, means that it can only react with molecules within the first solvation sphere, primarily methanol, phenylacetic acid (when formed from an Fe(II)–OOC(O)CH_2_Ph) and the benzyl radical (when formed from an Fe(III)–OOC(O)CH_2_Ph). The yield of formaldehyde does not allow us to be certain whether [Fe(IV)=O]^‡^ forms during heterolysis or homolysis of the O–O bond of an Fe(II) or Fe(III) peracid complex, respectively, or in both reactions.^46^

The impact of O_2_ on the reaction was revealed to be due primarily to the trapping of the benzyl radical formed upon O–O bond homolysis (in Fe(III)–OOC(O)CH_2_Ph) thereby reducing the opportunity for the reaction of PhCH_2_^•^ with **4** and resulting in the buildup of **4**. The slow reduction of **4** by self-decay means that the overall rate of decomposition of the peracid is reduced, albeit only by a factor of 2. Hence, the main impact of O_2_ is to affect the iron speciation over the course of the reaction, but with eventually only a modest impact on the actual reaction outcome in terms of the distribution of products of PhPAA decomposition.

Overall, it can be concluded that the impact of O_2_ on the oxidation of organic substrates is primarily to reduce the overall reaction rates. In the case of H_2_O_2_ or peracids containing H_2_O_2_, the presence of O_2_ is largely unavoidable, which, together with the formation of **3c**, means that the species observed and their extent of formation over time may not be representative of the actual species that are engaging in the oxidation of substrates. In a broader context, the evidence regarding the transient formation of more reactive iron species than **4** is of importance in catalyst design, where second coordination sphere interactions with substrates to position them close to the reactive center could be advantageous in reacting with the initially formed state, i.e., [Fe(IV)=O]^‡^.

## Methods

### Materials

[(N4Py)Fe(II)(CH_3_CN)](ClO_4_)_2_ and [(N4Py)Fe(IV)=O](PF_6_)_2_ were available from earlier studies.^22^ PhPAA was prepared using the literature method^47^ with 90% active oxygen present. Commercially available chemicals were used as received, without further purification unless stated otherwise. Solvents for spectroscopic measurements were of UVASOL (Merck) grade or better.

### Caution!

Pure peracids (especially as crystalline compounds) may explode under the influence of heat, shock, or spark etc. The drying or concentration of solutions that potentially contain H_2_O_2_ should be avoided. Prior to drying or concentrating, the presence of H_2_O_2_ should be tested using peroxide test strips, followed by neutralization with solid NaHSO_3_ or another suitable reducing agent. All reactions or handling involving peracids or H_2_O_2_ should be carried out behind a blast shield as a precaution, using open vessels and while wearing appropriate safety equipment. Generally, all manipulations should be performed only by individuals suitably trained in handling potentially explosive compounds.

### Physical Methods

All analyses were conducted at room temperature unless stated otherwise. UV/vis absorption spectra were recorded using an Agilent HP8453 spectrophotometer or an Avantes spectrometer and light source in 1 cm path length quartz cuvettes. ^1^H NMR spectra (400 MHz) were recorded on a Bruker spectrometer. Chemical shifts are denoted relative to the residual solvent signals (^1^H NMR spectra CD_3_OD, 3.31 ppm). Headspace FTIR spectra were recorded from samples in sealed 1 cm quartz cuvettes on a JASCO-4700 spectrometer with a resolution of 8 cm^–1^. The concentration of CO_2_ released was quantified using a calibration curve, as described in earlier studies.^48^ HPLC separations were performed on an Agilent 1100 series instrument equipped with an analytical XB-C18 Kinetex 5 μm, 250 mm × 4.6 mm column. The UV detector was set at λ = 250 nm. A water/acetonitrile mixture (60:40) was employed as the eluent, with a constant flow rate of 0.7 mL/min. Decomposition products were identified by comparing their retention times with those of authentic compounds under the same separation conditions.

Raman spectra at 785 nm were recorded using a 500 mW laser (Cobolt Lasers) fiber-coupled to an Avantes Raman probe. Collected Raman scattering was returned via a 100 μm multimode optical fiber to a Shamrock 163i spectrograph with a 600 L/mm 830 nm blazed grating and an iDus -420-OE CCD camera (Andor Technology). Spectra were acquired using Andor Solis software. Spectra were calibrated with cyclohexane (ASTM E 1840). Raman spectra at 532 nm (300 mW, Cobolt Lasers) were obtained in a 180^*o*^ backscattering arrangement. Raman scattering was collected, collimated and subsequently refocused via a pair of 2.5 cm diameter plano-convex lenses (*f* = 10 cm). The collected light was filtered using an appropriate long-pass edge filter (Semrock) and dispersed by a Shamrock 300i spectrograph (slit width 80 μm, Andor Technology) with a 1200 L/mm grating blazed at 500 nm and acquired with a DV420A-BU2 CCD camera (Andor Technology). Data were recorded and processed using Solis (Andor Technology), with spectral calibration performed using the Raman spectrum of cyclohexane. Baseline correction was performed for all spectra, and they were normalized to the solvent band of MeOH or TFE. Quantification of O_2_ by headspace operando Raman spectroscopy was performed as described earlier.^23^ ESI-MS analyses were performed with an Advion expression CMS, using electrospray ionization and a single-quadrupole compact MS. GC-MS analyses were performed with an Agilent GC-2010 Plus mass spectrometer (EI at 70 eV) equipped with a fused silica capillary column (30 m × 0.25 mm × 0.25 μm) coated with a dimethylsiloxane film (HP-5MS). Decomposition products were identified by comparing their GC retention times with those of authentic compounds. Formaldehyde was quantified colorimetrically as described earlier.^22^

Emission lifetimes were measured on an Edinburgh Instruments FS-5 spectrofluorometer by MCS (multichannel scaling) with a 450 nm (EPL-450) laser diode (Edinburgh Instruments). NIR emission spectra were recorded with a 450 nm laser (45 mW, Power Technology) directed into the optical path of the spectrometer with a 45° long-pass dichroic beamsplitter and focused into the sample with a 35 mm focal length, 25 mm diameter lens. Emission was collected by the same lens, passed through the dichroic beamsplitter and a long-pass filter (1064 nm, Semrock) to remove visible light, and focused with a 35 mm focal length plano-convex lens into a Shamrock 193i spectrograph equipped with an iDus -InGaAs diode array (Andor Technology) with a 600 L/mm grating blazed at 860 nm.

### Reactions with **1** and Peracids

A solution of 1 mM **1** in 1.5 mL of methanol was added to a 1 cm path length cuvette equipped with a screw-cap, septum, and stirring system. While recording UV/visible absorption, 10 equiv of PAA, 10 equiv of PhPAA, and 3.2 equiv of H_2_O_2_, or 10 equiv of PhPAA alone, were added, and the reactions were monitored over 30 min. For the final experiment, FTIR absorption spectra were recorded simultaneously, and the reaction mixture was analyzed by HPLC afterward.

A solution of 1 mM **1** in 1.5 mL of methanol or TFE was added to a 1 cm path length cuvette equipped with a screw-cap, septum, and stirring system. While concurrently recording UV/visible absorption and headspace and liquid-phase Raman spectra at 785 nm, or UV/visible absorption and FTIR absorption spectra, 100 equiv. of PhPAA were added through the septum to the cuvette. The reaction was monitored over 2 h. Headspace Raman scattering was corrected for air in the portion of the confocal volume outside the cuvette. The reaction mixture was analyzed by ^1^H NMR after. Similarly, reactions were performed under an argon or ^18^O_2_ atmosphere. For the final reaction, the mixture was analyzed by ^1^H NMR and GC-MS. For the ESI-MS analysis, while monitoring the reaction with a UV/visible spectrophotometer, a sample was taken when **5** reached its maximum absorbance, after ca. 150 s from the beginning of the reaction, diluted 100 times in MeOH, and directly injected into the ESI source.

### Reactions with **4** and Phenol or Formaldehyde

A solution of 1 mM of **4**, independently prepared^9^ in 1.5 mL MeOH or TFE, was added to a 1 cm path length cuvette with a screw-cap, septum, and stirring system. While recording UV/visible absorption, 10 equiv. of formaldehyde or phenol were added, and the reaction was monitored over 30 min.

### Lifetime and Singlet Oxygen Measurements

A stock solution of 0.2 mg/mL of [Ru(ph_2_phen)_3_](PF_6_)_2_, where ph_2_phen is 4,7-diphenyl-1,10-phenanthroline, was prepared in MeOH and added to a solution of **1** in a 1 cm path length cuvette. While recording the lifetime or Raman spectra, 100 equiv. of PhPAA were added through the septum to the cuvette. The volume and concentration of the three stock solutions were adjusted so that the cuvette had no headspace left, and the final concentrations of the species in the cuvette were 2.7 × 10^–5^ M, 1 mM, and 100 mM for the Ru probe, **1**, and PhPAA, respectively.

### Multivariate Curve Resolution Analysis of UV/Visible Absorption Spectra

Multivariate curve resolution (MCR) was conducted in Python using the pymcr package^49^ in combination with non-negative least-squares (NNLS) regression and a constraint that, after each iteration, the sum of the components involved for each data point sums to one (ConstraintNonneg, ConstraintNorm) (Figure S58). These restrictions are reasonable for spectroscopic usage as long as all species involved have a nonzero contribution to the data set.

Prior to the MCR analysis, a singular value decomposition (SVD) of the dataset was performed to obtain an initial guess of the component spectra needed to resolve the dataset using MCR analysis. SVD is a mathematical operation that reduces a dataset to the minimal set of components that can describe most of the data. The importance of these components is ranked using the singular values. SVD requires neither parameters nor optimization, and the component spectra can be negative, so it is only used for an initial guess. Therefore, the initial guess for the component spectra from the SVD calculation is kept positive, as negative values as input into the MCR are not allowed.

The results from SVD were also used to estimate the number of component spectra needed to resolve the dataset. Specifically, the number of components was plotted against the importance of each component in describing the data. The number of components is chosen such that when a certain number of components describe 99% of the dataset (i.e., the singular value of only one component is taken as 100%), this number of components is used as an input for the MCR analysis, i.e., the number of components to which the MCR analysis is restricted.

The MCR analysis resulted, in this case, in three or four component spectra and how these component spectra change over time with respect to each other (from now on called ″traces over time″). Note that these component spectra are not actual spectra of iron species but merely mathematical shapes that describe the UV/vis data set. These component spectra do resemble those of the iron species, as indicated in the figures. The traces over time of the component spectra were converted to concentration, as described below. For the component spectrum resembling Fe(IV)=O, the absorbance of the component spectrum at 695 nm was multiplied by its traces over time. The resulting absorbance of Fe(IV)=O over time was converted to concentration by the Lambert–Beer law using a molar absorptivity of 400 M^–1^·cm^–1^. For the component spectrum resembling an Fe(III)–phenolato complex, the absorbance of the component spectrum at 580 nm was multiplied by its traces over time. The resulting absorbance of the Fe(III)-phenolato complex over time was converted to concentration by the Lambert–Beer law using a molar absorptivity of 1000 M^–1^·cm^–1^.^50^

The fitted dataset was reconstructed for comparison with the original UV/vis dataset. This was done by multiplying the absorbance of a component spectrum at a certain wavelength by the trace of this component at a certain time point, and the resulting values for each of the three or four components were summed. This was done for each wavelength and at each time point. This process can also be described as matrix multiplication of the component spectra by the traces over time. The outcome of this calculation is the reconstructed dataset and is represented in a color mesh figure. The difference between this reconstructed dataset and the original dataset is represented in the residual color mesh figure. This difference is also presented as a percentage of the original absorbance to show what percentage of the original dataset is not accounted for by the fitted data.

### Kinetic Modeling

Numerical simulation was performed in Python using the “odeint” module from the “scipy.integrate” package. The set of elementary reaction steps and Python scripts is reported as (Figures S59 and S60). The kinetic model was constructed based on the reaction of 1 mM **1** with 100 mM PhPAA in methanol under argon, according to the elementary steps depicted in Scheme 3 using the corresponding rate constants: *k*_1_ (10 M^–1^·s^–1^), *k*_2_ (50 M^–1^·s^–1^), *k*_3_ (18 M^–1^·s^–1^), *k*_4_ (1 × 10^9^ M^–1^·s^–1^), *k*_4–2_ (5 M^–1^·s^–1^), *k*_5_ (10 M^–1^·s^–1^), and *k*_6_ (2.1 × 10^–3^ s^–1^). *k*_6_ was reported^23^ and other rate constants were estimated based on the best fit with the experimental data. In the kinetic model, ligand exchange processes of Fe(II) and Fe(III) species were not taken into account. The concentrations of Fe(IV)=O and Fe(III)–phenolato (**5**) were determined as described in the experimental section of the MCR analysis. The concentrations of phenylperacetic acid (PhPAA), phenylacetic acid (PhAA), and benzyl alcohol were determined from the area underneath their corresponding Raman bands at 885 cm^–1^, 1710 cm^–1^, and 752 cm^–1^, respectively (see also Raman spectra of the liquid phase, Figures S61, S62, and S63). These reaction progress measurements from Raman spectroscopy were calibrated to concentrations using single-point calibration based on the ^1^H NMR spectroscopic data (Figure 2), where the integrated Raman intensities of PhAA and benzyl alcohol between 300 and 500 s were calibrated to 52 and 44 mM, respectively. The CO_2_ concentration was determined with headspace FTIR spectroscopy (Figure S46) from the absorbance at 2360 cm^–1^, as described earlier.^48^

## Supporting Information Available

Supporting Information Additional experimental data, supplementary data for characterization of species **5**, discussion on the difference in reactivity of **1** with 10 equiv. peracids with and without H_2_O_2_, description of luminescence experiments and discussion on a kinetic model in which methanol oxidation is not considered (PDF)
