# Supplementary material for: A Transient, Highly Reactive Fe(IV)=O Species Revealed Through the Interference by O2 in the Activation of Organic Peracids by [(N4Py)Fe(II)]2+
Source: ACS Catal. 2025 Apr 21;15(9):7482–95. doi: 10.1021/acscatal.5c00706 (PMC12054616; doi:10.1021/acscatal.5c00706)
Supplement: Supplementary file 1 — cs5c00706_si_001.pdf [file cs5c00706_si_001.pdf]

A transient highly reactive Fe(IV)=O species revealed through the interference by O_2 in the activation of organic peracids by $[(N4Py)Fe(II)]^{2+}$

Marika Di Berto Mancini,[*,a†] C. Maurits de Roo,[a†] Andy S. Sardjan,[a] Ronald Hage,[a] Giorgio Olivo,[b] Osvaldo Lanzalunga,[b] Marcel Swart,[c,d] Wesley R. Browne[*,a]

[a]Stratingh Institute for Chemistry, Faculty of Science and Engineering, University of Groningen, Nijenborgh 3, 9474AG Groningen, the Netherlands

[b] Dipartimento di Chimica and Istituto CNR per i Sistemi Biologici (ISB-CNR), Università di Roma

"La Sapienza", P.le A. Moro, 5 I-00185 Rome, Italy [c]IQCC and Department of Chemistry, Universitat de Girona, ParcUdG, c/ Emili Grahit 91, 17003, Girona, Spain

[d]ICREA, Pg. Lluís Companys 23, 08010 Barcelona, Spain

[†] Equal contribution

email: m.di.berto.mancini@rug.nl, w.r.browne@rug.nl

Supporting information

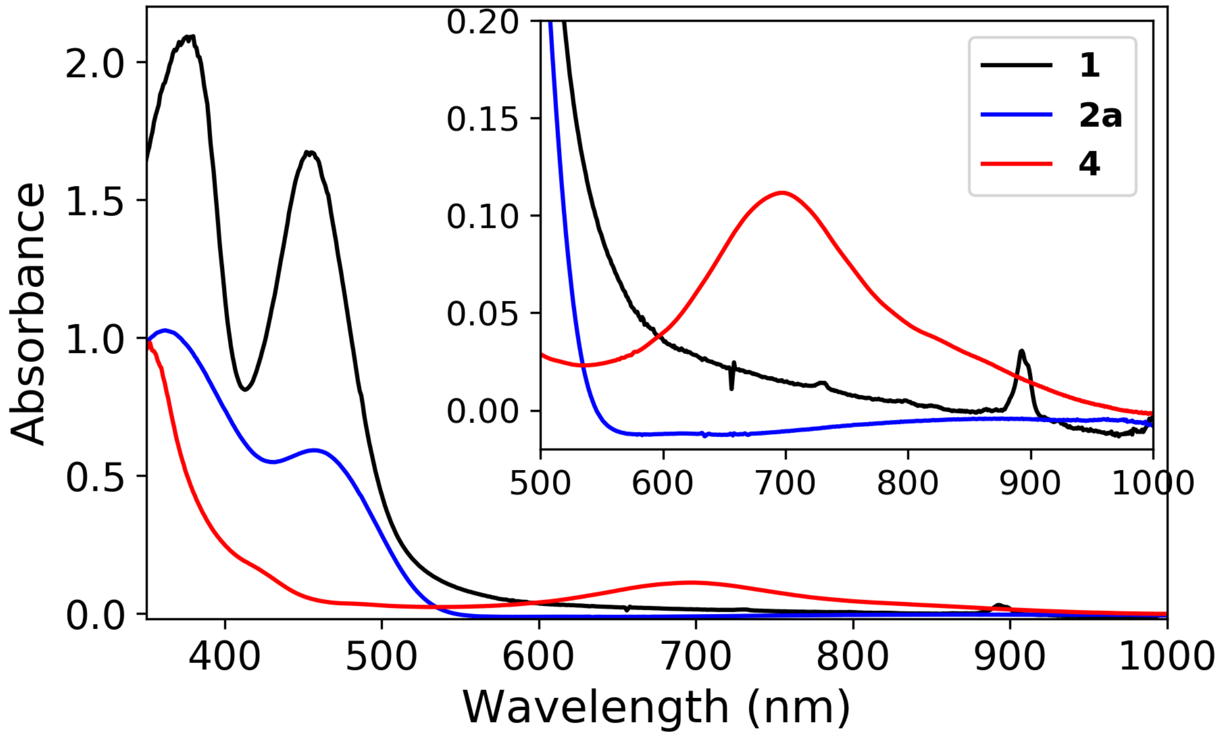

Figure S1: UV/vis absorption spectra of **1** (0.25 mM) in CH_3OH with 2% CH_3CN, **2a** and **4** inCH_3OH .

1 with peracids: comparison between PAA and PhPAA

Comparison of the changes in the UV/vis absorption spectrum of **1** with PAA, and with PhPAA and H_2O_2, shows in both cases Fe(IV)=O and Fe(III)-OOH are formed within several seconds (Figure S2). With 10 equiv. PAA (Figure S2A) the absorption band of Fe(IV)=O forms rapidly in a small extent and then decreases over 10 s with a concurrent increase in the absorption band of Fe(III)-OOH. Thereafter a change is observed in which the absorbance of the Fe(III)-OOH species decreases with a subsequent increase in the absorbance of Fe(IV)=O. After reaching a maximum, the absorption band of Fe(IV)=O decays slowly. These time dependent changes can be rationalized by the fast reaction of **1** with the residual H_2O_2 present in PAA (i.e., rapid formation of a small amount of **4**, which is then reduced by HAT from H_2O_2 with subsequent formation of Fe(III)-OOH). Once the H_2O_2 has been consumed,

reaction of the Fe(III) species present with the peracid yields **4**. With 10 equiv. PhPAA + 3.2 equiv. H_2O_2 (Figure S2B), however, the absorbance of the Fe(III)-OOH species initially decreases faster than with PAA after which it reforms again. The Fe(IV)=O, on the other hand, steadily decreases over 200 s. These results show similar reactivity with PAA, and with PhPAA with H_2O_2, with the difference that in the latter case the reaction of **1** with PhPAA is faster than the reaction with H_2O_2, while in the former the reaction with the hydroperoxide is faster than the reaction with the peracetic acid. When 10 equiv. PhPAA only (i.e. no H_2O_2) is added, clean conversion to **4** is observed (Figure S2C).

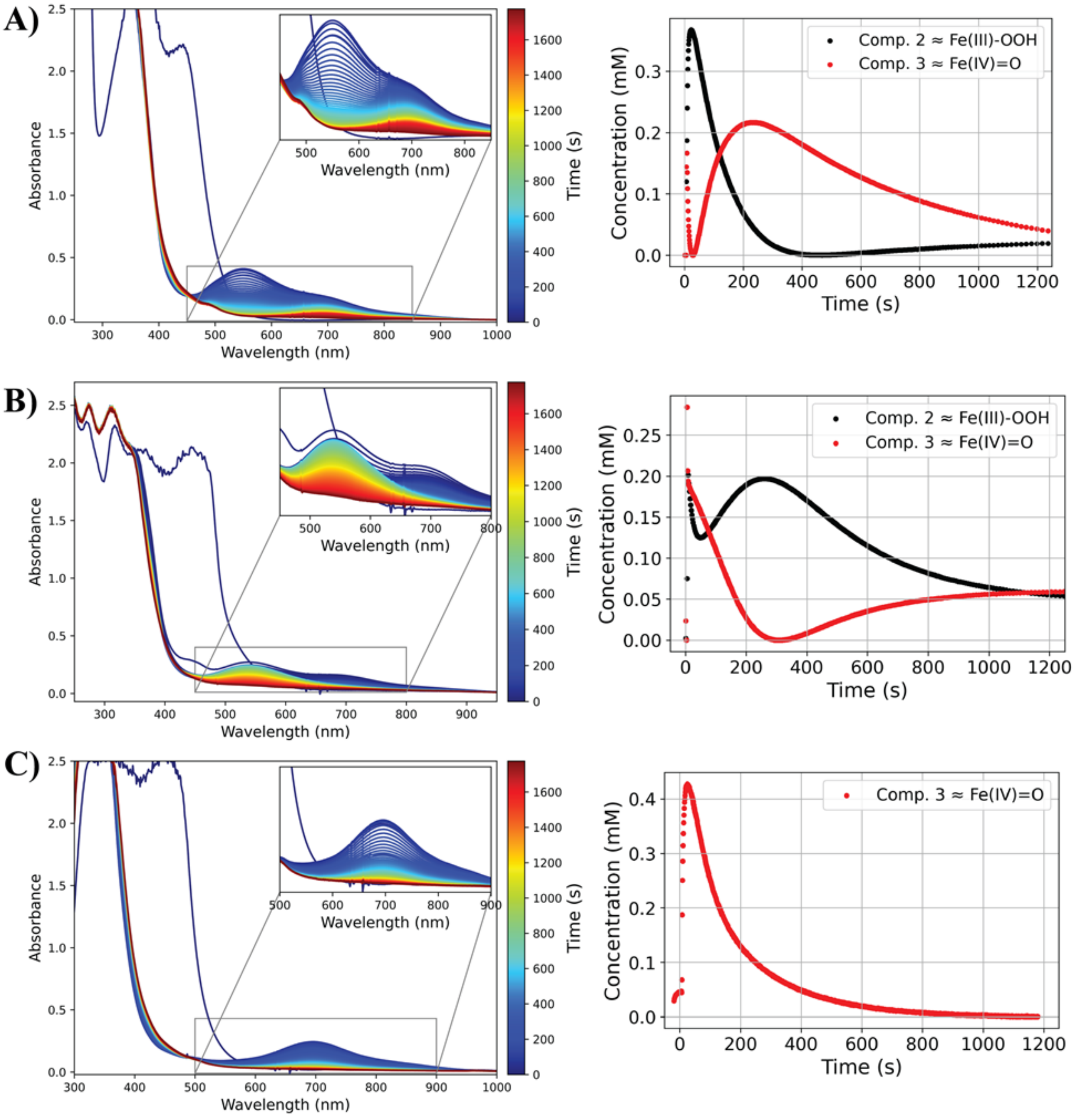

Figure S2: UV/vis absorption spectra over time of **1** (1 mM) in CH_3OH with (A) 10 equiv. of PAA, (B) 10 equiv. of PhPAA + 3.2 equiv. of H_2O_2, and (C) 10 equiv. of PhPAA. Insets show the characteristic bands of the Fe(III)-OOH (at 550 nm) and Fe(IV)=O (at 695 nm) species. Right panels show the concentration of Fe(III)-OOH and Fe(IV)=O over time according to MCR analysis of the spectra (Figures S3, S5, and S7).

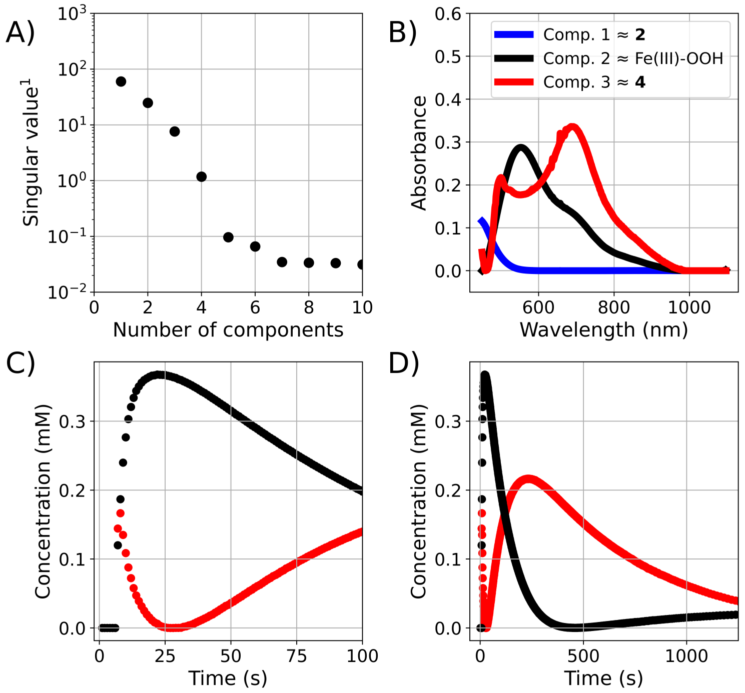

Figure S3: Multivariate curve resolution (MCR) of the time dependent UV/vis absorption data from the reaction of 1 mM **1** with 10 eq. PAA in MeOH using three components to resolve the dataset. A) Number of components that the singular value decomposition (SVD) uses to resolve the dataset.[1] The singular value is related to the importance of this component to resolve the dataset; the smaller the singular value, the less important the corresponding component is in resolving the dataset. B) The three component spectra ('shapes') with their relative absorbance: Comp. 1 (**2**, blue), Comp. 2 (Fe(III)-OOH, black), and Comp. 3 (**4**, red). C) and D) The concentration of comp. 2 (Fe(III)-OOH) and comp. 3 (**4**) over 100 s and 1400 s, respectively.

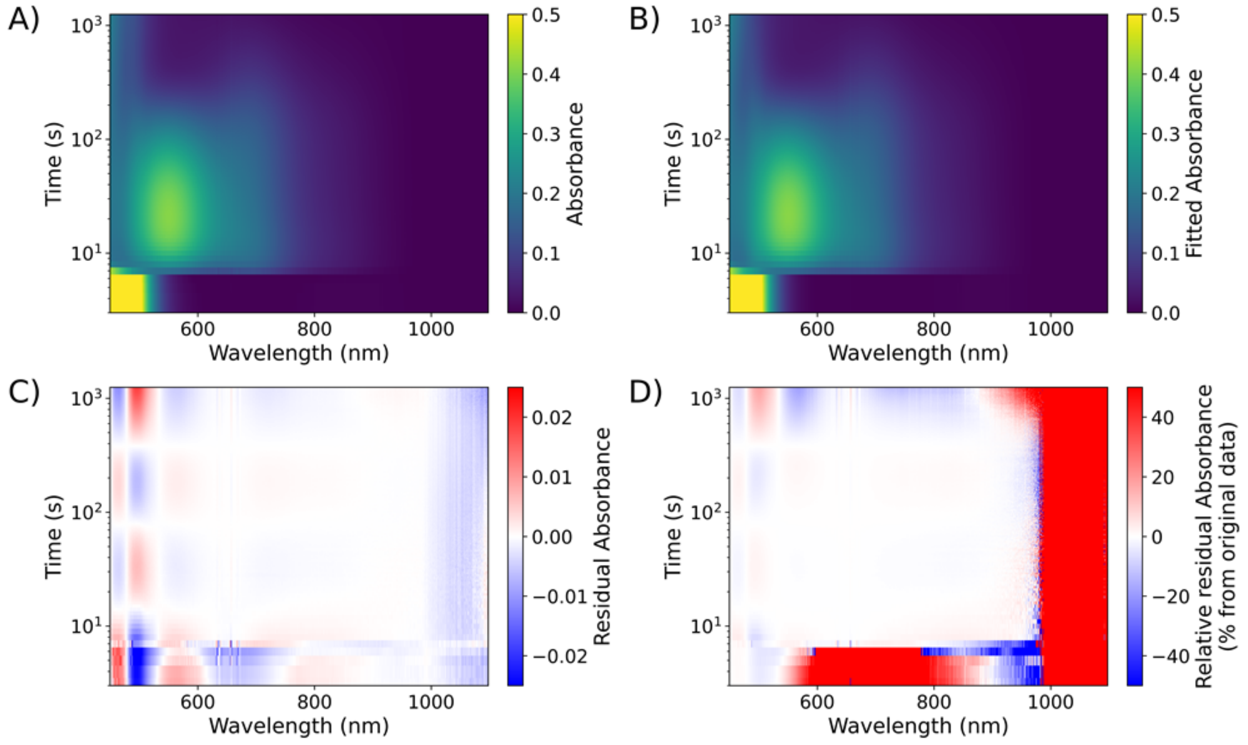

Figure S4: Multivariate curve resolution (MCR) of the time dependent UV/vis absorption data from the reaction of 1 mM **1** with 10 eq. PAA in MeOH using three components to resolve the dataset. A) and B) depict the representation of the original data and fitted data, respectively. C) and D) show the residual absorbance (actual absorbance minus fitted absorbance) and the residual absorbance as percentage of the actual absorbance, respectively. It is of note that in Figures A) and B) the absorbance is scaled between 0-0.5 to show features relevant to the iron complexes with good contrast. However, as a result the **2** that is present initially ($t < 8$ s) is misrepresented as having an absorbance of 0.5, instead of ca. 2.

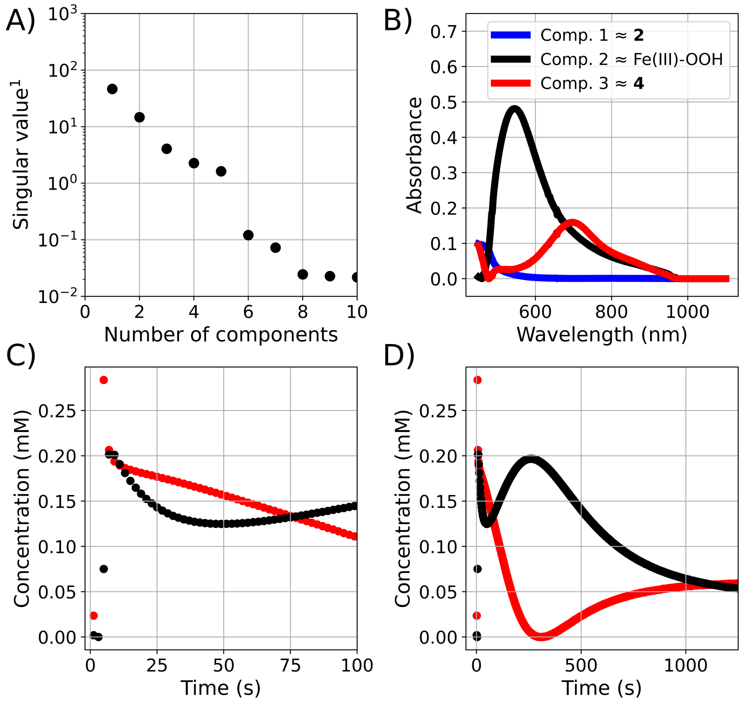

Figure S5: Multivariate curve resolution (MCR) of the time dependent UV/vis absorption data from the reaction of 1 mM **1** with 10 equiv. of PhPAA + 3.2 equiv. of H_2O_2 in MeOH using three components to resolve the dataset. A) Number of components that the singular value decomposition (SVD) uses to resolve the dataset.[1] The singular value is related to the importance of this component to resolve the dataset; the smaller the singular value, the less important the corresponding component is. B) The three component spectra ('shapes') with their relative absorbance: Comp. 1 (**2**, blue), Comp. 2 (Fe(III)-OOH, black), and Comp. 3 (**4**, red). C) and D) The concentration of comp. 2 (Fe(III)-OOH) and comp. 3 (**4**) over 100 s and 1500 s, respectively.

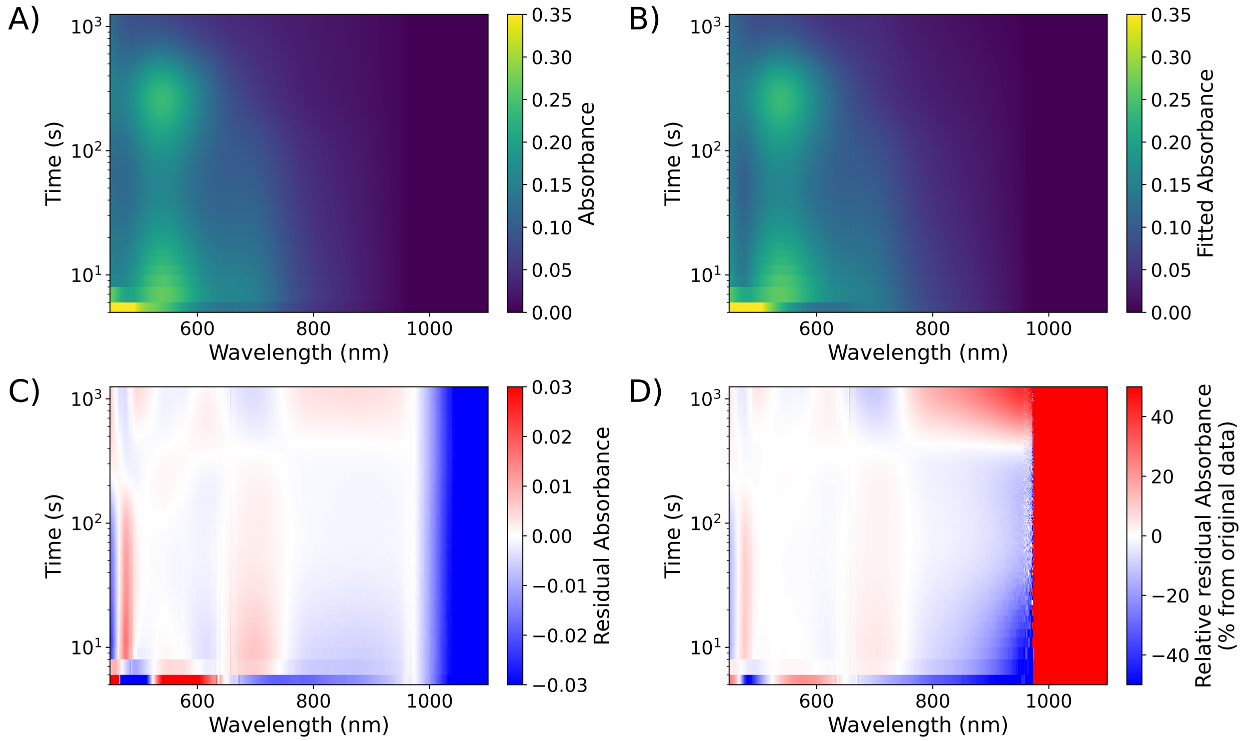

Figure S6: Multivariate curve resolution (MCR) detailed results of the UV/vis spectroscopy measurement of the reaction of 1 mM **1** with 10 equiv. of PhPAA + 3.2 equiv. of H_2O_2 in MeOH using three components to resolve the dataset. A) and B) depict the representation of the original data and fitted data, respectively. C) and D) show the residual absorbance (actual absorbance minus fitted absorbance) and the residual absorbance as a percentage of the actual absorbance, respectively. It is of note that in Figures A) and B) the absorbance is graphed between 0-0.35 to show features relevant to the iron complexes with good contrast. However, as a result the **2** that is present initially ($t < 5$ s) is misrepresented as having an absorbance of 0.5, instead of ca. 2.

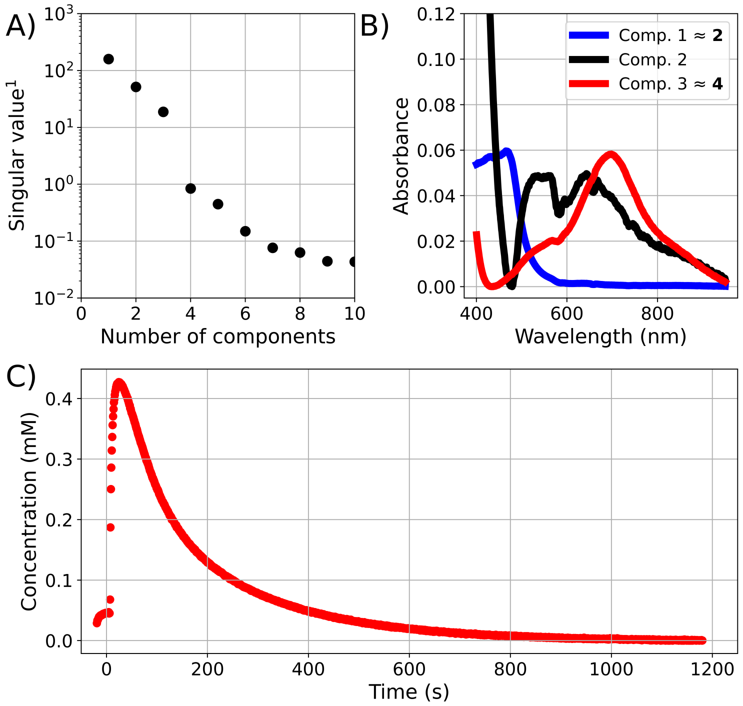

Figure S7: Multivariate curve resolution (MCR) of the time dependent UV/vis absorption data from the reaction of 1 mM **1** with 10 equiv. of PhPAA in MeOH using three components to resolve the dataset. A) Number of components that the singular value decomposition (SVD) uses to resolve the dataset.[1] The singular value is related to the importance of this component to resolve the dataset; the smaller the singular value, the less important the corresponding component is. B) The three component spectra ('shapes') with their relative absorbance: Comp. 1 (**2**, blue), Comp. 2 (not a relevant shape for the analysis, black), and Comp. 3 (**4**, red). C) The concentration of Comp. 3 (**4**) over 1200 s.

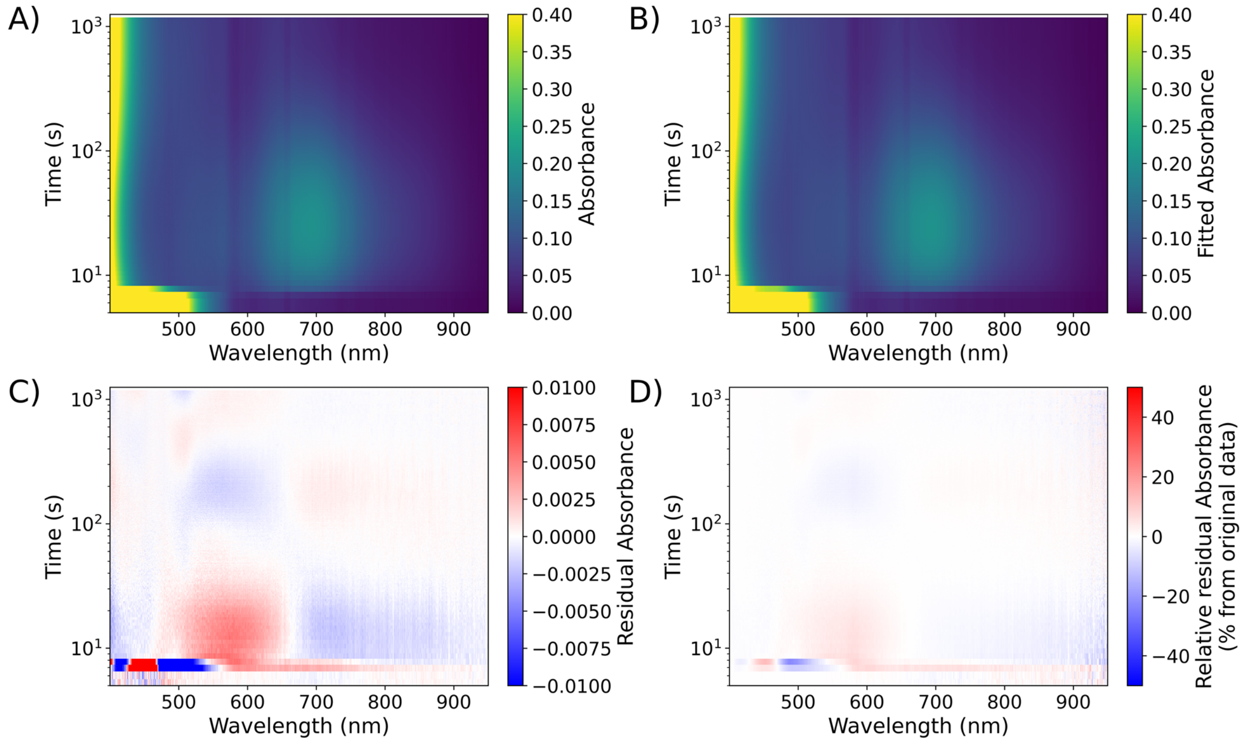

Figure S8: Multivariate curve resolution (MCR) detailed results of the UV/vis spectroscopy measurement of the reaction of 1 mM **1** with 10 equiv. of PhPAA in MeOH using three components to resolve the dataset. A) and B) depict the representation of the original data and fitted data, respectively. C) and D) show the residual absorbance (actual absorbance minus fitted absorbance) and the residual absorbance as percentage of the actual absorbance, respectively. It is of note that in Figures A) and B) the absorbance is graphed between 0-0.4 to show features relevant to the iron complexes with good contrast. However, as a result the **2** that is present initially (t $<$ 8 s) is misrepresented as having an absorbance of 0.5, instead of ca. 1.5.

1 with 10 equiv. phenylperacetic acid

Monitoring of the reaction of 10 equiv. of PhPAA with **1** in methanol simultaneously by UV/vis absorption and headspace FTIR spectroscopy (Figure S9) reveals that the characteristic visible absorption of **2a** disappears immediately within the mixing time with concomitant appearance of the NIR absorption band of **4**, corresponding to 50% of the initial concentration of **1**. After 30 s, the NIR absorbance of **4** begins to decrease and decreases over the subsequent 10 min. Headspace FTIR spectroscopy shows evolution of CO_2 from the reaction mixture over the same period (corresponding to 10% with respect to the PhPAA added, Figure S10 for details). HPLC analysis of the reaction mixture after 30 min shows the presence of phenylacetic acid (corresponding to 90% of the initial PhPAA added) with 10% benzaldehyde and no residual PhPAA (Figure S11). Overall, such distribution of products does not reflect the expected one, that would lead to ca. 90% benzyl-radical derived products and ca. 10% phenylacetic acid.

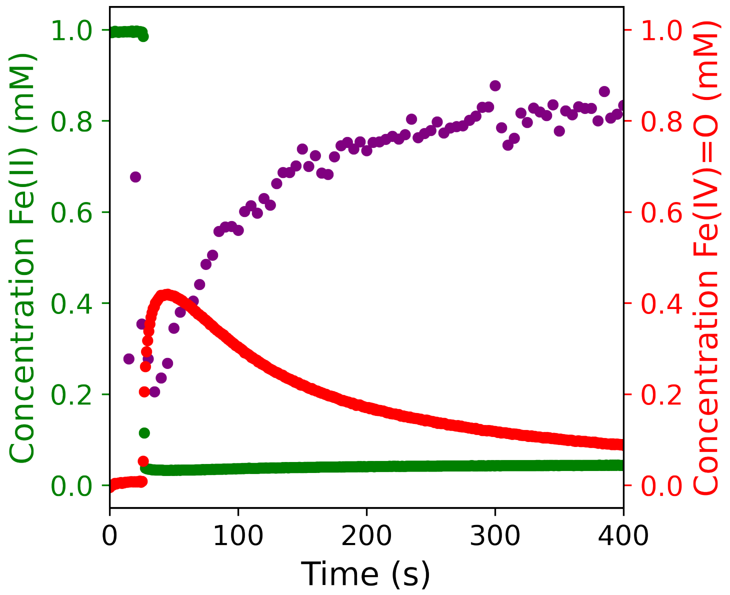

Figure S9: Time dependence of absorbance at 450 nm (Fe(II), green) and 695 nm (Fe(IV)=O, red) and headspace CO_2 (data normalized to maximum absorbance at 2360 cm^{-1}, purple). Conditions: 1 mM **1** to which 10 mM PhPAA was added with rapid mixing.

The initial formation of **4** concomitant with the disappearance of the Fe(II) complex is

consistent with formation of an intermediate Fe(II)−OOC(O)CH_2Ph species which undergoes rapid O-O bond heterolysis (Step 1 scheme 1).

The extent of formation of **4** is potentially limited by its comproportionation with any remaining Fe(II) complex to yield **3b** or **3a** (Step 2 in scheme 1). Instead, the reaction of **4** with solvent, to form the **3b** species and formaldehyde, is relatively slow, especially in CD_3OD.

$[(N4Py)Fe(III)\text{-}OH/OCH_3]^{2+}$ is expected react with PhPAA, forming a putative [(N4Py)Fe(III)-OOC(O)CH_2Ph]$^{2+}$ species, and following O-O bond homolysis, forms **4** and a carboxyl radical (Step 3 in scheme 1). The subsequent decomposition of the carboxyl radical liberates CO_2, which is observed by headspace FTIR spectroscopy. The CO_2 evolved appears to indicate that the homolytic pathway between Fe(III)-OH and the peracid is possible, yielding the peroxo radical that can decompose into organic radical products, such as benzyl alcohol, benzaldehyde, and/or benzoic acid, and CO_2. However, several observations indicate that the mechanism is more complex.

In fact, quantification of the CO_2 evolved and the other products expected for radical decomposition shows that only 10% of the PhPAA is decomposed in this way. In addition, the evolution of CO_2 over time shows a maximum is reached within ca. 200 s, corresponding to the time in which the NIR absorption of **4**, formed initially, decays. Furthermore, HPLC analysis shows the presence of 90% phenylacetic acid and only 10% benzaldehyde. Taking into account all these information, the decomposition of PhPAA by **1** does not seem to proceed primarily by the homolytic pathway (Step 3 in scheme 1) as expected, but rather by an alternative pathway that yields phenylacetic acid as the main product.

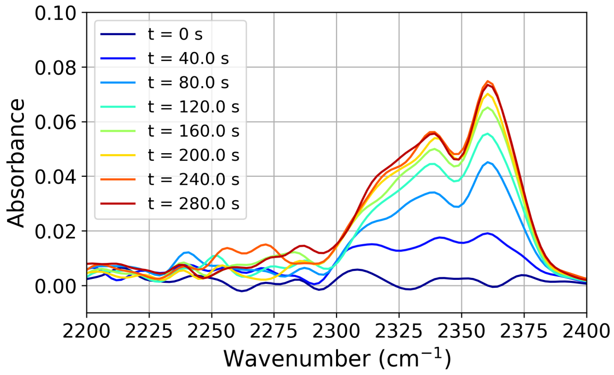

Figure S10: FTIR spectra (headspace) from 0 to 500 s of the reaction of 10 equiv. phenylperacetic acid with **1**. Conditions: 1 mM **1**, 10 mM phenylperacetic acid in 1.5 mL MeOH.

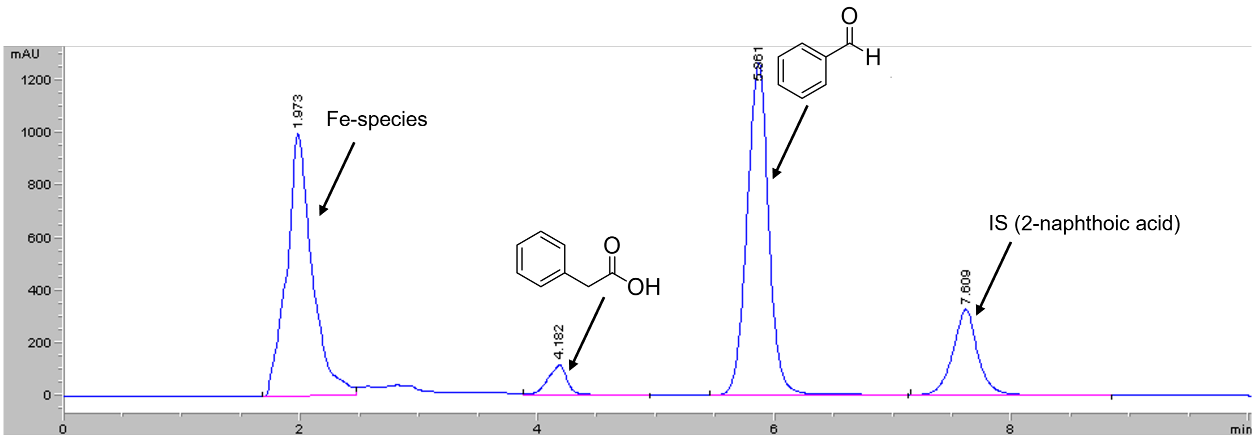

Figure S11: HPLC chromatogram of the reaction mixture 30 min after addition of 10 equiv. PhPAA to **1**. Conditions: 1 mM **1**, 10 mM phenylperacetic acid in 2 mL MeOH.

1 with 100 equiv. phenylperacetic acid in methanol

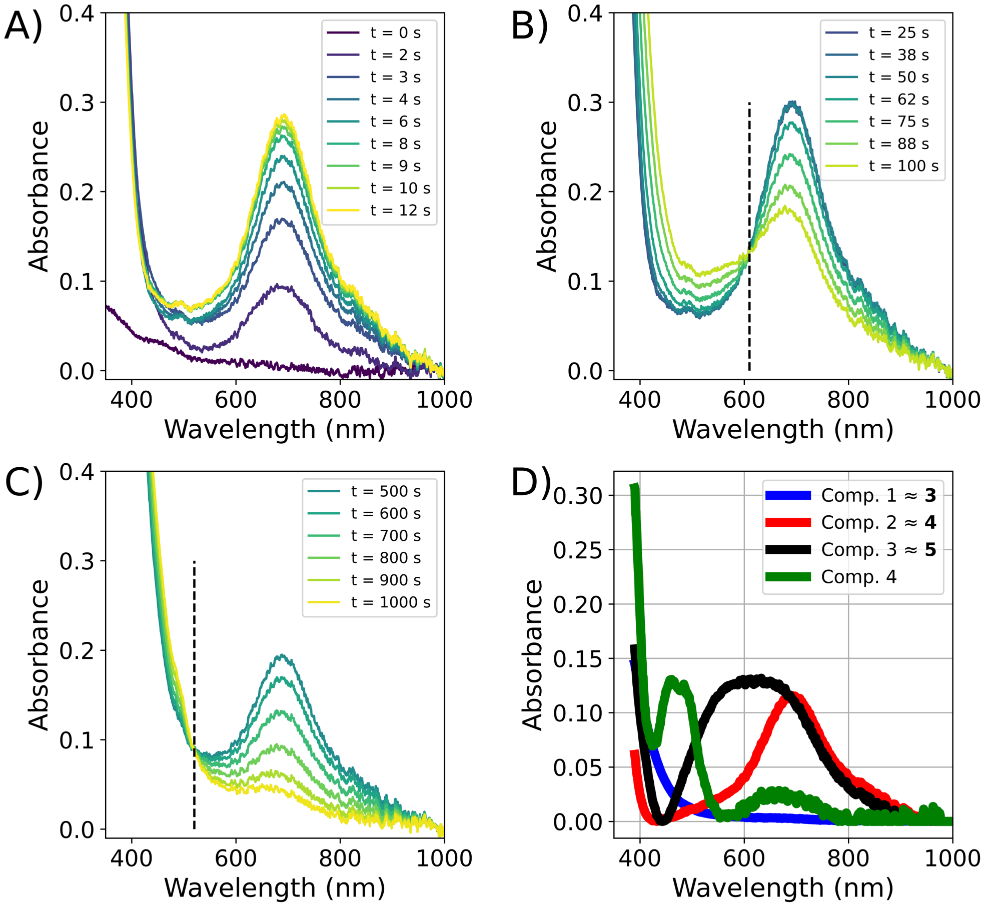

Figure S12: UV/vis absorption spectra of 100 equiv. phenylperacetic acid before and after addition of **1**, A) 0-12 s, B) 25-100 s, and C) 500-1000 s. D) shows the four component spectra obtained from multivariate curve resolution (MCR) analysis of the data. The fourth component does not represent a spectrum that is reasonable for a single species. Conditions: 1 mM **1**, 100 mM PhPAA, in 1.5 mL MeOH.

4 with 10 equiv. formaldehyde

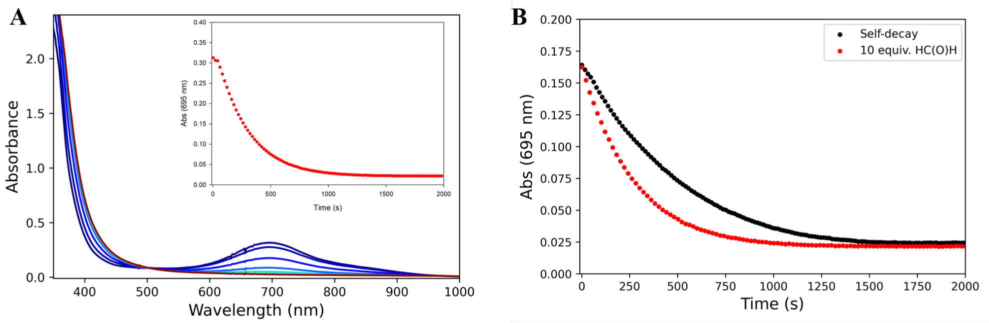

Figure S13: A) UV/vis absorption spectra over time of the reaction of **4** with formaldehyde in MeOH under air. Inset shows the decay in absorbance at 695 nm. Conditions: 1 mM **4**, 10 mM formaldehyde in 1.5 mL MeOH. B) Decay in absorbance at 695 nm in the reaction of **4** and formaldehyde with time is zero at the point where the absorbance is the same as the initial absorbance in the self-decay of **4** in MeOH. A solid sample of independently prepared**4** was used i.e. [(N4Py)Fe(IV)=O]$(PF_6)_2$.[21]

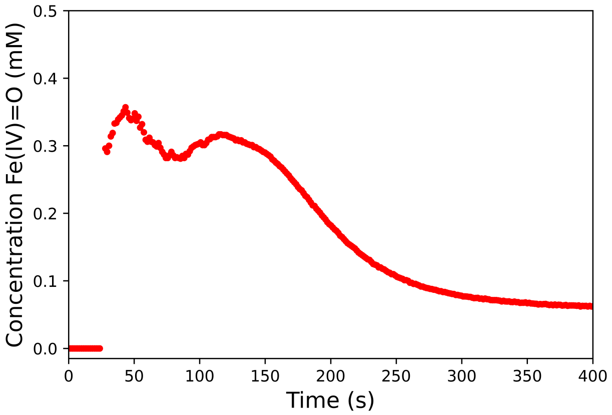

Figure S14: Concentration of **4** over time after the addition of phenylperacetic acid (100 mM) to **1** (1 mM) in MeOH (1.5 mL). The concentration of **4** over time shows differences compared compared to that shown in Figure 4C despite otherwise identical conditions. the differences are due to the difference in stirring and, hence concentration of O_2 in the solution overtime in each case.

Identification of species 5

MCR analysis show the presence of **5** under every reaction condition, with differences in extent and persistence. Under an $^{18}O_2$ atmosphere, **5** is formed to a lesser extent compared to reactions carried out under air and even less compared to reactions under Argon. The greater extent for formaiton of this species under argon compared to under air, suggests that **5** can not be an iron-peroxy species, such as Fe(III)−O−O−C(O)−CH_2−Ph. Moreover, the visible range of the absorption band is typical of an LMCT band in Fe(III) complexes. Assignment of the structure of **5** as Fe(III)−O−CH_2−Ph can be discounted as the calculated UV/vis spectrum of such as species shows a different absorption in the visible range (Figure S15) and mass analysis of the reaction mixture ca. 150 s after addition of PhPAA (monitored with UV/vis analysis, when **5** has reached its maximum of absorbance, see experimental section for details) does not show evidence of an iron-benzyl alcohol complex (see Figure S16). In addition, attempts to identify the species by resonance Raman spectroscopy (λ_{exc} = 532 nm) **5** proved unsuccessful, due to the limited accumulation of the species and a high fluorescence background.

The range of the visible absorption of **5** resembles the LMCT band of the iron-phenolato species formed in the reaction between **4**, independently prepared, and phenol, Figure S17. Indeed, MCR Comp. 2 of the reaction between **1** and PhPAA in TFE, namely **5**, is very similar to the absorption spectrum of iron-phenolato species (Figure S32).

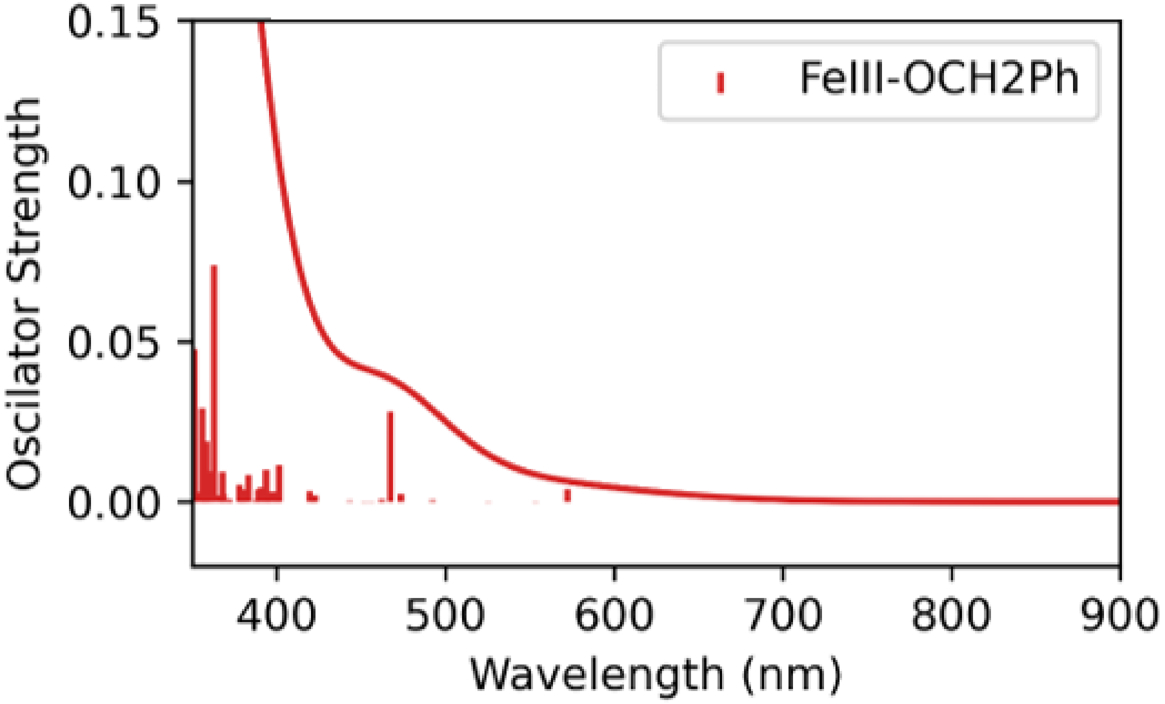

Figure S15: Calculated spectrum of Fe(III)−OCH_2Ph.

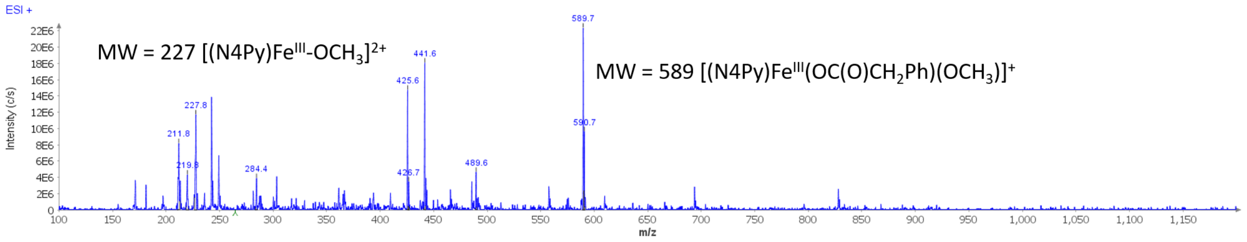

Figure S16: ESI-quadrupole spectrum of reaction at after ca. 150 s.

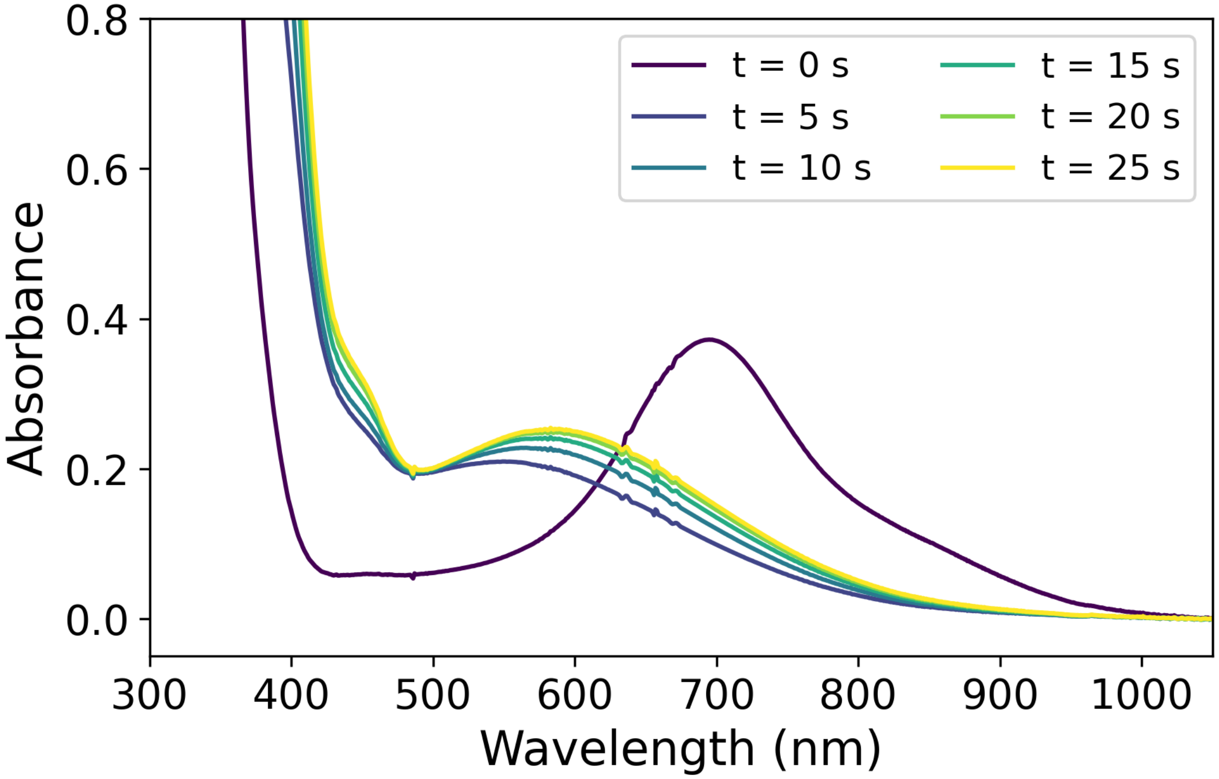

Figure S17: UV/vis absorption spectra over 25 s after addition of phenol to **4** in TFE under air. Conditions: 1 mM **4**, 10 mM phenol in 1.5 mL TFE.

Based on the exclusion of the other possible species involved in the reaction and on the similarity of the UV/vis absorption spectra, our best hypothesis for **5** is an iron-phenolato species. It could be generated after the formation of **4** and the benzyl radical during the reaction (scheme S1) by analogy to that shown elsewhere, (see Unjaroen, Inorg. Chem., 2017, 56, 470-479) where **4** is able to perform aromatic hydroxylation via an electrophilic substitution reaction on substrates such as anthracene; however, experimentally the reaction between **4** and benzene did not occur and the high barriers, calculated by DFT methods, may be the reason. Hence, it is unlikely that during the reaction **4** can directly react with the phenyl ring of the PhPAA. (see de Visser, et al Inorg. Chem. 2007, 46, 4632-4641) Nevertheless, the MCR analysis and the experimental data point towards the formation of an iron-phenolato species, suggesting the evolution of a more reactive species that can hydroxylate the aromatic ring and form **5**. However, as shown by the MCR and the UV/vis analysis, **5** is formed in relative low concentration, so the probability of this reaction is really low. In addition, this species is unstable and eventually it decomposes to other Fe(III) species.

$$\text{PhPAA} + \text{(L)Fe}^{IV}\text{=O } (\mathbf{4}) \rightleftharpoons \text{(L)Fe}^{III}\text{-OPh-R } (\mathbf{5}) \rightleftharpoons \text{Ph-R} + \text{(L)Fe}^{III}\text{-X } (\mathbf{3})$$

Scheme S1: Possible reactions that lead to the formation of **5**.

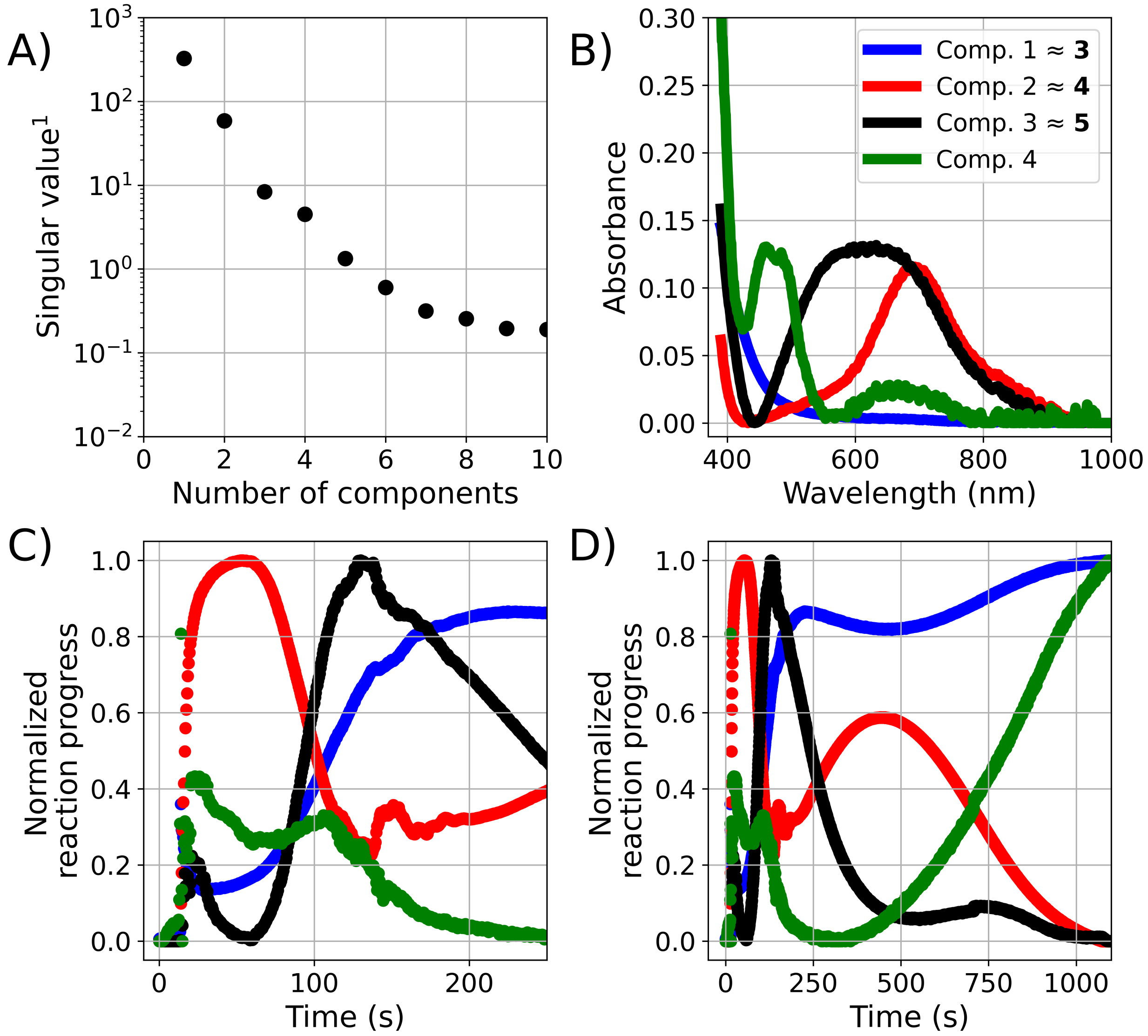

Figure S18: Multivariate curve resolution (MCR) detailed results of the UV/vis spectroscopy measurement of the reaction of 1 mM **1** with 100 eq. PhPAA in MeOH using four components to resolve the dataset. A) Number of components that the singular value decomposition (SVD) uses to resolve the dataset.[1] The singular value is related to the importance of this component to resolve the dataset; the smaller the singular value, the less important is the corresponding component to resolve the dataset. B) The four component spectra ('shapes') with their relative absorbance: Comp. 1 (**3**, blue), Comp. 2 (**4**, red), Comp. 3 (**5**, black), and Comp. 4 (green). C) and D) The relative importance (contribution) of each component at each data point over 250 s and 1100 s, respectively. It should be noted that component concentrations obtained from MCR only represent absorbance contributions over time and are normalized in each case, and thus do not present the actual concentrations of the various iron species present.

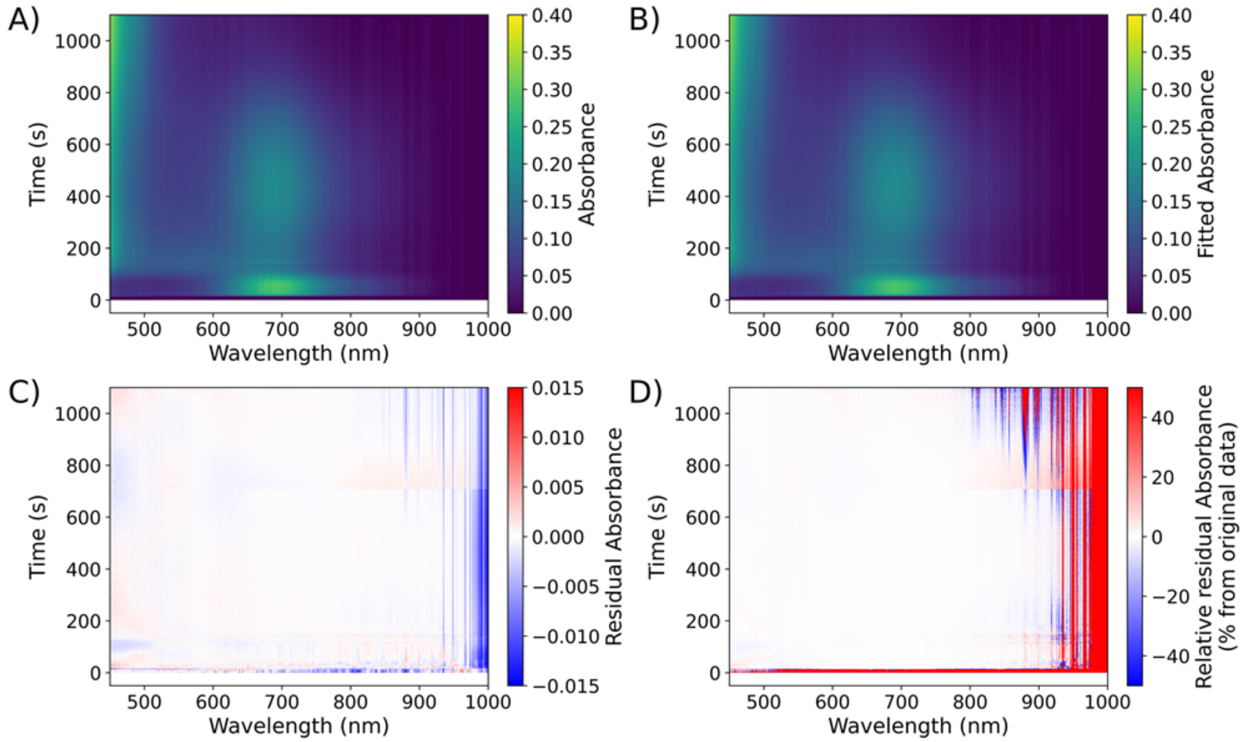

Figure S19: Multivariate curve resolution (MCR) detailed results of the UV/vis absorption spectra during the reaction of 1 mM **1** with 100 eq. PhPAA in MeOH using four components to resolve the dataset. A) and B) depict the representation of the original data and fitted data, respectively. C) and D) show the residual absorbance (actual absorbance minus fitted absorbance) and the residual absorbance as a percentage of the actual absorbance, respectively.

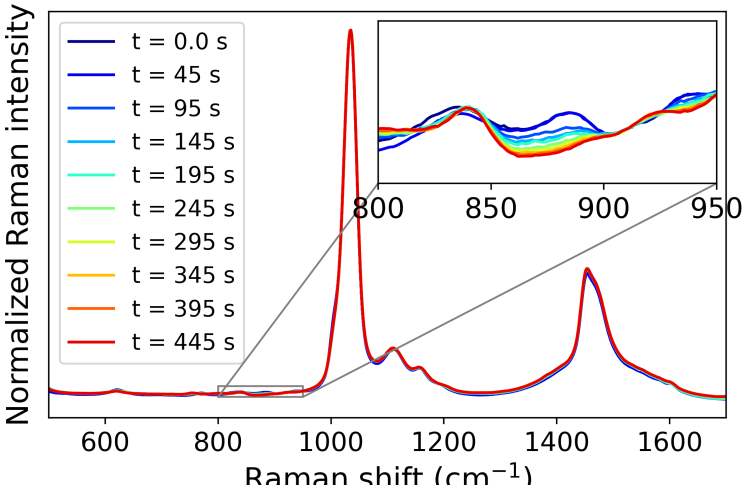

Figure S20: Raman spectra (liquid phase) of the reaction of 100 equiv. phenylperacetic acid with **1**, monitoring the change in PhPAA concentration over time (Raman band 885 cm^{-1}). Conditions: 1 mM **1**, 100 mM phenylperacetic acid, in 1.5 mL MeOH.

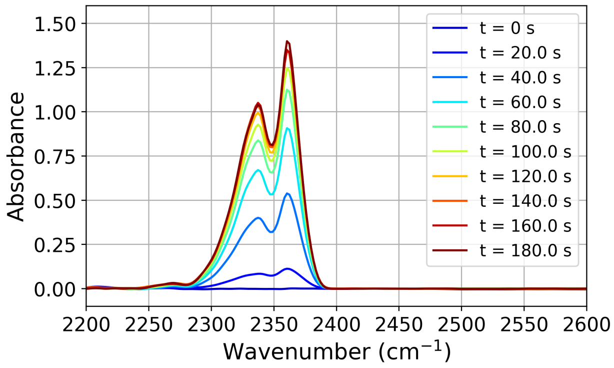

Figure S21: FTIR spectra (headspace) during the reaction of 100 equiv. phenylperacetic acid with **1** (1 mM) in methanol.

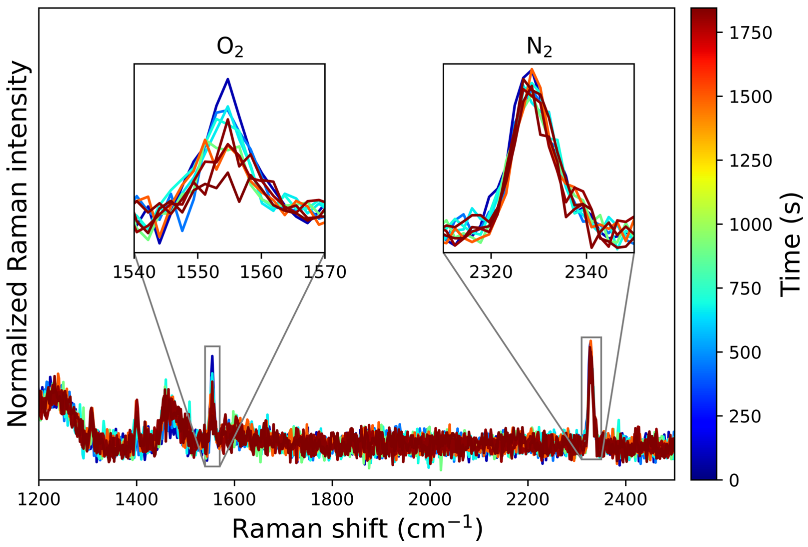

Figure S22: Raman (headspace) analysis of the reaction mixture with 100 equiv. phenylperacetic acid added to **1** (1 mM) in methanol. 0.045 mmol of O_2 are consumed.

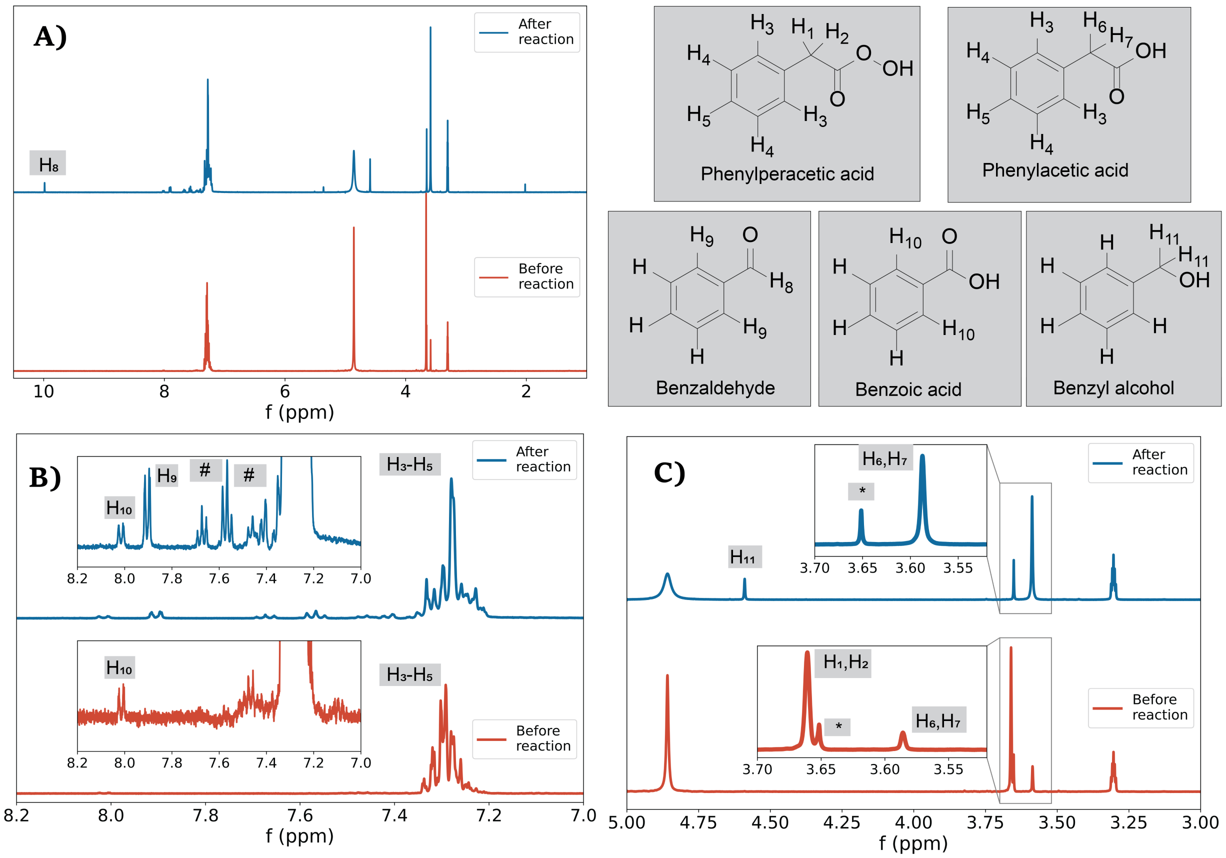

Figure S23: ^{1}H-NMR spectrum of the reaction mixture before (red) and 2 h after (blue) addition of **1** to 100 equiv. PhPAA. Conditions: 1 mM **1**, 100 mM PhPAA in 1.5 mL CD_3OD.

Luminescence spectroscopy

Quenching of the phosphorescence of $[Ru(ph_2phen)_3]^{2+}(PF_6)_2$ by dissolved O_2 through energy transfer is manifested in a reduction of the former's phosphorescence decay lifetime (τ_{obs}). The concentration of O_2 directly proportional to the observed decay rate constant ($k_{obs} = \frac{1}{\tau_{obs}}$) through the Stern-Volmer equation (see Demas et al, J. Am. Chem. Soc., 1977, 99, 3547-3551 and Juris et al, Coord. Chem. Rev., 1988, 84, 85-277). Hence, the concentration of O_2 in the reaction mixture over time following addition of PhPAA can be followed indirectly by the emission decay lifetime of $[Ru(ph_2phen)_3]^{2+}$. Observation of the NIR emission (1268 nm) from the $^1O_2$ generated in the quenching process is a necessary positive control to ensure that quenching reflects O_2 concentration and that it is not influenced by *in situ* formation of other quenchers of the electronically excited $[Ru(ph_2phen)_3]^{2+}$ complex. Hence, changes in intensity of NIR emission from $^1O_2$ can be used to establish that the increase in the emission lifetime of $[Ru(ph_2phen)_3]^{2+}$ is due to a reduction in the concentration of dissolved $^3O_2$.

We determined the concentration of dissolved O_2 over time indirectly through monitoring the O_2 sensitive luminescence decay lifetime of $[Ru(ph_2phen)_3]^{2+}$ and directly from the NIR emission at 1268 nm of $^1O_2$, formed through the quenching of the excited state of $[Ru(ph_2phen)_3]^{2+}$ by $^3O_2$ (see De Roo et al, ChemCatChem, 2024, e202301594).

Addition of excess PhPAA to **1** results a decrease in the concentration and eventually complete consumption of dissolved O_2 manifested in an increase in the emission lifetime of $[Ru(ph_2phen)_3]^{2+}$ from 0.23 μs to ca. 1.5 μs over 200 s (figure S30), indicating essentially complete consumption of dissolved O_2. This was confirmed by the decrease in the sensitized emission of $^1O_2$ (Figure S24). After 200 s, the emission lifetime decreased without concomitant increase in $^1O_2$ emission, indicating the presence of another quencher in the solution, e.g., an Fe(III) complex, that is capable of quenching the excited state of $[Ru(ph_2phen)_3]^{2+}$ by electron transfer (Figure S25) (see Kotani et al, J. Am. Chem. Soc., 2011, 133, 3249-3251 and Company at al, J. Am. Chem. Soc., 2014, 136, 4624-4633).

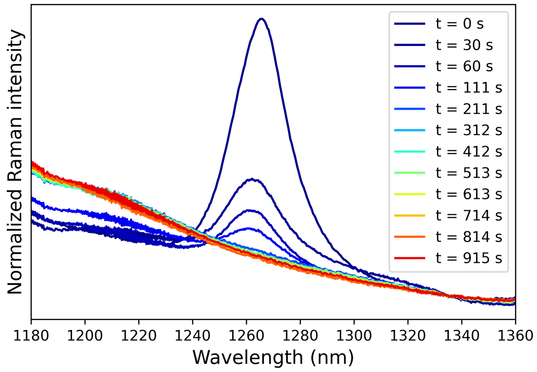

Figure S24: NIR emission from $^1O_2$ under standard reaction conditions over 20 min following addition of PhPAA acid to **1**. Conditions: 1 mM **1**, 100 mM PhPAA, 2.7 x 10^{-5} M $[Ru(ph_2phen)_3]^{2+}$ in 2 mL MeOH.

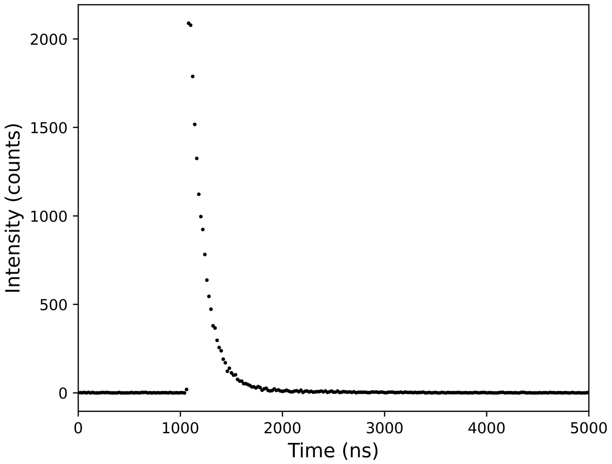

Figure S25: Emission decay of $[Ru(ph_2phen)_3]^{2+}$ in MeOH with **3a** showing a decrease in emission lifetime from ca. 230 ns (only $[Ru(ph_2phen)_3]^{2+}$ in solution) to ca. 135 ns with **3a** present. Conditions: 0.5 mM **3a** independently generated, 2.7 x 10^{-5} M $[Ru(ph_2phen)_3]^{2+}$ in 3 mL MeOH.

Control experiments were carried out with $[Ru(ph_2phen)_3]^{2+}$ together with PhPAA, and with **1** (Figure S26). The presence of PhPAA or **1** does not affect the emission lifetime of $[Ru(ph_2phen)_3]^{2+}$. However, the intensity of the emission from $^1O_2$ is decreased by PhPAA due to the addition of O-H oscillators that facilitate non radiative relaxation of $^1O_2$ (see figures S27 and S28). Re-equilibration of O_2 from the headspace into solution was avoided by filling the cuvette completely.

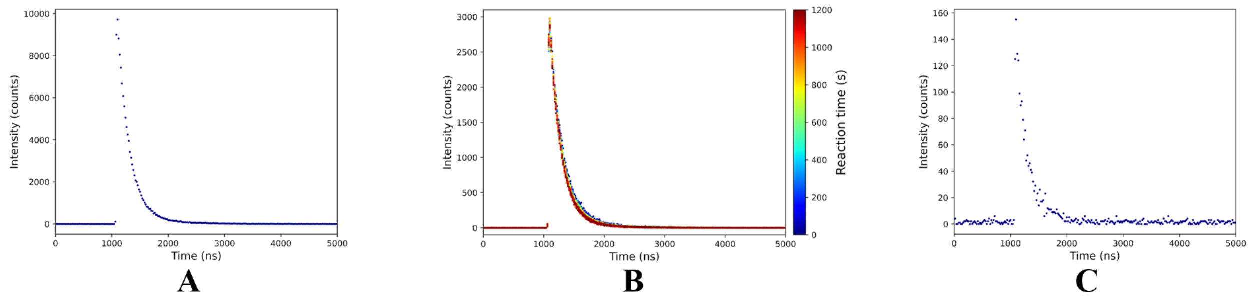

Figure S26: Control experiment of $[Ru(ph_2phen)_3]^{2+}$ lifetime: (A) $[Ru(ph_2phen)_3]^{2+}$ lifetime in MeOH under air; (B) $[Ru(ph_2phen)_3]^{2+}$ lifetime in MeOH under air with PhPAA (100 mM) monitored for 20 min; (C) $[Ru(ph_2phen)_3]^{2+}$ lifetime in MeOH under air with 1 mM **1**. The lifetime of $[Ru(ph_2phen)_3]^{2+}$ is not affected by the two reagent separately, it stays constant at ca. 230 ns. Conditions: 2.7 x 10^{-5} M $[Ru(ph_2phen)_3]^{2+}$ in 2 mL MeOH, 100 mM PhPAA, or 1 mM **1**.

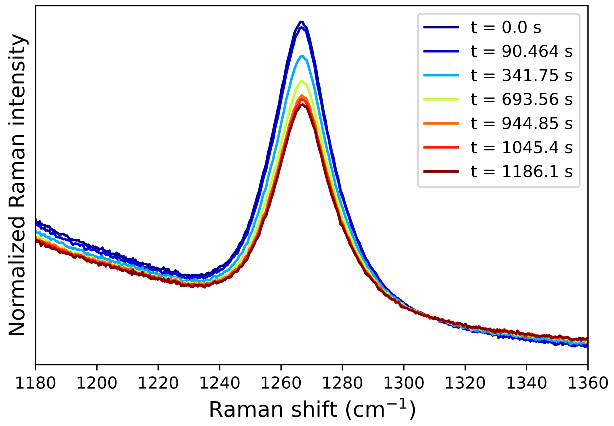

Figure S27: Control experiment of $^1O_2$ NIR emission signal with **1** monitored for 20 min. Conditions: 1 mM **1**, 2.7 x 10^{-5} M $[Ru(ph_2phen)_3]^{2+}$ in 2 mL MeOH.

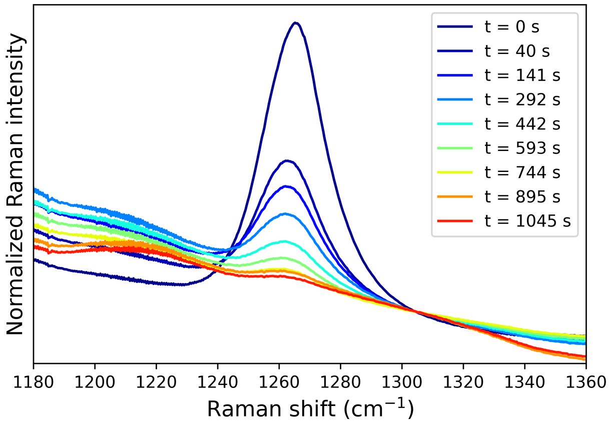

Figure S28: $^1O_2$ NIR emission in the presence of PhPAA monitored over 20 min. The signal decreases initially due to the addition of O-H oscillators, followed by a slower decrease over 1000 s. Conditions: 100 mM PhPAA, 2.7 x 10^{-5} M $[Ru(ph_2phen)_3]^{2+}$ in 2 mL MeOH.

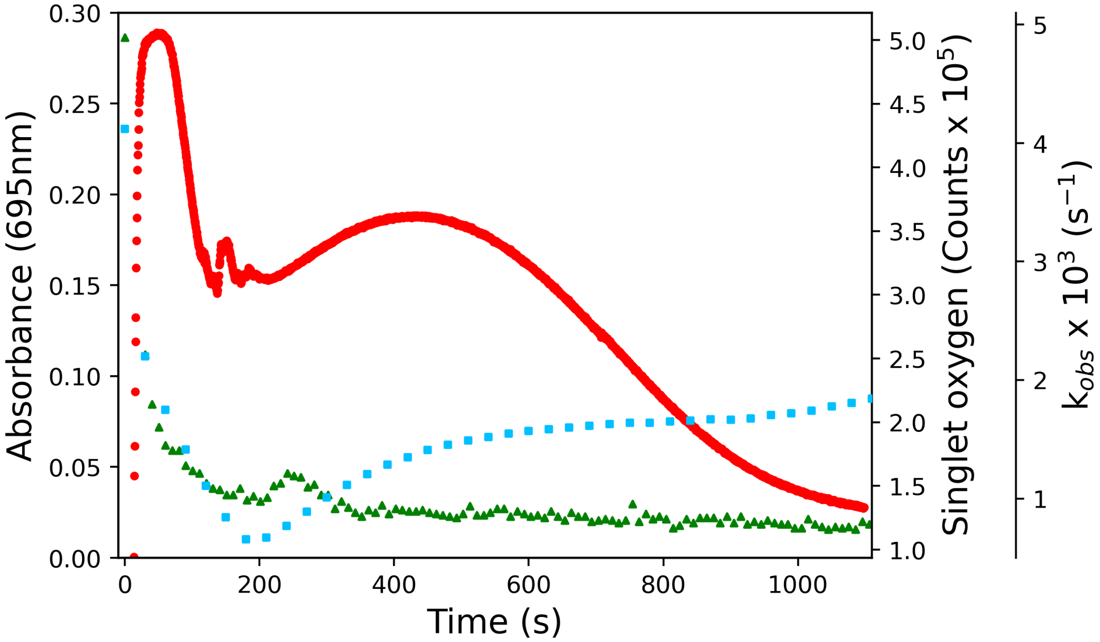

Figure S29: k_{obs} (light blue squares), intensity of $^1O_2$ emission (green triangles) and NIR absorption of **4** (red circle, see Figure 4) over 20 min following addition of PhPAA under reaction conditions described above. The observed rate of decay of emission (k_{obs}) from $[Ru(ph_2phen)_3]^{2+}$ is plotted as it is linearly related to $[O_2]$.

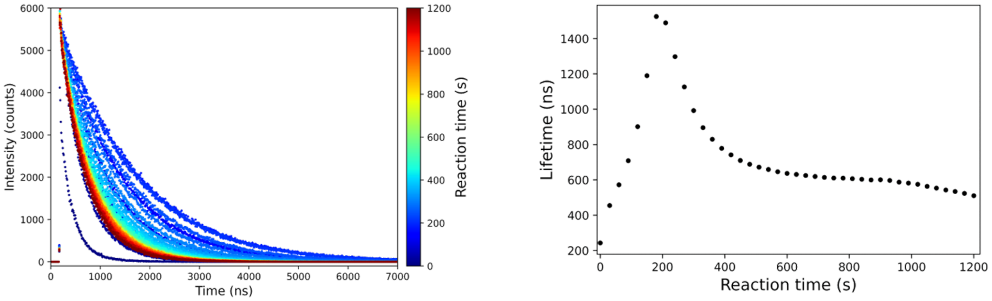

Figure S30: Changes in emission decay lifetime of $[Ru(ph_2phen)_3]^{2+}$ under standard reaction conditions over 20 min after the addition of PhPAA: (left) Emission decay of $[Ru(ph_2phen)_3]^{2+}$, (right) Emission decay lifetime of $[Ru(ph_2phen)_3]^{2+}$ over the course of the reaction.

1 with 100 equiv. phenylperacetic acid in TFE

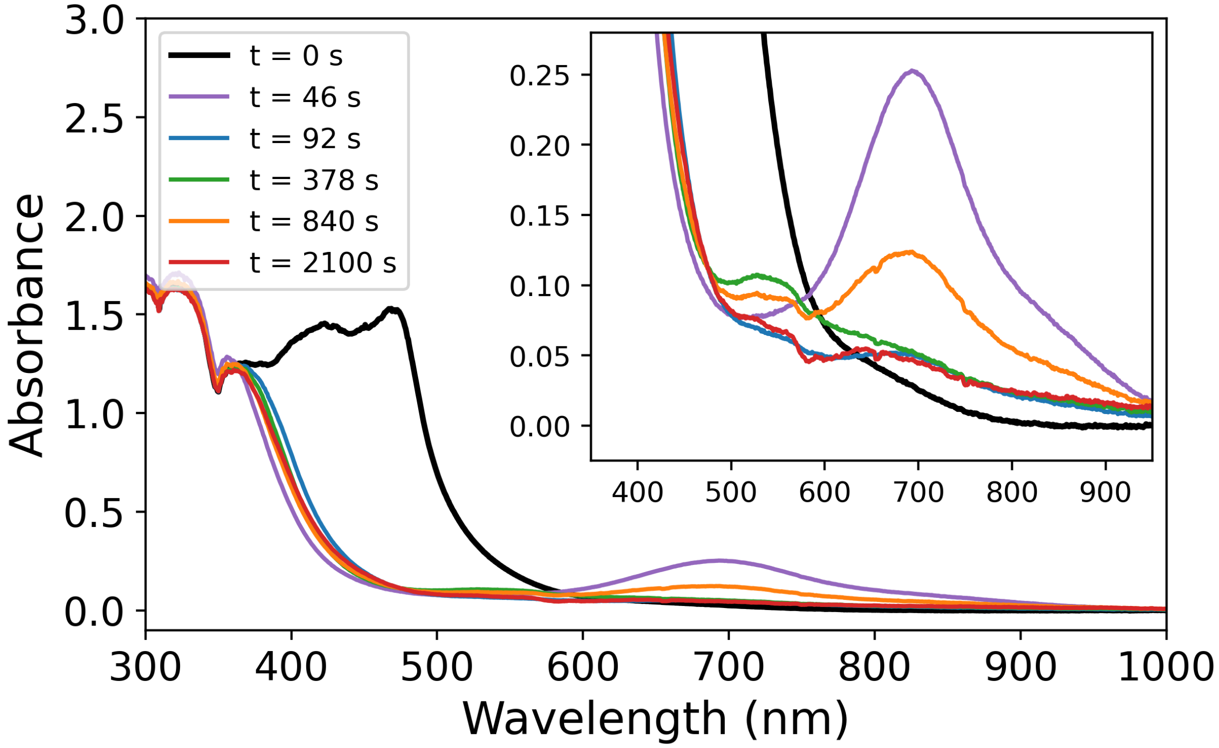

Figure S31: UV/vis absorption spectra following addition of 100 equiv. PhPAA to **1** in TFE. Conditions: 1 mM **1**, 100 mM phenylperacetic acid, in 1.5 mL TFE.

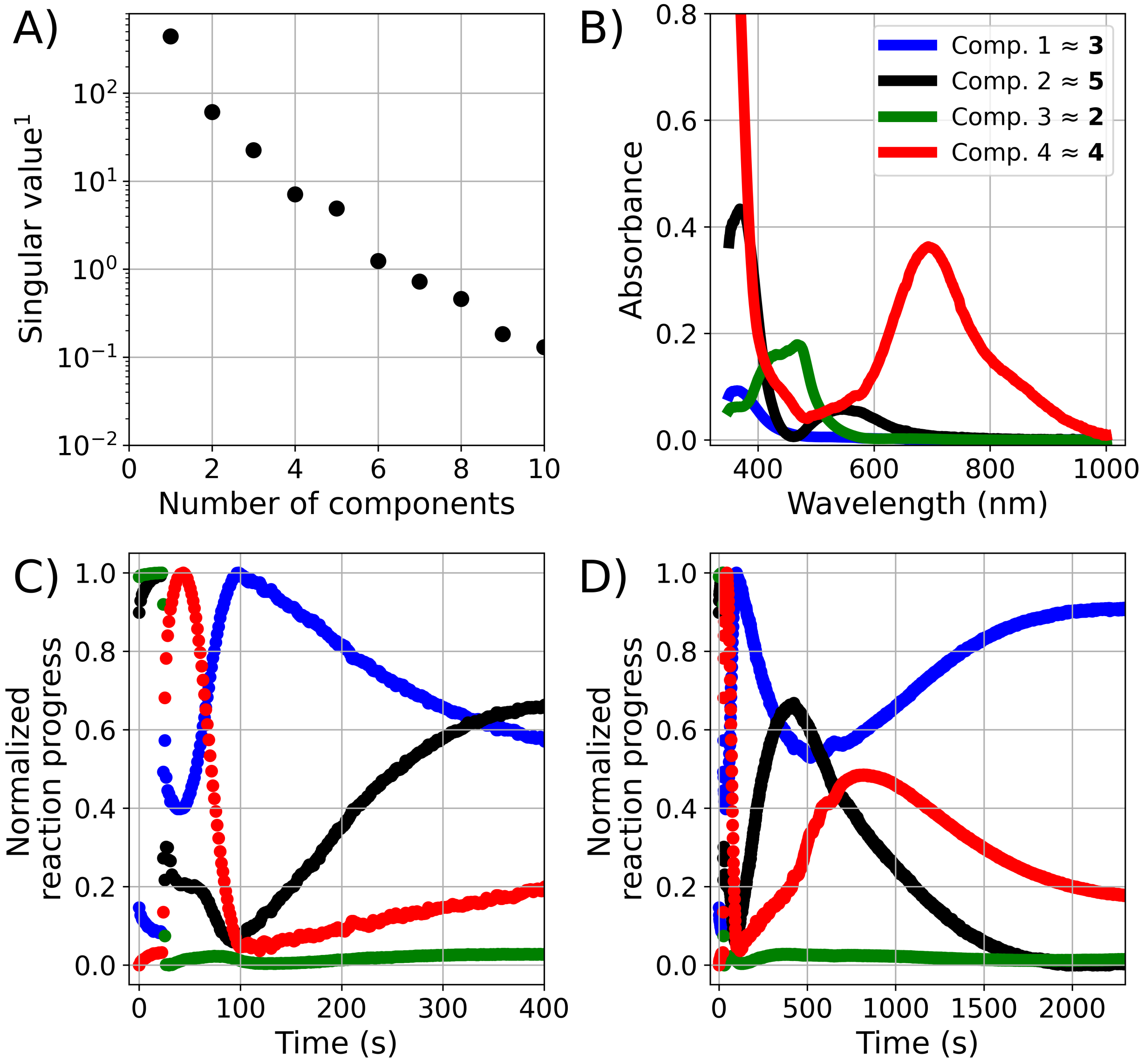

Figure S32: MCR analysis of UV/vis absorption spectra obtained following addition of 100 equiv. PhPAA to **1** in TFE using four components to resolve the dataset. A) Number of components that the singular value decomposition (SVD) uses to resolve the dataset.[1] The singular value is related to the importance of this component to resolve the dataset; the smaller the singular value, the less important is the corresponding component to resolve the dataset. B) The four component spectra ('shapes') with their relative importance: Comp. 1 (**3**, blue), Comp. 2 (**5**, black), Comp. 3 (**2**, green), and Comp. 4 (**4**, red). C) and D) The relative importance (contribution) of each component at each data point over 400 s and 2300 s, respectively. It should be noted that component concentrations obtained from MCR only represent absorbance contributions over time and are normalized in each case, and thus do not present the actual concentrations of the various iron species present.

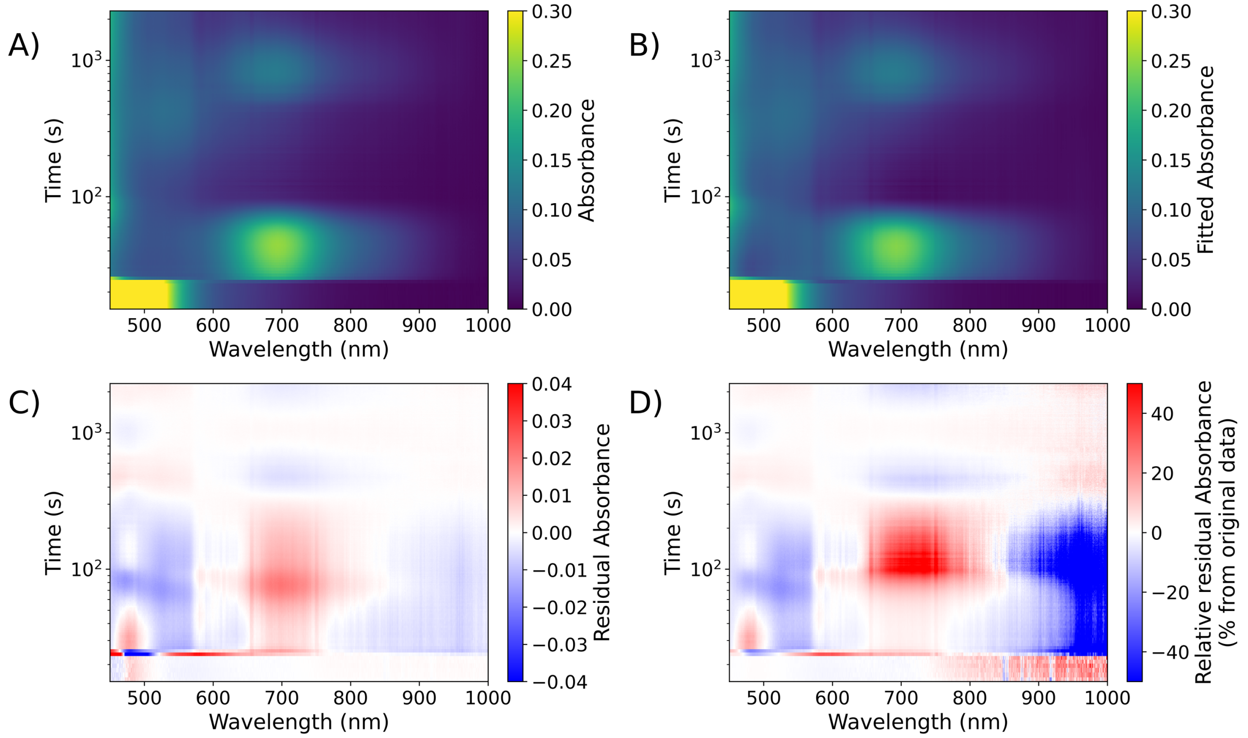

Figure S33: Multivariate curve resolution (MCR) of the UV/vis absorption spectra of **1** (1 mM) in TFE following addition of 100 eq. PhPAA using four components to resolve the data. A) and B) depict the representation of the original data and fitted data, respectively. C) and D) show the residual absorbance (actual absorbance minus fitted absorbance) and the residual absorbance as percentage of the actual absorbance, respectively. It is of note that in Figures A) and B) the absorbance is graphed between 0-0.3 to show features relevant to the iron complexes with good contrast. However, as a result the **2** that is present initially ($t < 8$ s) is misrepresented as having an absorbance of 0.3, instead of ca. 15

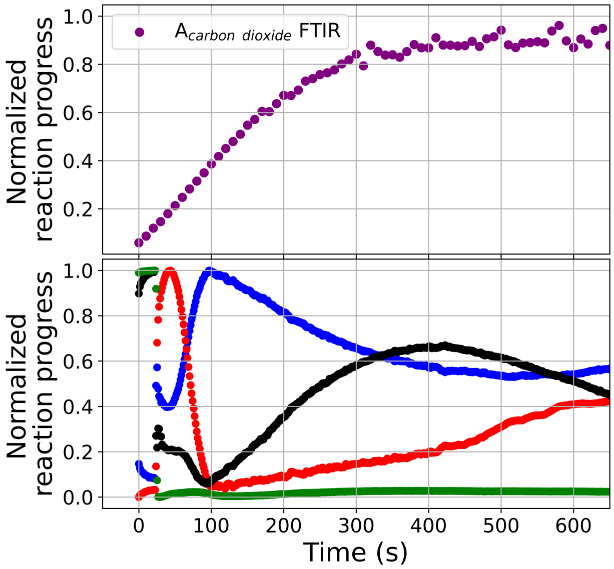

Figure S34: Reaction progress obtained from MCR analysis of UV/vis absorption and FTIR spectra with 100 equiv. PhPAA and 1 mM **1** in TFE. (top) The reaction progress of CO_2 over time (FTIR spectra shown in figure S35). (bottom) MCR traces from UV/vis absorption spectra of Fe(III) species (blue), **5** (black), Fe(II) species (green), and **4** (red). Conditions: 1 mM **1**, 100 mM PhPAA, in 1.5 mL TFE.

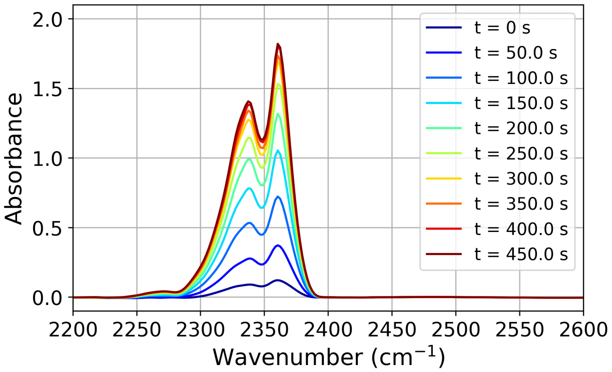

Figure S35: FTIR spectra (headspace) over time during the reaction of 100 equiv. phenylperacetic acid with **1** (1 mM) in TFE.

1 with 100 equiv. phenylperacetic acid under Ar

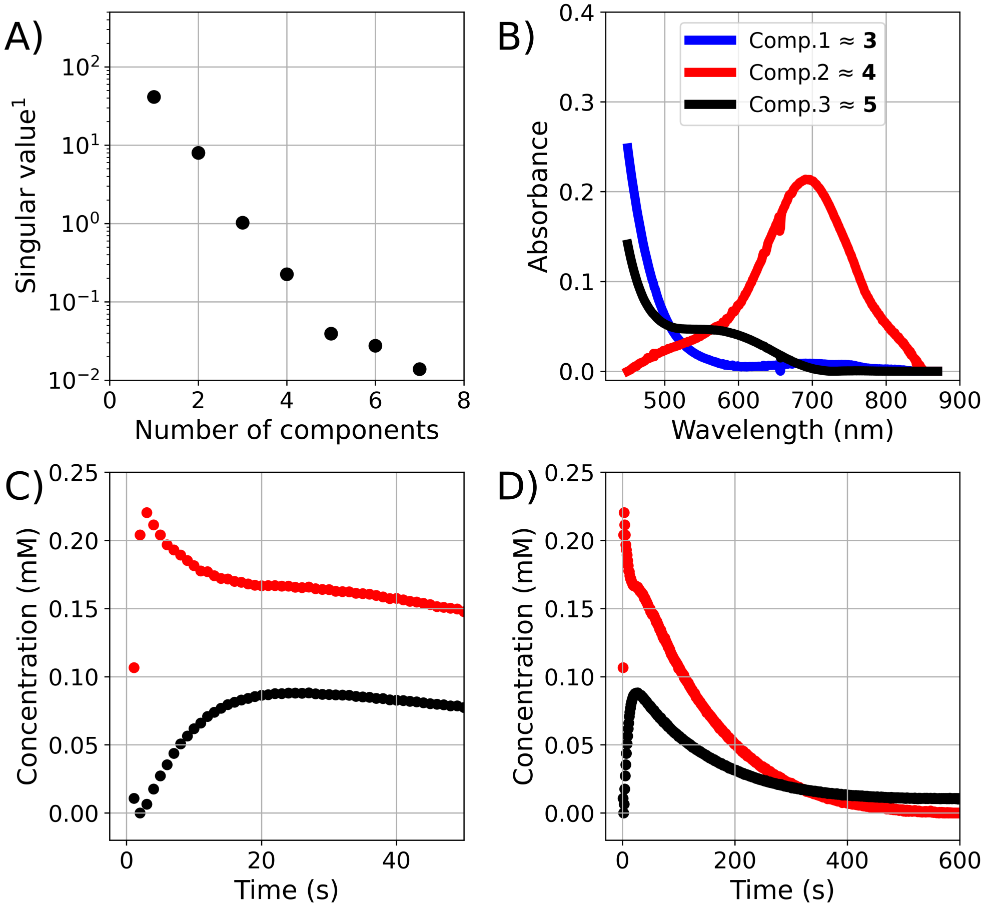

Figure S36: Results from MCR analysis of UV/vis absorption spectra during the reaction of 100 equiv. PhPAA with **1** in methanol under argon using three components to resolve the dataset. A) Number of components that the singular value decomposition (SVD) uses to resolve the dataset.[1] The singular value is related to the importance of this component to resolve the dataset; the smaller the singular value, the less important is the corresponding component to resolve the dataset. B) The three component spectra ('shapes') with their relative importance: **3** (blue), **4** (red), and **5** (black). C) and D) The relative importance (contribution) of comp. 2 and comp. 3 (**4** and **5**, respectively) at each data point over 50 s and 600 s, respectively.

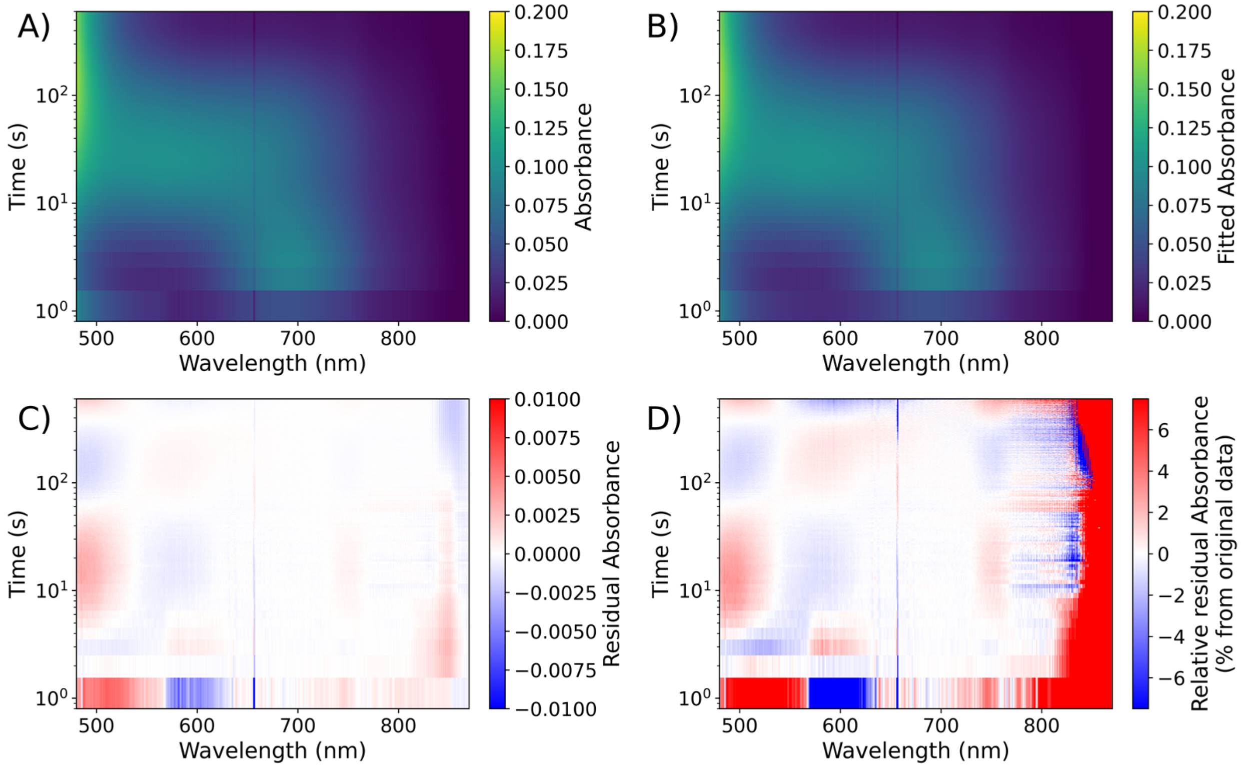

Figure S37: Multivariate curve resolution (MCR) analysis of UV/vis absorption spectra during the reaction of 1 mM **1** with 100 equiv. PhPAA in methanol under argon using three components to resolve the data. A) and B) depict the representation of the original data and fitted data, respectively. C) and D) show the residual absorbance (actual absorbance minus fitted absorbance) and the residual absorbance as percentage of the actual absorbance, respectively.

1 with 100 equiv. phenylperacetic acid under $^{18}O_2$

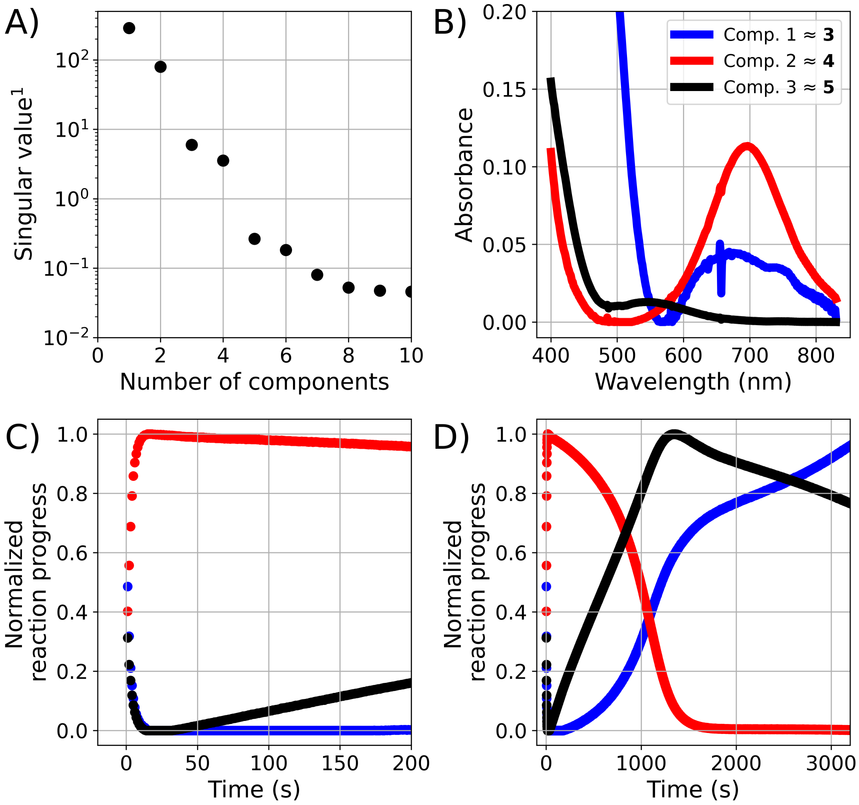

Figure S38: Multivariate curve resolution (MCR) analysis of UV/vis absorption spectra during the reaction of 1 mM **1** with 100 equiv. PhPAA in methanol under $^{18}O_2$ using three components to resolve the data. A) Number of components that the singular value decomposition (SVD) uses to resolve the dataset.[1] The singular value is related to the importance of this component to resolve the dataset; the smaller the singular value, the less important is the corresponding component to resolve the dataset. B) The three component spectra ('shapes') with their relative importance: **3** (blue), **4** (red), and **5** (black). C) and D) The relative importance (contribution) of each component at each data point over 200 s and 3200 s, respectively. It should be noted that component concentrations obtained from MCR only represent absorbance contributions over time and are normalized in each case, and thus do not present the actual concentrations of the various iron species present.

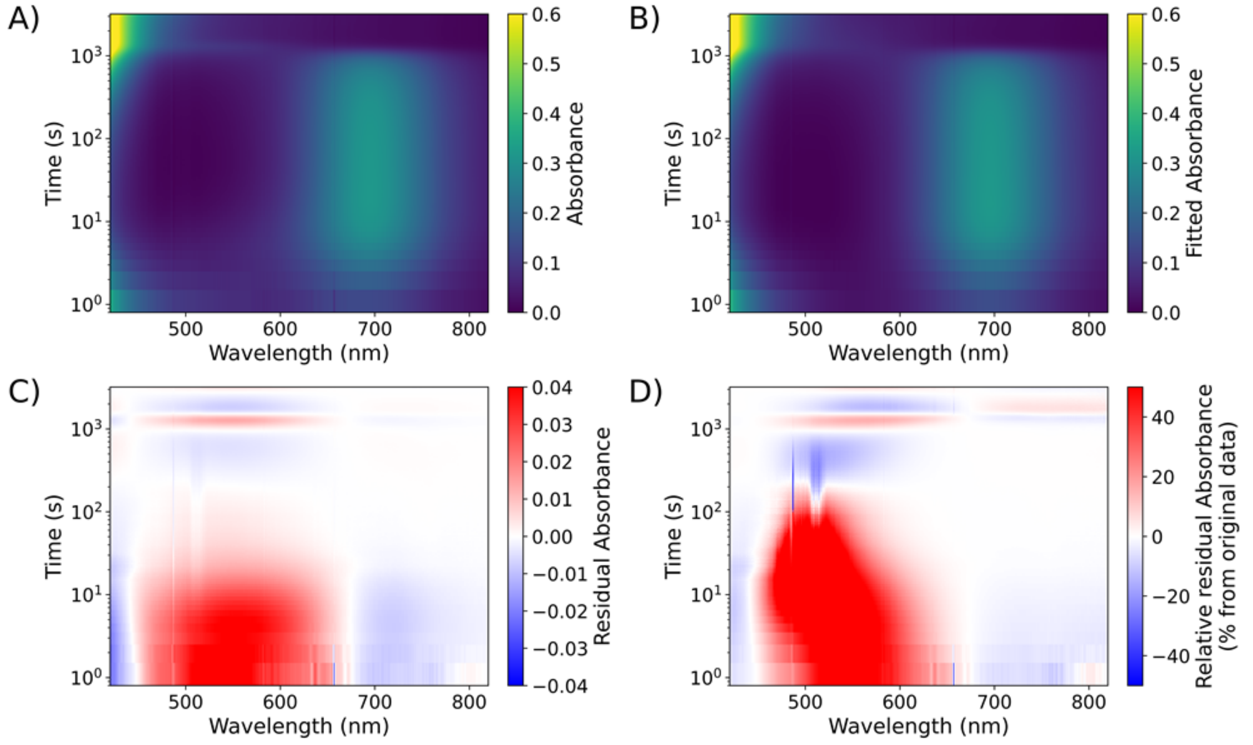

Figure S39: Multivariate curve resolution (MCR) analysis of UV/vis absorption spectra during the reaction of 1 mM **1** with 100 equiv. PhPAA in methanol under $^{18}O_2$ using three components to resolve the data. A) and B) depict the representation of the original data and fitted data, respectively. C) and D) show the residual absorbance (actual absorbance minus fitted absorbance) and the residual absorbance as percentage of the actual absorbance, respectively.

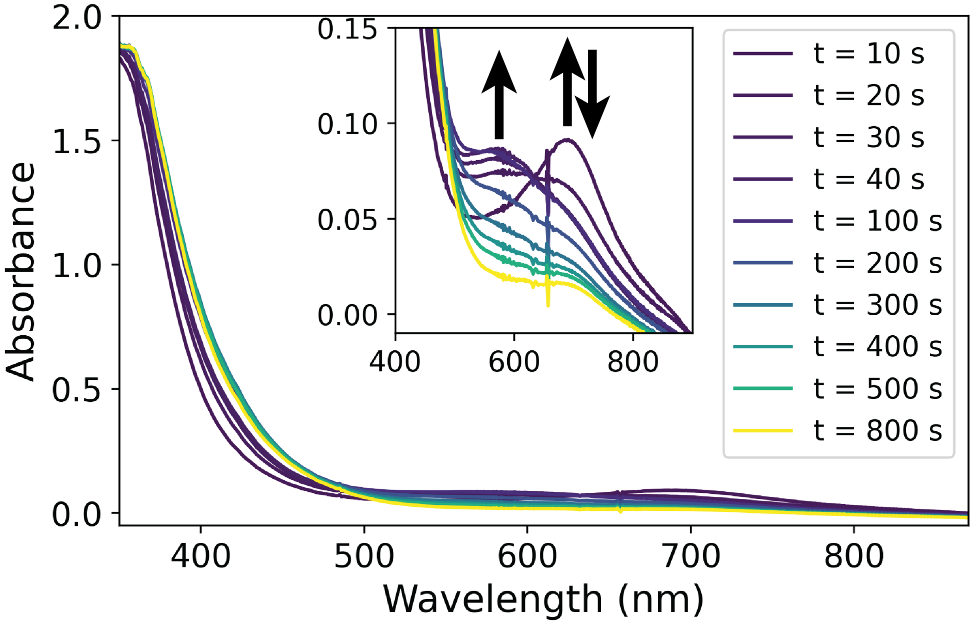

Figure S40: UV/vis absorption spectra of **1** over the 800 s following addition of PhPAA. Inset: expansion of NIR region of the spectrum. Conditions: 1 mM **1**, 100 mM phenylperacetic acid in 1.5 mL CD_3OD under argon.

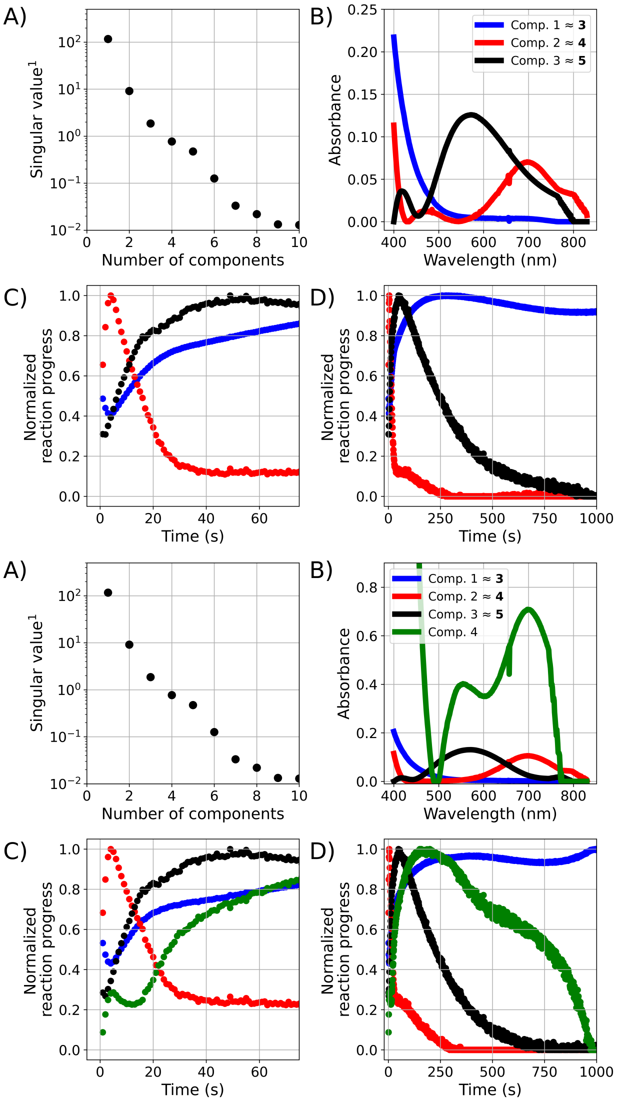

Figure S41: Multivariate curve resolution (MCR) analysis of the UV/vis absorption spectra recorded during the reaction of **1** (1 mM) with 100 eq. PhPAA in CD_3OD under argon. Using (right) three and (left) four components to resolve the data. A) Number of components that the singular value decomposition (SVD) uses to resolve the dataset.[1] The singular value is related to the importance of this component to resolve the dataset; the smaller the singular value, the less important is the corresponding component to resolve the dataset. B) The three component spectra ('shapes') with their relative importance. The spectra are assigned to **3** (blue), **4** (red), and **5** (black) based on resemblance to real spectra. Component 4 (green) is essentially a combination of the absorbance of **4** (red), and **5** (black) C) and The relative importance (contribution) of each component at each data point over (C) 75 s and (D) 1000 s. It should be noted that component concentrations obtained from MCR only represent absorbance contributions over time and are normalized in each case, and thus do not present the actual concentrations of the various iron species present.

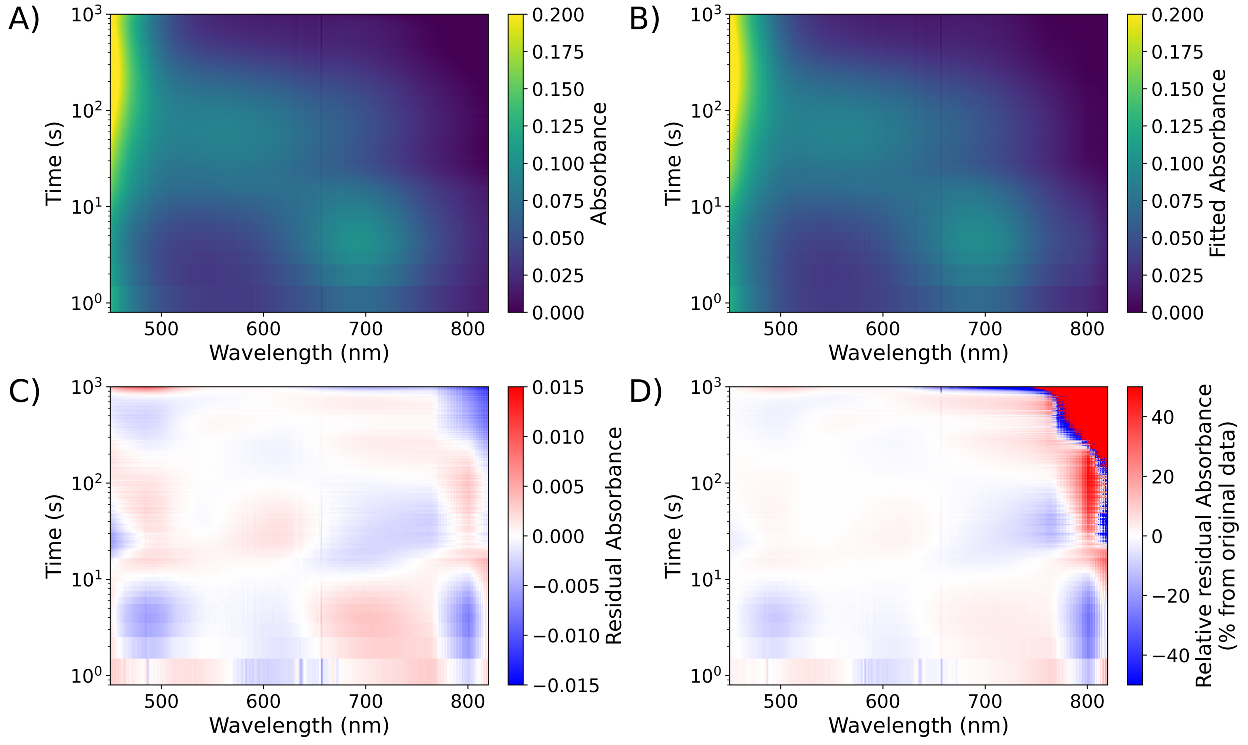

Figure S42: Multivariate curve resolution (MCR) analysis of UV/vis absorption spectra during the reaction of 1 mM **1** with 100 eq. PhPAA in CD_3OD under argon using three components to resolve the data. A) and B) depict the representation of the original data and fitted data, respectively. C) and D) show the residual absorbance (actual absorbance minus fitted absorbance) and the residual absorbance as percentage of the actual absorbance, respectively.

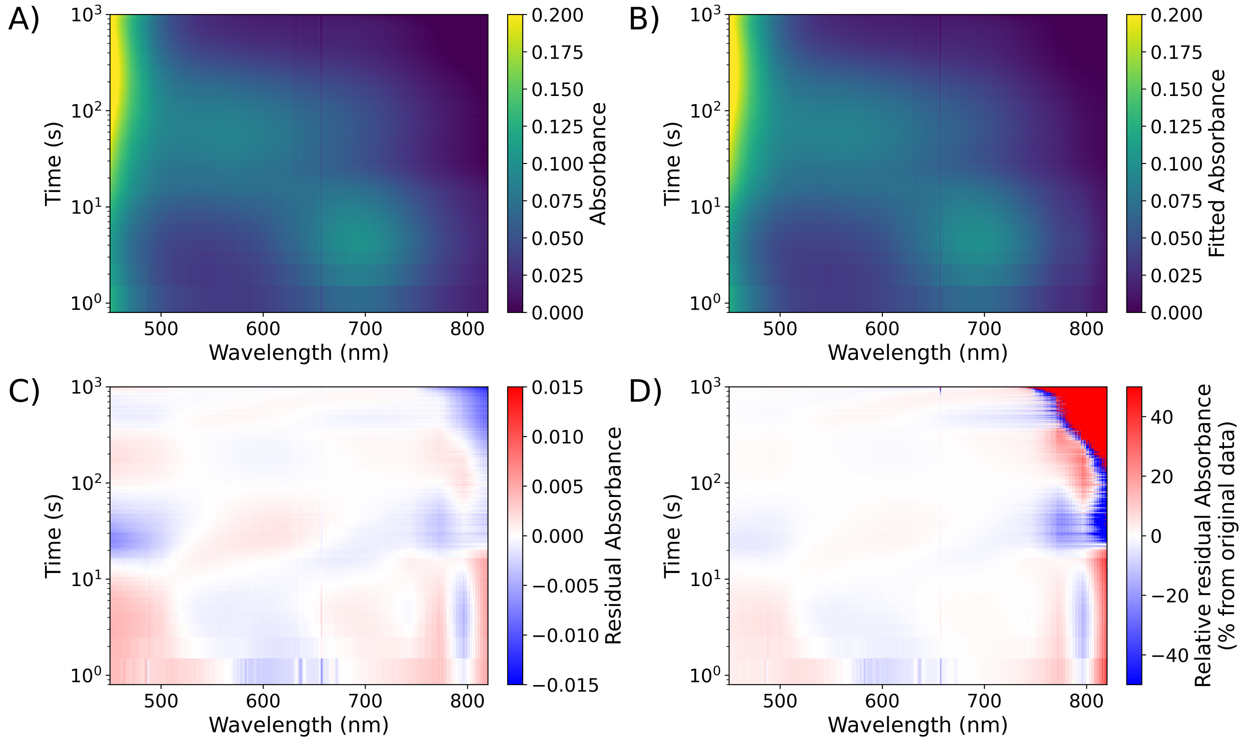

Figure S43: Multivariate curve resolution (MCR) analysis of UV/vis absorption spectra during the reaction of 1 mM **1** with 100 eq. PhPAA in CD_3OD under argon using four components to resolve the data. A) and B) depict the representation of the original data and fitted data, respectively. C) and D) show the residual absorbance (actual absorbance minus fitted absorbance) and the residual absorbance as percentage of the actual absorbance, respectively.

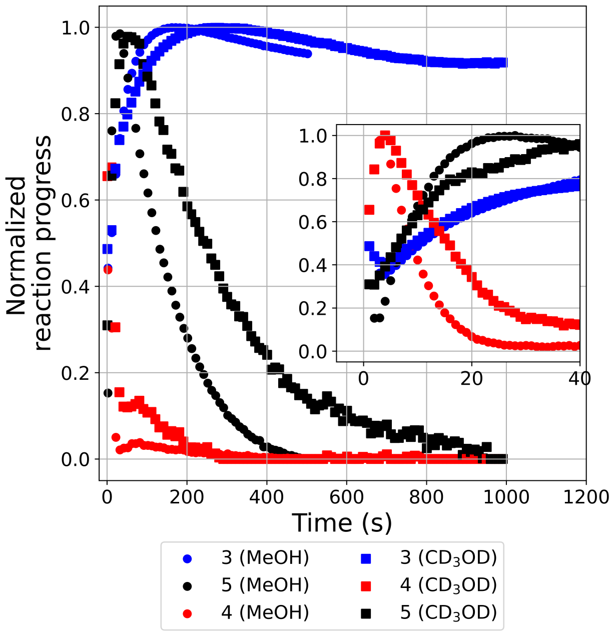

Figure S44: Time traces between 0-1000 s (inset 0-40 s) from the MCR analysis of UV/vis absorption spectra during the reaction of PhPAA with **1** under argon in CH_3OH compared to in CD_3OD. Fe(III) species (blue), **4** (red), and **5** (black). Circles represent the time traces from CH_3OH and rectangles from CD_3OD.

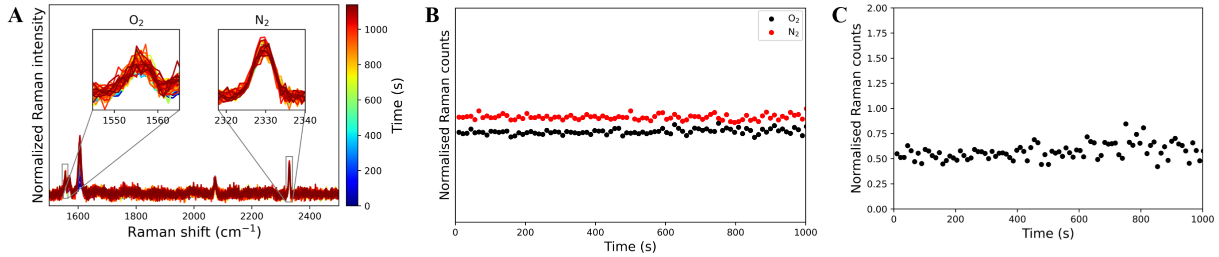

Figure S45: Raman headspace analysis (532 nm) of the reaction of 100 equiv. phenylperacetic acid with **1** under an Argon atmosphere. A) Raman spectra over time (inserts: characteristic O_2 and N_2 bands). B) Progression vs time of O_2 and N_2 for comparison. C) Ratio of O_2/N_2 bands over time for comparison. As expected, a change in the Raman band of O_2 is not observed since it originates from air outside of the cuvette. Conditions: 1 mM **1**, 100 mM PhPAA in 1.5 mL MeOH.

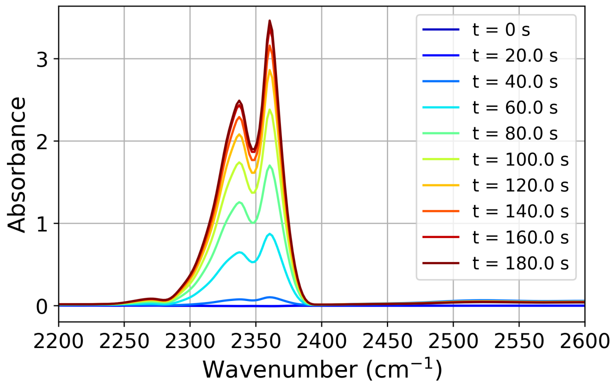

Figure S46: FTIR spectra (headspace) of the reaction of 100 equiv. phenylperacetic acid with **1** (1 mM) in methanol under an Argon atmosphere.

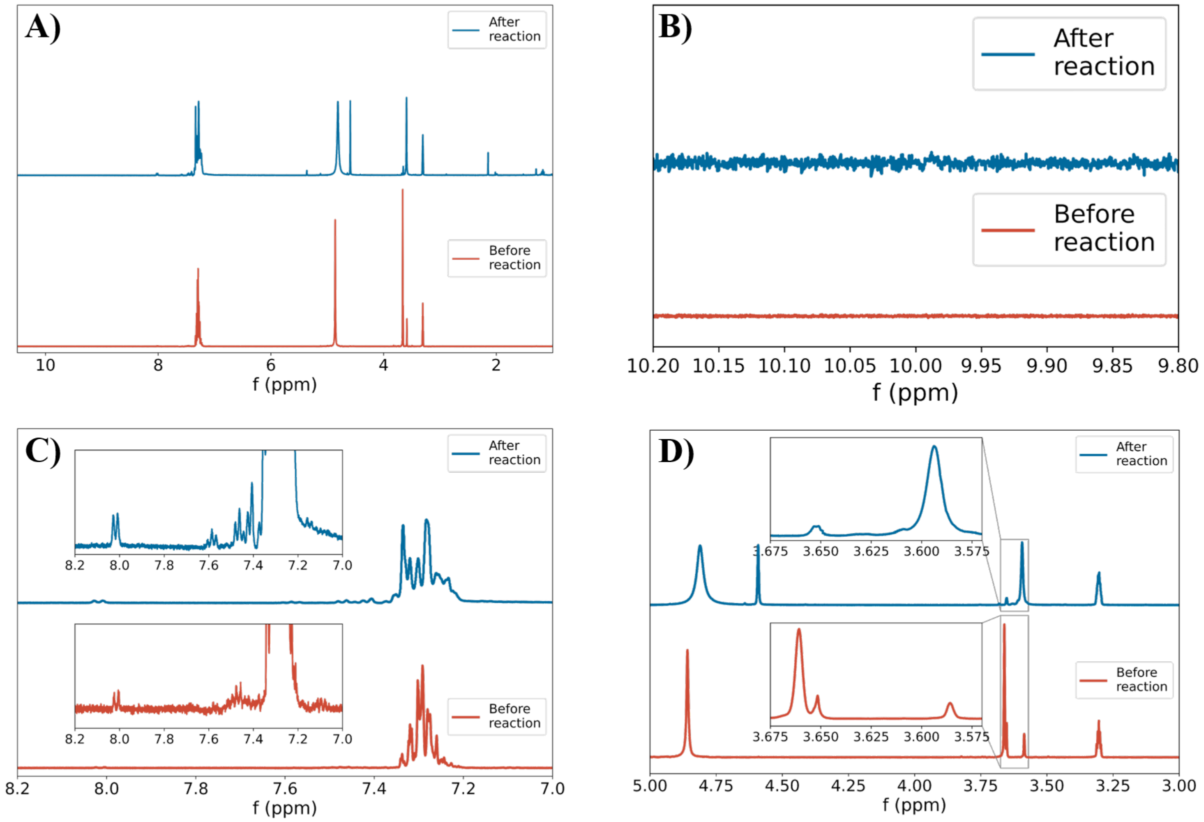

Figure S47: ^{1}H-NMR spectra of the reaction of 100 equiv. phenylperacetic acid with **1** under an Argon atmosphere. A), B), C) and D) show the ^{1}H-NMR before (red) and 2 h after addition of phenylperacetic acid (blue) in different ppm ranges. Conditions: 1 mM **1**, 100 mM PhPAA in 1.5 mL CD_3OD.

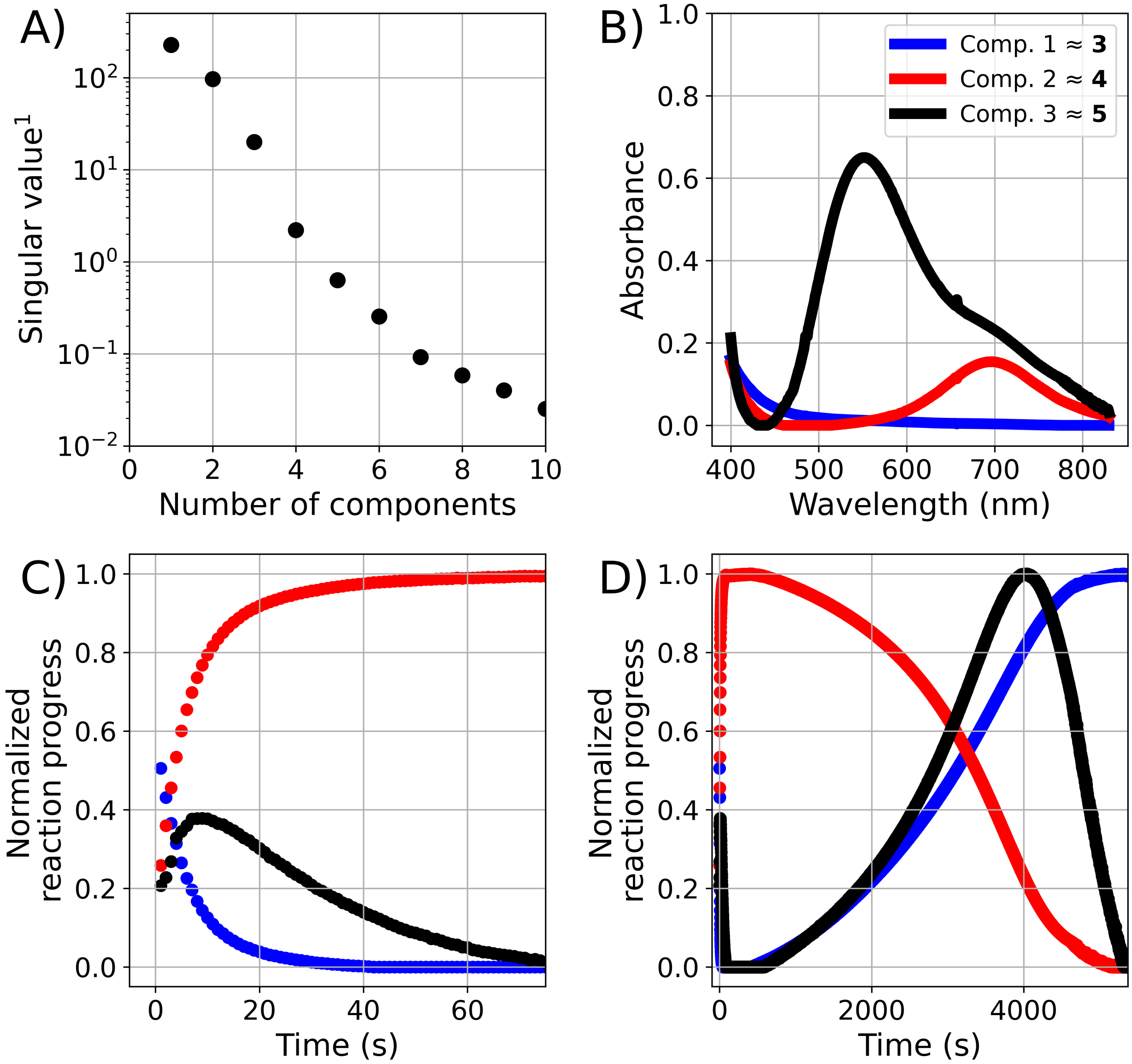

Figure S48: Multivariate curve resolution (MCR) analysis of UV/vis absorption spectra during the reaction of 1 mM **1** with 100 eq. PhPAA in CD_3OD under $^{18}O_2$ using three components to resolve the data. A) Number of components that the singular value decomposition (SVD) uses to resolve the dataset.[1] The singular value is related to the importance of this component to resolve the dataset; the smaller the singular value, the less important is the corresponding component to resolve the dataset. B) The three component spectra ('shapes') with their relative importance: **3** (blue), **4** (red), and **5** (black). C) and D) The relative importance (contribution) of each component at each data point over 75 s and 5400 s, respectively. It should be noted that component concentrations obtained from MCR only represent absorbance contributions over time and are normalized in each case, and thus do not present the actual concentrations of the various iron species present.

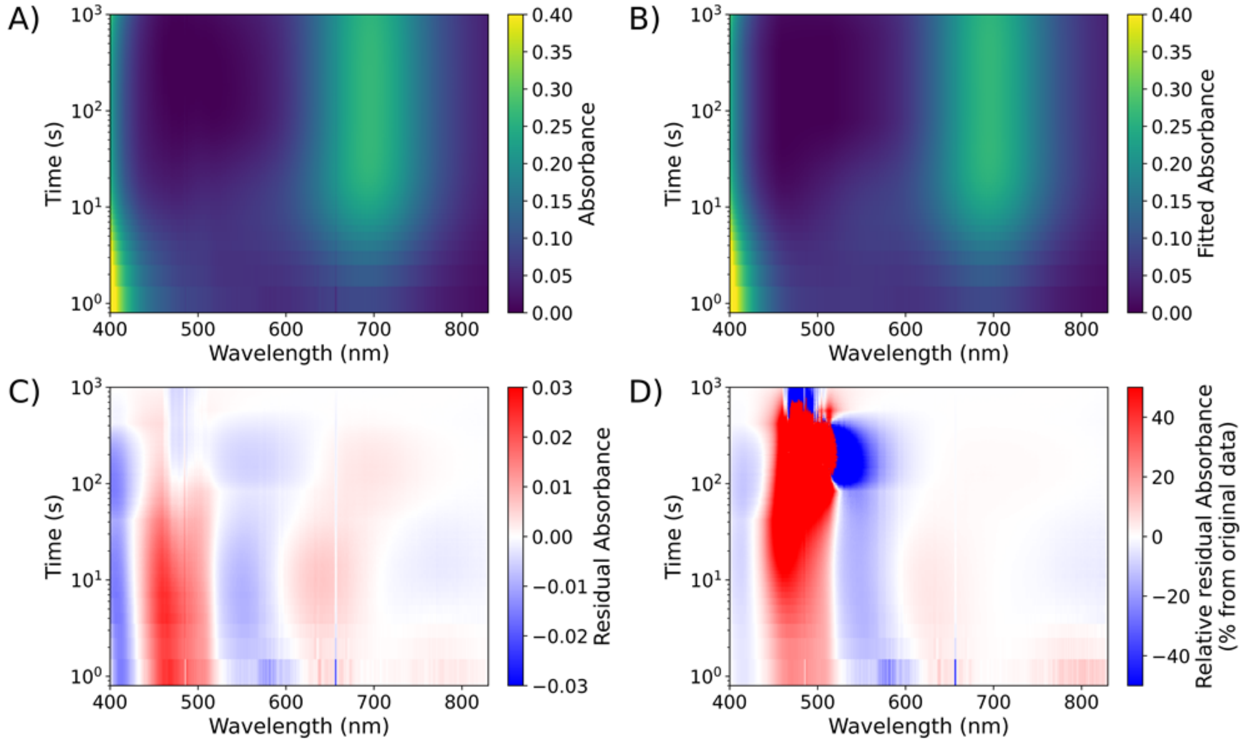

Figure S49: Multivariate curve resolution (MCR) analysis of UV/vis absorption spectra during the reaction of 1 mM **1** with 100 eq. PhPAA in CD_3OD under $^{18}O_2$ using three components to resolve the data. A) and B) depict the representation of the original data and fitted data, respectively. C) and D) show the residual absorbance (actual absorbance minus fitted absorbance) and the residual absorbance as percentage of the actual absorbance, respectively.

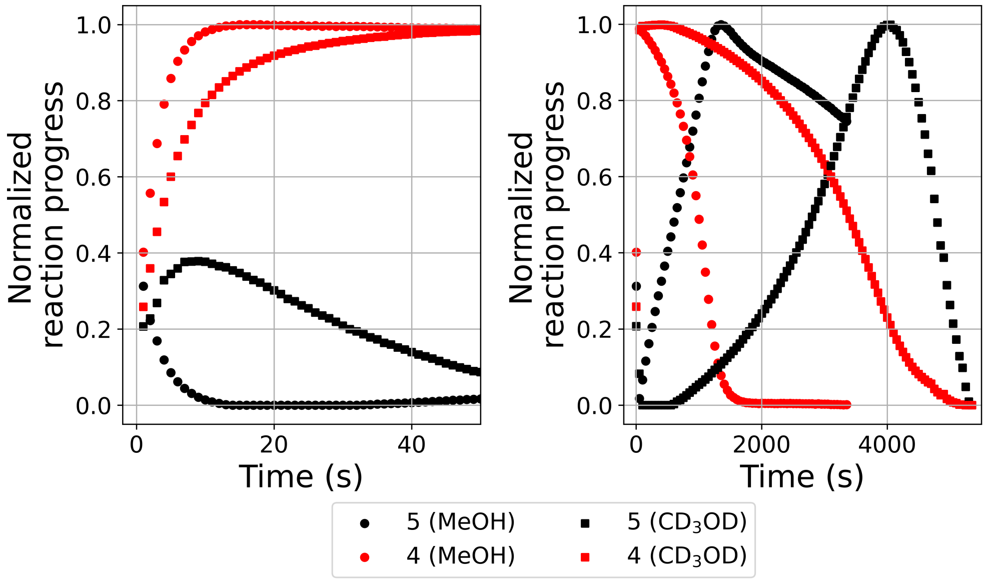

Figure S50: Time traces (left: 0-50 s, right: 0-5000 s) from the MCR analysis of UV/vis absorption spectra during the reaction of PhPAA to **1** under $^{18}O_2$ in CH_3OH vs in CD_3OD. **4** (red) and **5** (black). Circles represent the time traces from CH_3OH and rectangles from CD_3OD.

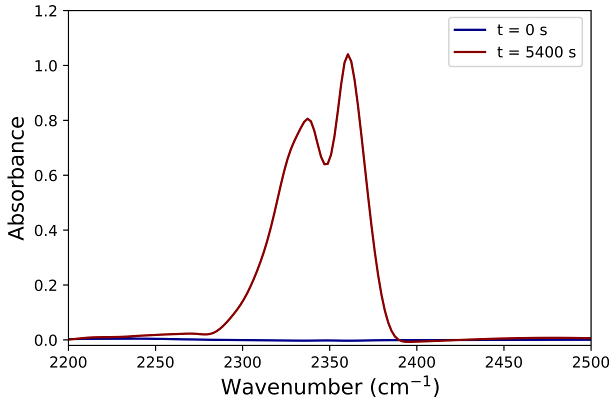

Figure S51: FTIR spectra (headspace) before and after the reaction of 100 equiv. phenylperacetic acid with **1** (1 mM) in methanol under $^{18}O_2$ atmosphere.

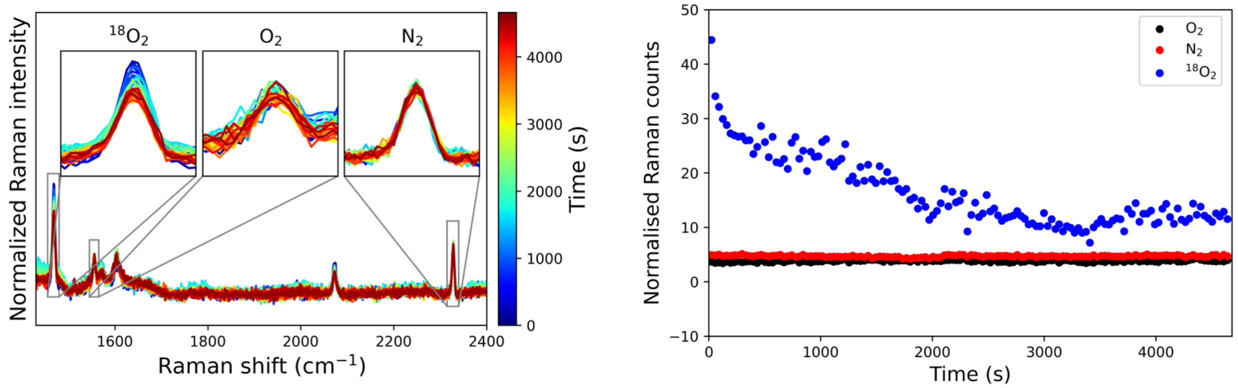

Figure S52: Raman headspace analysis (532 nm) of the reaction of 100 equiv. phenylperacetic acid with **1** under $^{18}O_2$ atmosphere. Left: Raman spectra over time (inserts: characteristic $^{18}O_2$, O_2, and N_2 bands). Right: Progression vs time of $^{18}O_2$, O_2, and N_2 for comparison. As expected, a change in the Raman band of $^{16}O_2$ is not observed since it originates from air outside of the cuvette. Conditions: 1 mM **1**, 100 mM PhPAA in 1.5 mL MeOH.

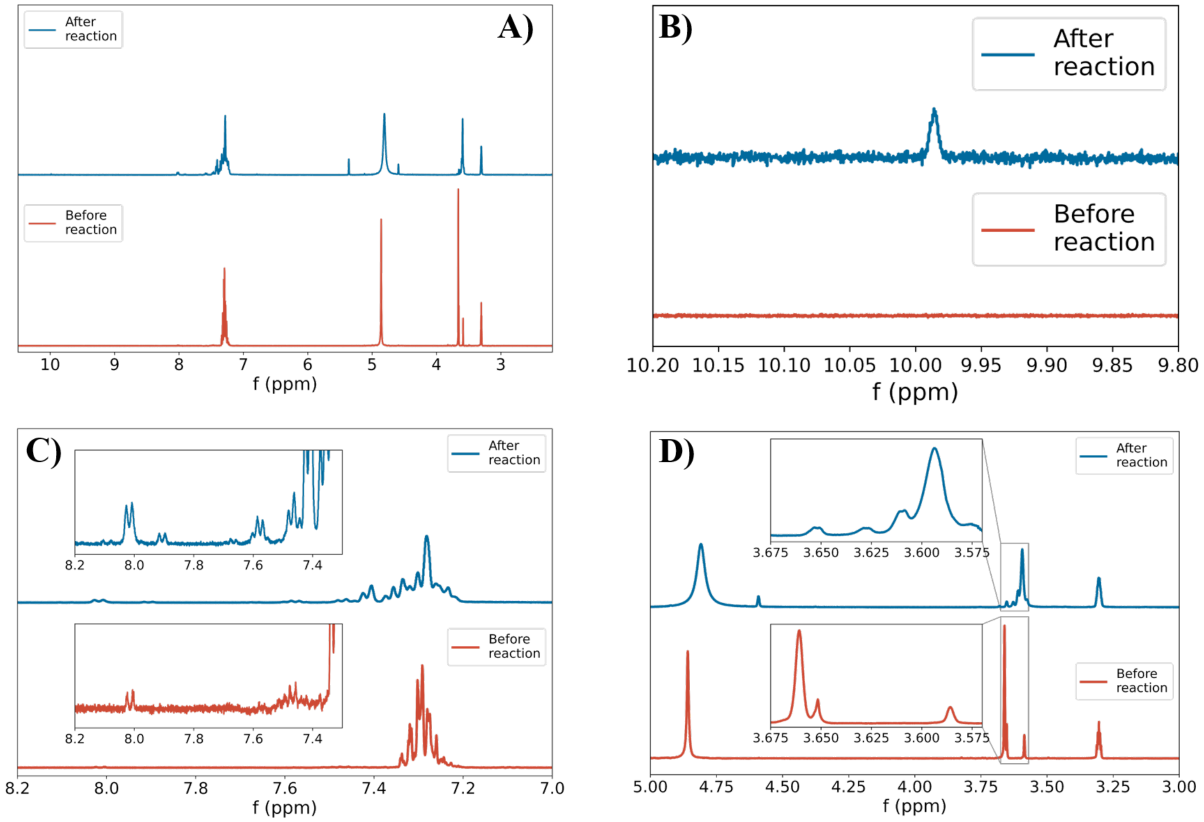

Figure S53: ^{1}H-NMR of the reaction of 100 equiv. phenylperacetic acid with **1** under $^{18}O_2$ atmosphere. A), B), C) and D) show the ^{1}H-NMR before (red) and 2h after addition of phenylperacetic acid (blue) at different ppm domains. Conditions: 1 mM **1**, 100 mM phenylperacetic acid in 1.5 mL CD_3OD.

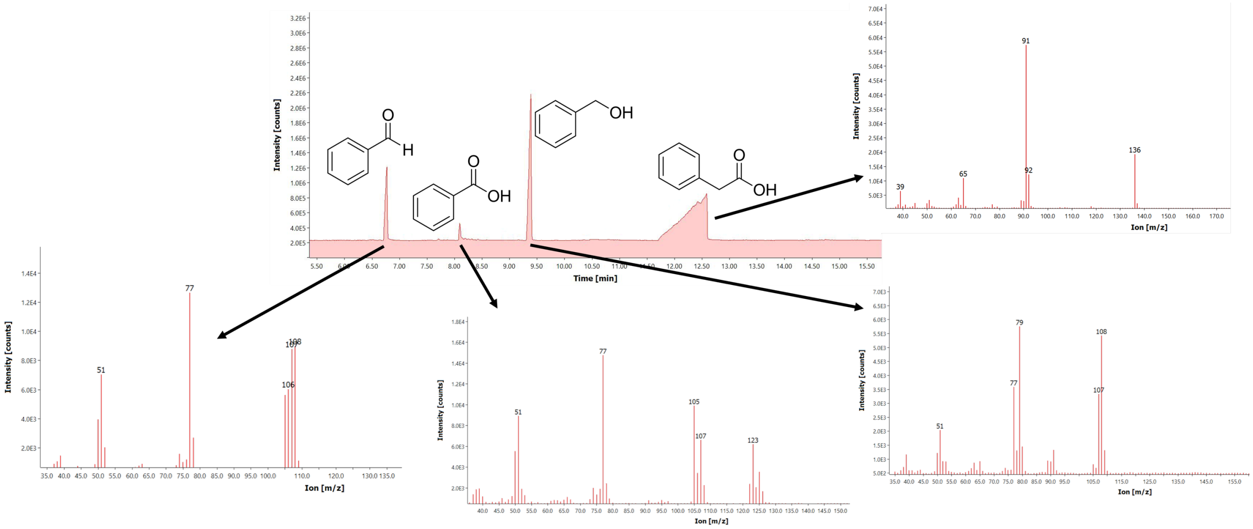

Figure S54: GC-MS chromatogram of the reaction mixture after 2 hours of reaction of 100 equiv. phenylperacetic acid with **1** under $^{18}O_2$ atmosphere. For each peak the MS spectrum is shown, from which the labeled products, i.e., benzaldehyde and benzoic acid, are clearly visible. Conditions: 1 mM **1**, 100 mM phenylperacetic acid in 1.5 mL CD_3OD.

4 and 3a with 100 equivalent phenylperacetic acid in methanol

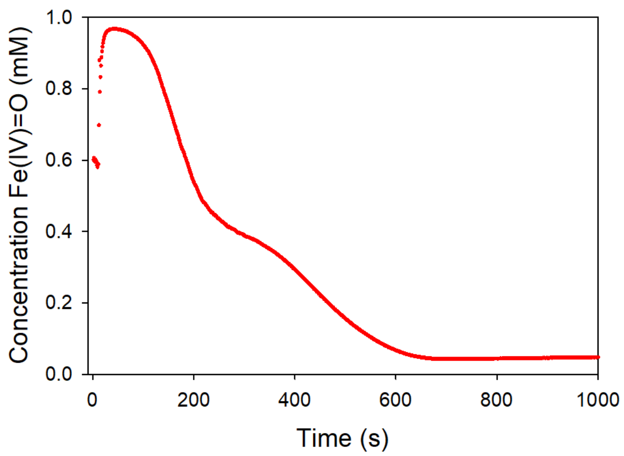

Figure S55: Concentration of **4** over time after the addition of phenylperacetic acid (100 mM) to pre-prepared **4**. Conditions: 1 mM **4**, 100 mM PhPAA in 1.5 mL MeOH.

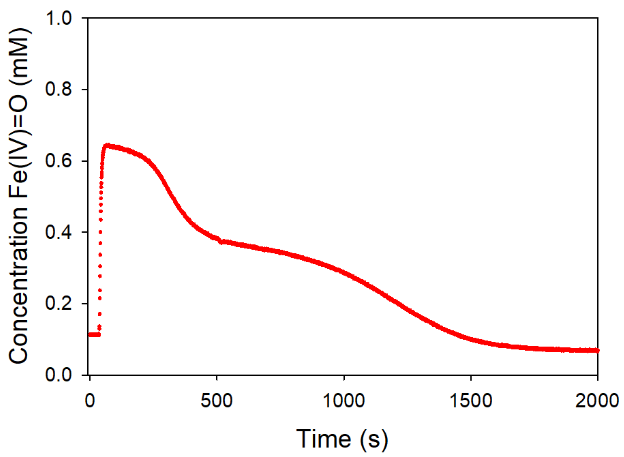

Figure S56: Concentration of **4** over time after the addition of phenylperacetic acid (100 mM) to the Fe(III) complex. Conditions: 1 mM **3a**, 100 mM PhPAA in 1.5 mL MeOH.

Kinetic modeling without consideration of methanol oxidation

The parameters of the kinetic model constructed for the reaction of **1** with a large excess of PhPAA under argon (Figure S57) were adjusted to match to the experimental data (Raman, and UV/vis and FTIR absorption spectroscopy). Figures S57A and S57B show the reaction progress of **2**, **3**, **4**, and **5**, and of PhPAA, phenylacetic acid (PhAA), CO_2, benzyl radical, and benzyl alcohol, respectively. The model accounts fully for the benzyl alcohol formed and CO_2 evolved over time. Since both components originate from the same reaction (k_3) only benzyl alcohol is shown in Figure S57B. However, the model does not explain well reaction progress in regard to iron species and other products (figures S57C, D, E, F).

Comparison of the outcome of MCR analysis of UV/vis absorption spectra to the modeled reaction progress of **4** and **5** (Figure S57C) reveals a similar trajectory for the two components, however, the actual reaction is faster than the model predicts (Figure S57D). Moreover, the amount of **4** present initially (ca. 0.2 mM) is greatly overestimated by the kinetic model (ca. 1 mM).

In Figures S57E and F, the observed concentration of PhPAA, PhAA, benzyl alcohol and CO_2 over time is compared to that predicted by the model. PhPAA is consumed faster than predicted, and the amount of PhAA and benzyl alcohol/CO_2 are highly underestimated and overestimated by the model, respectively (S57E and F).

The model described in Scheme 3 does not predict well the observed progression of the reaction and variation in the rate constants could not yield a satisfactory fit. Important discrepancies between the modeled and experimental data are the reaction progress and the mass balance for PhPAA, PhAA, benzyl alcohol and CO_2. The underestimation of PhAA and overestimation of benzyl alcohol and CO_2 suggests that the reaction between Fe(II) and PhPAA is underrepresented in the model, i.e., during the reaction more Fe(II) must be available to react with PhPAA. This hints to the presence of an additional pathway

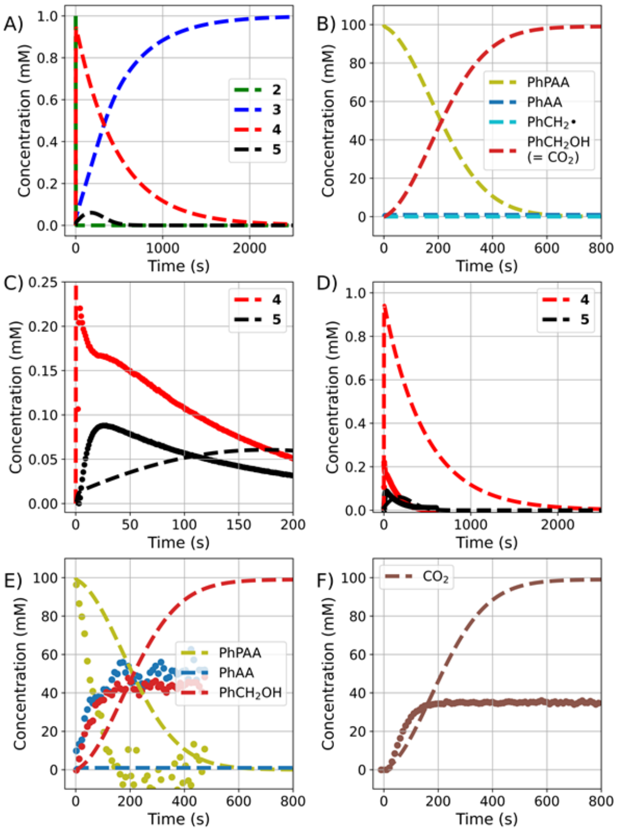

Figure S57: Microkinetic modeling of the reaction of **1** (1 mM) with 100 equiv. PhPAA under argon. A) Modeled concentrations of the iron species vs time; B) Modeled concentrations of phenylperacetic acid (PhPAA), phenylacetic acid (PhAA), CO_2, benzyl radical, and benzyl alcohol vs time; C), D) Experimental data from UV/vis and modeled concentrations of **4** and **5** over 200 s and 2500 s, respectively; E) Experimental data from liquid phase Raman spectroscopy and modeled concentrations of PhPAA, PhAA, and benzyl alcohol vs time; F) Experimental data from headspace FTIR spectroscopy and modeled concentrations of CO_2 vs time. The dashed and dotted lines represent the modeled and the experimental data, respectively. Color legend: **2** (green), **4** (red), **3** (blue), **5** (black), PhPAA (olive), PhAA (light blue), CO_2 (brown), benzyl radical (cyan), and benzyl alcohol (light red).

that forms Fe(II). Furthermore, Fe(IV)=O is much shorter-lived experimentally than in the model, Figure S57D, indicating that an additional pathway where Fe(IV)=O is reduced to Fe(II) is involved. Therefore, the proposed mechanism must be extended.

Methanol is the most plausible species available to reduce Fe(IV)=O to Fe(II), however the reaction of methanol with **4** is too slow to be kinetically competent, especially in CD_3OD (see Chen et al, Chem. Commun., 2017, 53, 12357-12360). Nevertheless, the amount of the oxidation formaldehyde formed was significant (ca. 42 mM under air; ca. 37 mM under

argon) and its formation was not impacted by solvent deuteration (ca. 40 mM under argon in CD_3OD). The formation of formaldehyde also rationalizes the slightly faster decay of **4** at the end of the reaction than what would be expected for its reaction with methanol alone (Figure S13). Indeed in methanol containing 10 mM formaldehyde, **4** prepared independently decays as rapidly as observed in the present study with PhPAA.

Python scripts multivariate curve resolution (MCR) and kinetic modeling

```python
# Import packages
import sys
import logging
from pymcr.constraints import ConstraintNonneg, ConstraintNorm
from pymcr.mcr import McrAR
from pymcr.regressors import NNLS

# Apply singular value decomposition as an initial guess for MCR.
u, sing, a = np.linalg.svd(dat) # apply the SVD

"""
This cell starts the MCR and takes parameters:
N_comp: number of components you want to used for fitting
u_vectors, a_vectores: The guesses made by the cell above
# NNLS (non negative least squares) is used for MCR.
# Hence, neither spectra nor concentrations can be negative (which makes sense for UV/vis).
"""
mcrar = McrAR(c_regr=NNLS(), st_regr=NNLS(), c_constraints=[], st_constraints=[])
def start_MCR(N_comp, u_vectors, a_vectors):

    logger = logging.getLogger('pymcr')
    logger.setLevel(logging.DEBUG)
    stdout_handler = logging.StreamHandler(stream=sys.stdout)
    stdout_format = logging.Formatter('%(message)s')  # Just a basic message akin to print statements
    stdout_handler.setFormatter(stdout_format)
    logger.addHandler(stdout_handler)
    mcrar.tol_increase = 50
    mcrar.tol_n_increase = 5000
    mcrar.tol_n_above_min = 5000
    mcrar.max_iter = 10000
    mcrar.tol_err_change  = 1E-12

    guess_spectra = []

    for i in range(N_comp):
        u_guess = u.T[i]
        a_complement = a[i]

        # This is done because the actual values of spectra and their
        # complements don't matter, rather their absolute values do.
        # If a spectrum is 'inversed' (the sum of all points is negative)
        # its importance can still be represented accurately by having a
        # negative complement.
        # While this works mathematically it does not make physical sense,
        # so we inverse them.
        if np.sum(u_guess) < 0:
            if np.sum(a_complement) < 0:
                u_guess = - u_guess # flipping when both u and v are mostly negative

        guess_spectra.append(u_guess)

    return mcrar.fit(dat.T, ST=guess_spectra)

# Start the MCR analysis from the initial guess from SVD and the chosen number of components.
start_MCR(4, u, a)
# The 'good' exiting message is "Change in err below tol_err_change (9.8861e-06). Exiting."
```

Figure S58: Python script for multivariate curve resolution (MCR).

```python
from scipy.integrate import odeint
# Define the individual reaction steps that are to be modelled.
"""
Fe(II)-X + PhPAA -> Fe(IV)O + PhAA; k1

Fe(IV)O + Fe(II)-X -> 2 Fe(III)-X; k2

Fe(III)-X + PhPAA -> Fe(IV)O + CO2 + *CH2Ph; k3

Fe(IV)O + *CH2Ph -> Fe(III)-OPhCH2; k4

Fe(III)-OPhCH2 + CH3OH -> Fe(III)-OCH3 + HOCH2Ph; K4_2

Fe(III)-X + *CH2Ph -> Fe(II)-X + HOCH2Ph; k5

Fe(IV)O + CH3OH -> Fe(III)-OH + HOCH2*; k6
"""

# Rate constants that belong to the reaction steps above.
k1 = 10 # M^-1 . s^-1 estimated second order rate constant
k2 = 50 # M^-1 . s^-1 estimated second order rate constant
k3 = 18 # M^-1 . s^-1 estimated second order rate constant
k4 = 1e9 # M^-1 . s^-1 estimated diffusion controlled rate contstant in MeOH
k4_2 = 5 # s^-1 estimated pseudo first order rate constant
k5 = 10 # M^-1 . s^-1 estimated diffusion controlled rate contstant in MeOH
k6 = 2.1e-3 # s^-1 pseudo first order rate constant from literature

# A function that solves the differential equations and returns the concentrations vs time.
def reaction_PPAA(c, t):

    # Define the concentrations such that they are saved as an array.
    cFeII = c[0]
    cPPAA = c[1]
    cPAA = c[2]
    cFeIVO = c[3]
    cFeIII = c[4]
    cCO2 = c[5]
    cBenzyl_r = c[6]
    cBenzyl_alc = c[7]
    cFeIII_BA = c[8]


    # The differential equations defined
    cFeII_dt = - k1 * cFeII * cPPAA - k2 * cFeIVO * cFeII + k5 * cFeIII * cBenzyl_r
    cPPAA_dt = - k1 * cFeII * cPPAA - k3 * cFeIII * cPPAA
    cPAA_dt = + k1 * cFeII * cPPAA
    cFeIVO_dt = + k1 * cFeII * cPPAA - k2 * cFeIVO * cFeII + k3 * cFeIII * cPPAA - k4 * cFeIVO * cBenzyl_r - k6 * cFeIVO
    cFeIII_dt = + 2 * k2 * cFeIVO * cFeII - k3 * cFeIII * cPPAA + k4_2 * cFeIII_BA - k5 * cFeIII * cBenzyl_r + k6 * cFeIVO
    cCO2_dt = + k3 * cFeIII * cPPAA
    cBenzyl_r_dt =  + k3 * cFeIII * cPPAA - k4 * cBenzyl_r * cFeIVO - k5 * cFeIII * cBenzyl_r
    cBenzyl_alc_dt = + k4_2 * cFeIII_BA + k5 * cFeIII * cBenzyl_r
    cFeIII_BA_dt = + k4 * cFeIVO * cBenzyl_r - k4_2 * cFeIII_BA

    # Return the array of the concentrations over time
    return np.array([cFeII_dt, cPPAA_dt, cPAA_dt, cFeIVO_dt, cFeIII_dt, cCO2_dt, cBenzyl_r_dt, cBenzyl_alc_dt, cFeIII_BA_dt])

c0 = [1e-3, 100e-3, 0, 0, 0, 0, 0, 0, 0] # Initial concentrations of the compounds

t_in = np.linspace(0, 7500, 500000) # Time over which the calculation is done and the timestep

c = odeint(reaction_PPAA, c0, t_in) # Solving differential equations
c = c*1000 # Multiply by 1000 to get mM

# Define labels and colors
labels_model = ["Fe(II)-OMe", "PhPAA", "PhAA", "Fe(IV)O",
            "Fe(III)-OMe", "CO$_{2}$", "PhCH$_{2}$*", "PhCH$_{2}$OH", "Fe(III)-OCH$_{2}$Ph"]
colors_model = ["green", "darkorange", "cyan", "red",
            "blue", "purple", "magenta", "y", "black"]

# Mass balance check (should solve to 1)
conc_iron = (c[:, 0] + c[:, 3] + c[:, 4]+ c[:, 8])
print(conc_iron)
```

Figure S59: Python script for the initial kinetic model.

```python
#%% The function
# Define the individual reaction steps that are to be modelled.
"""
Fe(II)-X + PhPAA -> Fe(IV)O + PhAA; k1

Fe(IV)O + Fe(II)-OH2 -> 2 Fe(III)-OH; k2

Fe(III)-X + PhPAA -> Fe(IV)O + CO2 + *CH2Ph; k3

Fe(IV)O + *CH2Ph -> Fe(III)-OPhCH2; k4

Fe(III)-OPhCH2 + CH3OH -> Fe(III)-OCH3 + HOCH2Ph; K4_2

Fe(III)-HOCH3 + *CH2Ph -> Fe(II)-OCH3 + HOCH2Ph; k5

Fe(IV)O + CH3OH -> Fe(III)-OH + HOCH2*; k6

Fe(IV)O + CH3OH -> Fe(II) + CH2O; k7
"""
# Rate constants that belong to the elementary reaction steps above.
k1 = 10 # M^-1 . s^-1 estimated second order rate constant
k2 = 50 # M^-1 . s^-1 estimated second order rate constant
k3 = 18 # M^-1 . s^-1 estimated second order rate constant
k4 = 1e9 # M^-1 . s^-1 estimated diffusion controlled rate contstant in MeOH
k4_2 = 5 # s^-1 estimated pseudo first order rate constant
k5 = 10 # M^-1 . s^-1 estimated diffusion controlled rate contstant in MeOH
k6 = 2.1e-3 # s^-1 pseudo first order rate constant from literature
k7 = 6 # M^-1 . s^-1 estimated second order rate constant

# A function that solves the differential equations and returns the concentrations vs time.
def reaction_PPAA(c, t):

    # Define the concentrations such that they are saved as an array.
    cFeII = c[0]
    cPPAA = c[1]
    cPAA = c[2]
    cFeIVO = c[3]
    cFeIII = c[4]
    cCO2 = c[5]
    cBenzyl_r = c[6]
    cBenzyl_alc = c[7]
    cFeIII_BA = c[8]
    cCH2O = c[9]

    # The differential equations defined
    cFeII_dt = - k1 * cFeII * cPPAA - k2 * cFeIVO * cFeII + k5 * cFeIII * cBenzyl_r + k7 * cFeIVO
    cPPAA_dt = - k1 * cFeII * cPPAA - k3 * cFeIII * cPPAA
    cPAA_dt = + k1 * cFeII * cPPAA
    cFeIVO_dt = + k1 * cFeII * cPPAA - k2 * cFeIVO * cFeII + k3 * cFeIII * cPPAA - k4 * cFeIVO * cBenzyl_r - k6 * cFeIVO - k7 * cFeIVO
    cFeIII_dt = + 2 * k2 * cFeIVO * cFeII - k3 * cFeIII * cPPAA + k4_2 * cFeIII_BA - k5 * cFeIII * cBenzyl_r + k6 * cFeIVO
    cCO2_dt = + k3 * cFeIII * cPPAA
    cBenzyl_r_dt =  + k3 * cFeIII * cPPAA - k4 * cBenzyl_r * cFeIVO - k5 * cFeIII * cBenzyl_r
    cBenzyl_alc_dt = + k4_2 * cFeIII_BA + k5 * cFeIII * cBenzyl_r
    cFeIII_BA_dt = + k4 * cFeIVO * cBenzyl_r - k4_2 * cFeIII_BA
    cCH2O_dt = + k7 * cFeIVO

    # Return the array of the concentrations over time
    return np.array([cFeII_dt, cPPAA_dt, cPAA_dt, cFeIVO_dt, cFeIII_dt, cCO2_dt, cBenzyl_r_dt, cBenzyl_alc_dt, cFeIII_BA_dt, cCH2O_dt])

c0 = [1e-3, 100e-3, 0, 0, 0, 0, 0, 0, 0, 0] # Initial concentrations of the compounds

t_in = np.linspace(0, 800, 500000) # Time over which the calculation is done and the timestep

c = odeint(reaction_PPAA, c0, t_in) # Solving differential equations
c = c*1000 # Multiply by 1000 to get mM

# Define labels and colors
labels_model = ["Fe(II)", "PhPAA", "PhAA", "Fe(IV)O",
        "Fe(III)", "CO$_{2}$", "PhCH$_{2}$*", "PhCH$_{2}$OH", "Fe(III)-OCH$_{2}$Ph", "CH$_{2}$O"]
colors_model = ["green", "darkorange", "cyan", "red",
        "blue", "purple", "magenta", "y", "black", "lightgreen"]
```

Figure S60: Python script for the kinetic model in which an extra elementary step (k_7) is introduced in which **4** is reduced to **2a** by methanol to form formaldehyde.

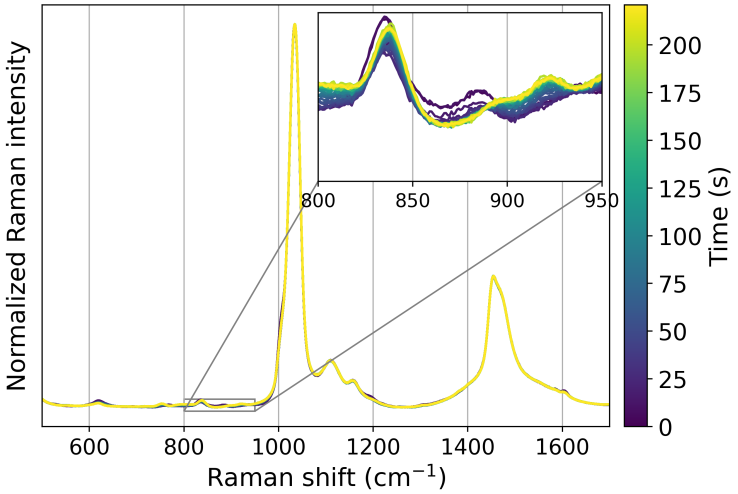

Figure S61: Raman spectra (liquid phase) recorded of after addition of 100 equiv. phenyl peracetic acid to **1** (1 mM) in methanol under an argon atmosphere.

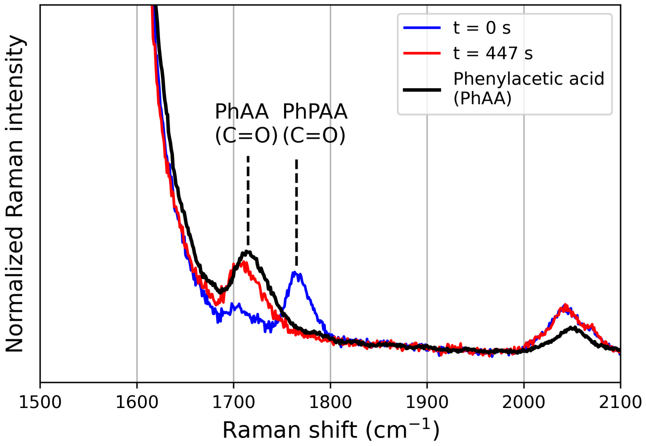

Figure S62: Raman spectra (liquid phase) recorded before (blue) and after (red) addition of 100 equiv. phenylperacetic acid to **1** (1 mM) in methanol under an argon atmosphere. A Raman spectrum of phenylacetic acid was recorded (black) for comparison.

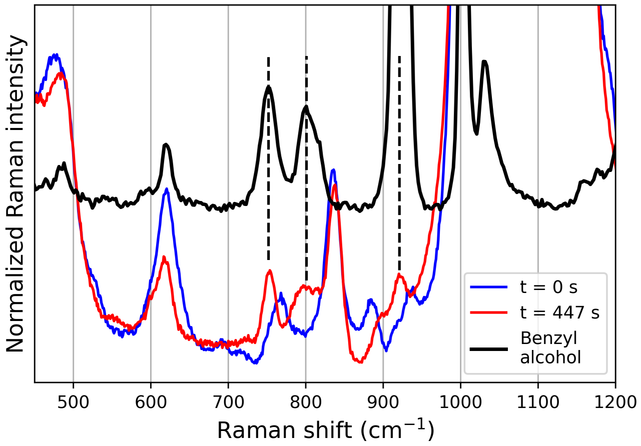

Figure S63: Raman spectra (liquid phase) recorded before (blue) and after (red) addition of 100 equiv. phenylperacetic acid to **1** (1 mM) in methanol under an argon atmosphere. A Raman spectrum of benzyl alcohol was recorded (black) for comparison.

Bond Dissociation Energies of PhPAA versus H_2O_2

BDEs were determined using DFT by calculating the starting molecule and products for the two following reactions:

$PhCH_2C(O)OOH \longrightarrow PhCH_2C(O)OO^{\cdot} + H^{\cdot}$

$HOOH \longrightarrow HOO^{\cdot} + H^{\cdot}$

All relevant molecules were optimized in the gas phase at the R2SCAN-3C level using Orca 6.0.0. The Gibbs Free energies were calculated by frequency calculations. And the BDE were calculated as by taking the different in Gibbs free energy of the products and the reactants.

	Single Point Energy (Eh)	Gibbs (Eh)
$H^{\cdot}$	-0.5	-0.50792602
HOOH	-151.53467184	-151.5298518
$HOO^{\cdot}$	-150.89685257	-150.90499706
PhPAA	-535.17235174	-535.06172692
$PhPAA^{\cdot}$	-534.51625216	-534.41911753

Table S1: Single point and Gibbs-free energies of relevant molecules.

	H_2O_2	PhPAA
BDE (GE) kcal/mol	73.4	84.5

Table S2: BDEs for H_2O_2 and PhPAA.
